# Canine Cognitive Dysfunction and Alzheimer’s Disease: Pathophysiological Relationships and the Impact of Glymphatic System Impairment on Neurodegeneration

**DOI:** 10.3390/vetsci13030298

**Published:** 2026-03-21

**Authors:** Maurizio Dondi, Ezio Bianchi, Paolo Borghetti, Rosanna Di Lecce, Giacomo Gnudi, Chiara Guarnieri, Valentina Buffagni, Francesca Ravanetti, Roberta Saleri, Attilio Corradi

**Affiliations:** Department of Veterinary Science, University of Parma, 43126 Parma, Italy; maurizio.dondi@unipr.it (M.D.); paolo.borghetti@unipr.it (P.B.); rosanna.dilecce@unipr.it (R.D.L.); giacomo.gnudi@unipr.it (G.G.); chiara.guarnieri1@unipr.it (C.G.); valentina.buffagni@unipr.it (V.B.); attilio.corradi@unipr.it (A.C.)

**Keywords:** canine cognitive dysfunction (CCD), Alzheimer’s disease (AD), neuroclinical signs (DISHAA), glymphatic system, β-amyloid, osmolytes, tau pathology, neurodegeneration, magnetic resonance imaging (MRI), translational model

## Abstract

Canine cognitive dysfunction (CCD) is a common age-related neurodegenerative disorder in dogs that shares several pathological and clinical features with human Alzheimer’s disease (AD). In both species, β-amyloid (Aβ) accumulates within the brain parenchyma and cerebral vessel walls and is associated with synaptic loss, oxidative stress, mitochondrial dysfunction, and chronic neuroinflammation, ultimately leading to progressive cognitive decline. Increasing evidence indicates that impairment of brain clearance mechanisms, particularly the glymphatic system, represents a central pathogenic mechanism in both CCD and AD. The glymphatic system is a glia-dependent perivascular network involved in the clearance of Aβ and other metabolic waste products from the brain. Its function declines with aging, vascular disease, and astrocytic alterations, including changes in aquaporin-4 distribution. Reduced glymphatic and periarterial drainage promotes the retention and aggregation of Aβ and tau proteins. Compared with AD, tau pathology in CCD is generally less extensive, supporting the interpretation of CCD as an Aβ-predominant condition and a partial pathological analog of Alzheimer’s disease. Clinically, CCD is characterized by a constellation of behavioral changes including, disorientation, altered social interactions, sleep–wake cycle disturbances, a loss of housetraining, changes in activity levels, and increased anxiety, commonly summarized by the DISHAA acronym. Overall, CCD represents a valuable spontaneous large-animal model for investigating neurodegenerative mechanisms and clearance-related therapeutic targets relevant to both veterinary and human medicine.

## Simple Summary

Canine cognitive dysfunction (CCD) is a common age-related neurodegenerative disorder in dogs and shares several pathological and clinical characteristics with human Alzheimer’s disease (AD). In both species, β-amyloid (Aβ) accumulates in the brain parenchyma and along cerebral blood vessel walls, where it is associated with synaptic loss, oxidative stress, mitochondrial dysfunction, and persistent neuroinflammatory processes, leading to a progressive decline in cognitive function. Growing evidence suggests that impairment of the glymphatic system is a key pathogenic mechanism in both CCD and AD. This glia-dependent perivascular network is involved in the clearance of Aβ and other metabolic by-products from the brain, and its function is reduced by aging, vascular disease, and astrocytic alterations, including changes in aquaporin-4 distribution. Reduced glymphatic and periarterial drainage promotes the retention and aggregation of Aβ and tau proteins. Compared with AD, tau pathology in CCD is typically less pronounced, supporting the interpretation of CCD as an Aβ-predominant condition and a partial pathological counterpart of Alzheimer’s disease. Clinically, CCD manifests as a spectrum of behavioral changes, including disorientation, altered social interactions, sleep–wake cycle disturbances, a loss of housetraining, changes in activity patterns, and increased anxiety, commonly summarized by the DISHAA acronym.

## 1. Introduction

Canine cognitive dysfunction is an age-related neurodegenerative syndrome increasingly recognized in companion dogs as lifespans lengthen. Affected animals develop progressive deficits in learning, memory, spatial orientation, social interaction, sleep–wake regulation, elimination behavior, and emotional control, which are commonly summarized by the DISHAA framework: disorientation, altered social interactions, sleep–wake disturbances, house-soiling, altered activity, and anxiety [1,2,3].

These clinical features closely resemble those observed in human Alzheimer’s disease, raising the question of whether canine cognitive dysfunction and AD should be considered distinct entities or, rather, species-specific manifestations of a shared pathogenic continuum [4]. Over the past three decades, neuropathological and molecular investigations have identified substantial convergences between CCD and AD. Both disorders are characterized by cortical and hippocampal atrophy, synaptic and neuronal loss, deposition of β-amyloid (Aβ) within the brain parenchyma and cerebral vessel walls in the form of cerebral amyloid angiopathy (CAA), increased oxidative stress, and persistent neuroinflammatory responses [2,5,6,7,8,9]. In aged dogs, Aβ plaques—predominantly enriched in Aβ42—accumulate spontaneously in the prefrontal and association cortices as well as in the hippocampus, and plaque burden correlates with the severity of cognitive impairment, closely mirroring observations in humans [5,10,11]. Tau pathology is generally less prominent in CCD and rarely progresses to the extensive neurofibrillary tangle (NFT) burden or stereotyped Braak-stage distribution typical of AD; nevertheless, tau abnormalities have been detected in a subset of CCD cases and appear to be mechanistically linked to Aβ deposition and associated neuroinflammatory and vascular alterations [12,13,14].

More recently, dysfunction of brain clearance mechanisms—particularly the glymphatic pathway and intramural periarterial drainage—has emerged as a central contributor to protein accumulation and neurodegeneration. The glymphatic system is a brain-wide, glia-dependent perivascular network that couples cerebrospinal fluid flow to interstitial fluid transport through astrocytic endfeet enriched in aquaporin-4 (AQP4), thereby facilitating the removal of metabolic waste products, including Aβ and tau [15,16]. Age-related vascular stiffening, CAA, astrocytic gliosis, and mislocalization of AQP4 disrupt these clearance pathways, reducing solute elimination and favoring the retention and aggregation of pathogenic proteins [17,18,19]. Anatomical, magnetic resonance imaging, and immunohistochemical studies indicate that dogs share the principal structural components of glymphatic and perivascular clearance systems with humans, including Virchow–Robin spaces, polarized perivascular AQP4 expression, osmolyte regulation, and basement membrane-based drainage routes [6].

Dogs therefore occupy a distinctive translational position between rodent models and humans. They are large-brained, long-lived, share the human domestic environment, and develop spontaneous age-related cognitive decline under natural conditions. Neuroimaging studies in CCD have demonstrated cortical and hippocampal atrophy, ventricular enlargement, reduced interthalamic adhesion thickness, white matter hyperintensities, and cerebral microhemorrhages, features that closely parallel established imaging biomarkers of AD [20,21,22,23,24]. At the ultrastructural level, transmission electron microscopy in both species reveals comparable amyloid fibrils, synaptic degeneration, mitochondrial abnormalities, and age-related myelin and axonal pathology [6,25,26,27].

Despite these similarities, important interspecies differences must be acknowledged, most notably the predominance of combined Aβ–tau proteinopathy in AD compared with a largely Aβ-centric pathology with relatively limited tau involvement in CCD, as well as distinct genetic architectures, such as the established roles of *APOE* ε4 and *PSEN1/2* mutations in humans versus the absence of clearly defined major genetic risk loci in CCD to date [4,13,28,29,30]. Accordingly, this review aims to (1) describe the anatomy and ultrastructural organization of glymphatic and perivascular clearance systems in dogs and humans; (2) summarize amyloid- and tau-related pathobiology, including relevant genetic and molecular factors, in CCD and AD; (3) compare the neuropathological, neuroimaging, and behavioral profiles of the two conditions; and (4) highlight the value of CCD as a spontaneous large-animal model for investigating glymphatic dysfunction, mixed proteinopathy–vascular mechanisms, and potential disease-modifying interventions relevant to human Alzheimer’s disease.

## 2. Anatomy of Glymphatic System

The glymphatic system is a brain-wide perivascular network that mediates cerebrospinal and interstitial fluid transport and facilitates metabolic waste clearance. Structurally, it is inseparable from the neurovascular unit, relying on the intimate anatomical coupling of vessels, perivascular spaces, and astrocytic glial cells. It has been described as a “glia-dependent lymphatic system,”, hence the term “glymphatic”, which is derived from the fusion of “glia” and “lymphatic” [12]. Although most mechanistic data derive from rodent and human studies, an increasing number of anatomical, imaging, and neuropathological investigations indicate that dogs possess a comparable glymphatic organization, supporting their use as a relevant large-animal translational model.

### 2.1. Cerebrospinal Fluid and Periarterial Central Nervous System

In anatomic detail, the glymphatic system originates at the interface between cerebrospinal fluid (CSF) in the subarachnoid space and the walls of cerebral arteries. Large pial arteries on the brain surface are surrounded by a sleeve-like fluid compartment, traditionally termed the Virchow–Robin space. As these arteries penetrate the cortical surface and enter the parenchyma, they remain surrounded by a perivascular space that is delimited externally by glial (astrocytic) structures and internally by the arterial wall [13,14].

**Figure 1:**
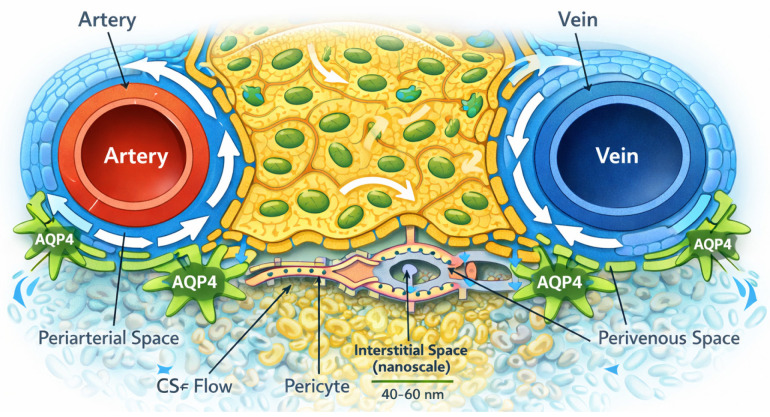
A schematic illustration of the glymphatic system. Cerebrospinal fluid (CSF) flows from the periarterial space through the interstitial compartment and drains into the perivenous space. Transport is mediated by AQP4 channels expressed on astrocytic endfeet surrounding the vasculature.

The wall of a penetrating artery is supported by associated basement membranes. Surrounding this vascular wall is a fluid-filled compartment that can receive CSF from the subarachnoid space. The outer boundary of this compartment is formed by astrocytic endfeet and a basal lamina that forms the outermost boundary of the central nervous system (CNS), the glia limitans. This structural arrangement creates a periarterial space in direct continuity with subarachnoid CSF and establishes a low-resistance pathway by which CSF can move into the brain parenchyma [12,13,14] (Figure 1).

Convective movement of cerebrospinal fluid along periarterial spaces is thought to be primarily driven by arterial pulsatility, with additional contributions from vasomotion and, to a lesser extent, respiratory and cardiac cycles [14]. In vivo two-photon imaging and particle tracking studies have demonstrated that arterial wall motion represents a major mechanical force underlying CSF transport within these compartments. Perturbations of vascular dynamics, such as those occurring in hypertension, markedly attenuate periarterial flow, underscoring the dependence of glymphatic inflow on intact arterial function [15]. From an anatomical standpoint, the periarterial compartment constitutes the principal “inflow limb” of a directional brain clearance circuit, whereby CSF enters the parenchyma along arterial pathways, disperses through the interstitial space, and is subsequently cleared along perivenous routes.

This arterial-to-venous polarity is a defining structural feature of the glymphatic pathway.

In dogs, Virchow–Robin (perivascular) spaces have been documented both histologically and by MRI, tracking penetrating arteries from the cortical surface into deep gray and white matter in a distribution closely resembling that described in humans [16]. On high-field MRI, these spaces appear as linear or ovoid T2-hyperintense foci aligned with the course of small penetrating vessels, particularly in the basal ganglia, thalamus and subcortical white matter. The number and caliber of these structures increase with advancing age and in association with presumed small-vessel disease or leukoaraiosis-like changes detected on MRI, supporting the interpretation that they represent dilated perivascular sleeves rather than lacunar infarcts. Although classical intravital two-photon tracer experiments, widely used in rodent models, have not yet been extensively performed in dogs, a growing body of experimental and clinical intrathecal contrast-enhanced MRI studies provides indirect functional evidence for periarterial glymphatic inflow in this species. Following intrathecal administration of gadolinium-based contrast agents or other tracers in research beagles and clinical canine patients, serial MRI typically demonstrates early enhancement of the basal cisterns and perivascular tracks surrounding major cerebral arteries, followed by delayed parenchymal and perivenous signal enhancement. This temporal pattern is consistent with CSF entry into periarterial sleeves, subsequent exchange with interstitial fluid, and drainage along perivascular pathways [17]. Taken together with the well-defined morphology of Virchow–Robin spaces in the canine brain, these findings support the presence of a functional periarterial compartment in dogs that likely contributes to glymphatic inflow in a manner analogous to that described in experimental rodent models.

### 2.2. Astrocytes, AQP4 Polarization, and the Perivascular Glial Sheath

Blood vessels provide the conduits, whereas astrocytes supply the critical perivascular lining that renders these conduits functionally glymphatic. Virtually all cerebral blood vessels are surrounded by astrocytic endfeet, which together form an almost continuous glial envelope known as the glial limitans [6].

A hallmark of this sheath is the high expression and polarization of aquaporin-4 (AQP4) water channels at the astrocytic endfeet facing the vessel wall [6] (Figure 1).

This polarization is not simply a biochemical feature but represents a critical determinant of glymphatic function. Aquaporin-4 (AQP4) enables rapid, bidirectional water exchange between the perivascular space and the interstitial compartment. A hallmark of the perivascular glial sheath is the polarized expression of AQP4 at astrocytic endfeet facing the vessel wall [6]. This polarization is considered a key structural determinant of efficient CSF–ISF exchange and glymphatic transport. From a structural perspective, the astrocytic perivascular sheath, together with its AQP4-enriched membranes, establishes a low-resistance interface between perivascular CSF and the brain interstitium [18,19]. Rather than relying exclusively on passive diffusion through the extracellular space, the glymphatic system leverages this highly water-permeable glial boundary to support convective, or “bulk,” fluid flow through the parenchyma [12,13,14]. The combined architecture of the perivascular basement membrane, astrocytic basal lamina, and overlapping astrocytic endfeet creates a specialized microenvironment in which variations in vascular tone, osmotic gradients, and extracellular solute composition can dynamically regulate both the direction and magnitude of fluid movement [20].

This astrocyte–vascular unit is also dynamic. In cerebral edema, trauma, and a range of neuropathologies, astrocytes undergo swelling, AQP4 expression and polarization are altered, and the vascular basal lamina may be disrupted [20]. These changes remodel the geometry and effective permeability of perivascular spaces, providing a mechanistic basis for the high vulnerability of glymphatic transport to both vascular and glial pathology [21].

Immunohistochemical studies in the canine brain show prominent AQP4 expression on astrocytic endfeet around blood vessels, ventricular ependyma, and the glia limitans, mirroring the distribution reported in humans and rodents [22].

In naturally occurring canine conditions such as meningoencephalitis, epilepsy, and brain tumors, perivascular AQP4 is upregulated or redistributed, and astrocytes become hypertrophic, indicating that the same astrocyte–vascular unit is engaged in fluid regulation [22].

In aged dogs with cognitive dysfunction and amyloid angiopathy [5,6,11], features of astrocytic gliosis and altered perivascular AQP4 expression were recognized [22].

### 2.3. Parenchymal Interstitial Pathways: From Periarterial Spaces to Perivenous Routes

Once CSF traverses the astrocytic perivascular boundary, it mixes with interstitial fluid within the brain extracellular space (ECS). Anatomically, the ECS consists of narrow channels and clefts between neurons, glial cells, and microvessels, and occupies approximately 15–20% of the nervous tissue volume in the healthy adult brain. Its geometric properties, particularly tortuosity (the degree to which diffusion pathways deviate from a straight line) and volume fraction, critically influence the dispersion of solutes and fluid within the brain parenchyma [12].

Tracer studies suggest net solute movement from periarterial inflow zones through the parenchyma toward perivenous efflux routes [13,14,23]. However, the relative contributions of bulk flow versus dispersion within the extracellular space remain debated. Anatomical arrangement—such as cellular packing density, myelinated fiber tracts, and regional variation in vascular density—strongly influences the spatial distribution and kinetics of solute movement [12,15].

### 2.4. Ultrastructural Anatomy of Glymphatic System

Transmission electron microscopy (TEM) defines the glymphatic system as an ultrastructural continuum comprising the nanoscale interstitial space, composite vascular basement membranes at capillary and arterial levels, smooth muscle cell basement membranes forming intramural drainage pathways, and the astrocytic glia limitans with polarized AQP4 expression (Figure 1). Together, these elements form a structurally integrated pathway that regulates fluid exchange, solute clearance, and metabolic homeostasis in the brain. At the capillary and arteriolar levels, the perivascular space is represented ultrastructurally by vascular basement membranes formed through the fusion of endothelial and astrocytic basal laminae of the glia limitans, creating a continuous nanoscale pathway that is contiguous with the interstitial extracellular space [24].

TEM studies demonstrate direct continuity between the narrow (≈40–60 nm), tortuous interstitial spaces and capillary basement membranes, supporting diffusion-dominant solute transport within the parenchyma and size-restricted access to intramural perivascular drainage pathways [25]. At the capillary level, the vascular wall is composed of a non-fenestrated endothelial cell layer joined by tight junctions, an underlying endothelial basement membrane, embedded pericytes, and an abluminal astrocytic sheath forming the glia limitans.

TEM reveals that the capillary basement membrane is a composite structure arising from the fusion of endothelial and astrocytic basal laminae, typically organized into a trilaminar architecture composed of two laminae rarae flanking a central lamina densa [25]. This basement membrane lies in direct focal continuity with the surrounding interstitial space, allowing small solutes to pass from the parenchyma into intramural perivascular drainage pathways while excluding larger particulate material, as demonstrated by size-restricted tracer and nanoparticle experiments.

As vessels transition from capillaries to arterioles and arteries, ultrastructural organization becomes increasingly complex. The tunica media of arterioles and arteries contains concentric layers of smooth muscle cells, each ensheathed by its own basement membrane. TEM studies show that intramural perivascular drainage of interstitial fluid and solutes occurs specifically within these smooth muscle cell basement membranes, which together form a longitudinal, three-dimensional network within the arterial wall that channels solutes out of the brain toward leptomeningeal and cervical lymphatic pathways [25].

At the level of penetrating cortical arteries, cerebrospinal fluid enters the brain along the pial–glial basement membrane, a specialized basal lamina shared between the pia mater and the astrocytic glia limitans. Electron microscopy demonstrates that this pial–glial basement membrane lies external to the smooth muscle layers and forms a narrow, continuous sheet without a true Virchow–Robin “space” in the classical sense [25]. TEM-based tracer studies further show that particles introduced into the CSF rapidly accumulate within this pial–glial basement membrane but do not penetrate directly into the interstitial space unless transferred across the astrocytic endfoot layer. Astrocytic endfeet constitute a critical ultrastructural interface in the glymphatic system. These specialized astrocytic processes envelop 60–98% of the cerebrovascular surface and collectively form the glia limitans. TEM reveals that adjacent endfeet are separated by narrow inter-endfoot clefts and that their perivascular membranes display a high density of aquaporin-4 (AQP4) water channels, reflecting pronounced molecular polarization [24]. This polarized AQP4 distribution is thought to support rapid, bidirectional water exchange between perivascular compartments and the interstitium, while maintaining size selectivity for solute movement.

Ultrastructural alterations of astrocytic endfeet—including mitochondrial swelling, disruption of cristae architecture, accumulation of autophagic vacuoles, and loss of normal endfoot organization—have been documented in human conditions associated with impaired glymphatic clearance, such as idiopathic normal pressure hydrocephalus, and correlates with reduced perivascular AQP4 expression [26].

Additional ultrastructural support for the existence of glymphatic transport pathways derives from electron microscopic studies localizing exogenous substances within the brain. Multimodal transmission electron microscopy and spectroscopic analyses have demonstrated the presence of insoluble gadolinium deposits within capillary and arteriolar basement membranes, the perivascular (Virchow–Robin) compartment, and the interstitial space. These deposits are frequently associated with folds of the basal lamina and astrocytic elements, while notably remaining absent from the endothelial cytoplasm, a distribution pattern consistent with solute transport along basement membrane-defined pathways rather than transendothelial passage [27].

## 3. Cerebrospinal Fluid, the Glymphatic System and the Aging Dog

Aging is a complex, multifactorial process that can be defined as a progressive biological decline that leads to a gradual reduction in the maintenance of the organism’s homeostasis. In dogs, given the wide variability of breeds and sizes, a senior dog is defined as one in the last third of its expected lifespan [28].

In dogs, twelve hallmarks have been proposed by researchers to highlight the main and significant changes related to aging [29,30]. In 2024, Guelfi et al. [31] proposed an additional indicator, hydration level, which directly impacts water balance, with effects primarily on the muscles, cardiovascular system, and the nervous system. In the latter, water plays an important role not only in the integrity of cell membranes and as a mediator for electrical signal transmission in neurons, but also as a major and fundamental component of cerebrospinal fluid. Cerebrospinal fluid (CSF) is a colorless fluid composed primarily of water (99%), with low concentrations of proteins, ions, neurotransmitters, and glucose [32].

Several hypotheses have been proposed to explain the mechanisms underlying the physiology of the cerebrospinal fluid, regarding its production and dynamics. The classical theory, known as the “Weed–Dandy–Cushing hypothesis” and defined over 100 years ago, identifies the choroid plexus as the main sites of CSF production (approximately 80%) and secretion and as the main regulator of flow. The choroid plexus consists of a vascularized stromal core containing fenestrated capillaries, covered by a single layer of specialized secretory epithelial cells. Because the endothelial cells of these capillaries lack tight junctions, they are highly permeable, allowing fluid and solutes to move from the bloodstream into the stromal compartment along hydrostatic and osmotic gradients. For this reason, the choroid plexus is one of the few regions of the central nervous system that lacks a functional blood–brain barrier [32]. In contrast, the epithelial cells are interconnected by tight junctions, forming a selective barrier that tightly regulates the passage of substances from the stroma into the ventricular system and thereby controls cerebrospinal fluid composition. This arrangement allows blood-derived substances to access the epithelial layer, where their transfer into the ventricular system is selectively regulated. The classical model of cerebrospinal fluid (CSF) physiology is centered on the choroid plexus and is based on three principal assumptions: (1) active CSF formation and secretion by the choroid plexus epithelium; (2) passive absorption of CSF at distal sites; and (3) a predominantly unidirectional flow from the ventricular system to the subarachnoid space. According to this theory, CSF production by the choroid plexus generates the pressure gradient that drives fluid movement from the ventricles toward the subarachnoid compartment.

According to this model, cerebrospinal fluid production is primarily driven by transcellular sodium (Na^+^) transport mediated by the Na^+^/K^+^-ATPase on the luminal membrane of choroid plexus epithelial cells. Sodium transport is accompanied by chloride (Cl^−^) and bicarbonate (HCO_3_^−^) fluxes, establishing osmotic gradients that facilitate the movement of water into the ventricular system. Aquaporin-1 mediates water channel transfer from the blood to the CSF [33]. By maintaining low intracellular Na^+^ concentrations, the Na^+^/K^+^-ATPase generates the electrochemical gradient that drives Na^+^ entry across the basolateral membrane, likely mediated by the Na^+^-dependent HCO_3_^−^ cotransporter NCBE and/or the Na^+^/H^+^ exchanger NHE1.

The importance of Na^+^ transport is further supported by the genetic deletion of Na^+^ transporters, which markedly reduce CSF production and ventricular size [34].

HCO_3_^−^ and Cl^−^ transport also play a significant role in CSF formation. It is proposed that intracellular accumulation of HCO_3_^−^ promotes its efflux via HCO_3_^−^ channels and the HCO_3_^−^/Cl^−^ exchanger AE2, leading to intracellular Cl^−^ accumulation and generation of a gradient that drives Cl^−^ secretion through apical Cl^−^ channels and transporters (i.e., NKCC1). The coordinated movement of Na^+^, Cl^−^, and HCO_3_^−^ from blood to ventricles establishes the osmotic gradient required for water transport [32].

Water movement across the choroid plexus epithelium occurs mainly through the highly permeable water channel AQP1, predominantly expressed in the apical membrane. Although its exclusivity as a water pathway is debated, AQP1 is critical for CSF production, as its deletion significantly reduces CSF secretion and epithelial water permeability [35]. Overall, the integrated transport of ions and water results in the formation of CSF, which contains low levels of protein and K^+^, higher concentrations of Na^+^, Cl^−^, and Mg^2+^, and approximately 99% water compared with plasma.

However, due to the discrepancies between classical theory and experimental evidence, Bulat, Orešković, and Klarica have proposed a new model of CSF hydrodynamics [36,37]. In this model, CSF is formed through fluid filtration across capillary walls, with CSF and interstitial fluid volumes regulated by hydrostatic and osmotic forces driven by protein and ion gradients. Water is filtered from high-pressure capillaries into interstitial fluid and CSF, while relatively impermeable electrolytes create osmotic counterpressure. As blood reaches low-pressure capillaries and venules, water is reabsorbed, resulting in a continuous exchange between CSF and interstitial fluid (ISF).

The discovery of the glymphatic system fully supported the hypothesis by Bulat, Klarica and Oreskovic [36]. In 2012, Iliff et al. provided experimental evidence supporting directional periarterial inflow and perivenous efflux, consistent with the anatomical framework described above [13]. The glymphatic system is an active, energy-dependent process driven by several mechanisms: continuous CSF production, respiration, and especially arterial pulsatility. The physiological regulation of the glymphatic system is complex and depends on multiple factors. Experimental studies have shown that arterial pulsatility is a key element: reducing the pulsation of cerebral arteries impairs the influx of CSF into the brain, whereas increasing pulsatility enhances glymphatic flow. Arterial pulsations promote the entry of CSF into perivascular spaces and facilitate exchange between CSF and interstitial fluid, explaining why glymphatic flow occurs primarily along arteries rather than veins [14]. Respiration and low-frequency vasomotor oscillations also contribute to cerebral fluid dynamics [38].

The state of arousal strongly influences the glymphatic activity. During sleep, CSF flow and the clearance of interstitial solutes, including β-amyloid, are increased. This effect is linked to reduced noradrenergic tone and expansion of the extracellular space, which facilitates fluid exchange [39]. In humans, body position during sleep also plays a role: the lateral position promotes more efficient clearance compared with supine or prone positions, highlighting the influence of postural and gravitational factors [40]. Functionally, the glymphatic system supports clearance of metabolic waste (including Aβ and tau) and contributes to nutrient and solute distribution within the brain [13,14].

In addition, the glymphatic system is crucial for nutrient distribution, including glucose delivery, for drug transport, and for intercellular communication.

As mentioned above, AD is characterized by the accumulation of amyloid-β (Aβ) and hyperphosphorylated tau, processes increasingly linked to impaired brain clearance mechanisms rather than overproduction alone [41]. In addition to promoting the clearance of substances to be eliminated, the glymphatic system also allows for the influx of glucose and other nutrients to neurons and astrocytes, as well as the transport of cholesterol and lipids.

Glymphatic flow also contributes to paracrine signaling and mechano-transduction by influencing astrocytic calcium activity and mechanically activating specific receptors, underscoring its integrated role in brain physiology.

**Figure 2:**
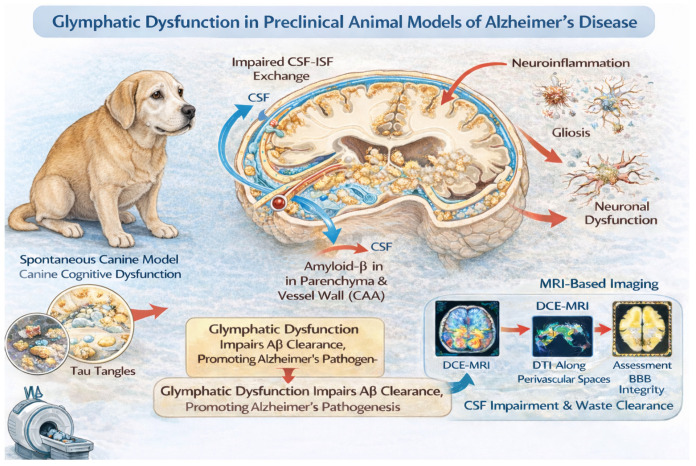
A schematic representation of the proposed role of canine cognitive dysfunction as spontaneous translational model for investigating Alzheimer’s disease pathogenesis and glymphatic system involvement.

Because patients with Alzheimer’s disease are believed to exhibit impaired cerebrospinal fluid dynamics, one pathogenic hypothesis proposes that reduced glymphatic function—and consequently diminished clearance capacity—contributes to the aggregation and accumulation of AD-related proteins [42,43,44]. The development of this model, which greatly expands knowledge, has unified previous physiological hypotheses on this fluid exchange and has also allowed for further exploration of its molecular aspects with the characterization of the role of aquaporin-4 (AQP4) at the cell level, astrocytes, and their structural and functional role in this system. Despite the promising progress achieved in this model, further and more detailed translational studies are required to elucidate how these alterations contribute to the pathogenesis of AD in humans [45]. Studies on preclinical models demonstrate a role of the glymphatic system in the clearance of β-amyloid (Aβ) and tau, thereby supporting the hypothesis that structural and/or functional dysfunction of this system contributes to the pathogenesis of AD [46]. However, human studies have not yet fully elucidated the role of glymphatic dysfunction in the development of AD and other neurodegenerative diseases [47]. Similarly, although no specific research has focused on the role of the glymphatic system in the pathogenesis of canine cognitive dysfunction (CCD), this may represent a new and interesting field for future research (Figure 2).

The role of the glymphatic system in canine cognitive dysfunction and its pathogenesis has not yet been investigated. However, like humans, dogs show neuropathological alterations associated with amyloid-β pathology, and the deposition of amyloid-β is progressive and characterized by four maturation stages, suggesting a gradual development of amyloid pathology similar to early phases of human AD [4,5,6,10]. Because these changes occur naturally and dogs develop age-related cognitive decline, the canine model provides a useful platform for studying early pathogenic events and for supporting the translation of research findings to human neurodegenerative diseases.

### 3.1. Aquaporin-4 Channels

The existence of a structured, convective transport system rather than simple diffusion has been demonstrated by in vivo imaging studies in mice. In brief, CSF enters the brain from the subarachnoid space driven primarily by arterial pulsatility, with additional contributions from respiration and pressure gradients. The fluid then penetrates the parenchyma through aquaporin-4 water channels, which are highly enriched in astrocytic endfeet, facilitating exchange between cerebrospinal and interstitial compartments.

This influx generates a convective ISF flow toward perivenous spaces, from which fluid and solutes are ultimately drained into the cervical lymphatic system [13,19]. Experimental studies in mice lacking the AQP4 gene, the α-syntrophin gene (Snta1), or subjected to pharmacological inhibition of AQP4 function have demonstrated the critical role of this channel and its perivascular astrocytic localization in glymphatic transport. Under these conditions, glymphatic function is markedly impaired, resulting in reduced CSF influx and diminished clearance of brain solutes [13,15,48]. Although AQP4 is a water-selective channel, the precise mechanisms by which it directly or indirectly facilitates solute clearance remain incompletely understood.

It is known that aquaporin-4 is the principal water channel in the central nervous system and is predominantly expressed in astrocytic processes, particularly in the perivascular endfoot membranes lining the cerebral vasculature [18,45,49]. AQP4 tetramers assemble into higher-order supramolecular structures known as orthogonal arrays of particles (OAPs) [50]. Two major isoforms of AQP4 are expressed, M1 and M23 [51]. The shorter M23 isoform forms the core of OAPs through N-terminal intermolecular interactions, while the longer M1 isoform is typically distributed at the periphery of these arrays. OAPs are highly enriched in perivascular astrocytic endfeet due to interactions with the dystrophin-associated protein complex (DAPC) [52]. Specifically, AQP4 binds to α-syntrophin, which links the channel to dystrophin and, via α-dystroglycan, to extracellular matrix proteins such as laminin and agrin within the perivascular glial basement membrane.

This highly organized molecular architecture gives rise to a dense concentration of aquaporin-4 channels at the interface between perivascular and interstitial compartments [53] (Figure 1 and Figure 2). The polarized localization of AQP4 minimizes the resistance to CSF–ISF exchange and thereby facilitates efficient fluid transport. According to this interpretation, AQP4 knockout mice show a marked reduction in CSF influx following the administration of fluorescent tracers into the cisterna magna when compared with wild-type controls.

Notably, while periarterial tracer entry remains largely intact, movement of tracers from periarterial spaces into the surrounding parenchyma is markedly impaired, suggesting that AQP4 specifically facilitates fluid transfer between perivascular spaces and the interstitial compartment [54]. A large body of evidence further supports the central role of astrocytic AQP4 in glymphatic transport [55].

Astrocytic endfeet cover more than 60% of the cerebral capillary surface. AQP4 regulates convective fluid movement along perivascular pathways continuous with the Virchow–Robin spaces [56]. Meta-analyses of AQP4 knockout mouse models consistently demonstrate reduced ISF and CSF tracer flux, confirming the importance of AQP4 in CNS fluid homeostasis. In addition to its perivascular localization, AQP4 is also expressed in ventricular ependymal cells, further contributing to global regulation of brain water balance [57]. The M23 isoform appears to play a dominant role in glymphatic function and astrocytic process motility.

Alterations in aquaporin-4 expression, isoform composition, or perivascular polarization have been implicated across a broad spectrum of neurological and neuropathological conditions, including neurodegenerative disorders, traumatic brain injury, cerebrovascular disease, and malignancies of the central nervous system.

Proper perivascular localization of AQP4 depends on the integrity of the dystrophin-associated complex, which stabilizes the neurovascular unit. Changes in DAPC gene expression have been associated with cognitive decline in dementia, and experimental disruption of DAPC components leads to mislocalization of AQP4 and impaired glymphatic function.

Moreover, AQP4 localization is dynamically regulated through calmodulin-dependent signaling pathways. Calcium influx via TRPV4 channels and downstream protein kinase signaling controls the trafficking of AQP4 from intracellular vesicles to the astrocytic plasma membrane [58].

Environmental and physiological stimuli, such as hypoxia and hypothermia, can enhance AQP4 surface expression [59], whereas pharmacological inhibition of calmodulin signaling has been shown to reduce cerebral edema and improve functional recovery in experimental injury models [60,61].

Glymphatic activity is also strongly influenced by circadian rhythms, with fluid transport markedly enhanced during sleep and under anesthesia. These rhythmic fluctuations correlate with sleep-dependent trafficking and expression of AQP4, and experimental disruption of circadian clock genes leads to the dysregulation of AQP4 localization and interstitial fluid flow.

Alvarez et al. [22] were the first to characterize the presence and distribution of AQP4 in the healthy canine brain, demonstrating a pattern closely comparable to that described in humans. AQP4 was widely expressed in astrocytic membranes, with prominent localization at perivascular endfeet along the blood–brain barrier. Age-related alterations in AQP4 distribution were observed in both gray and white matter. Moreover, dogs with idiopathic communicating hydrocephalus exhibited increased AQP4 concentrations in the cerebrospinal fluid, consistent with reports of AQP4 upregulation in pathological conditions associated with brain fluid overload, including internal hydrocephalus [62].

### 3.2. Role of Osmolytes in Alzheimer’s Disease and Canine Cognitive Dysfunction

Osmolytes are low-molecular-weight organic molecules that preserve cellular integrity by regulating water balance and cell volume in response to osmotic stress. So-called compatible osmolytes allow cells to adapt to changes in extracellular osmolarity without disrupting protein structure or cellular function. Major organic osmolytes include polyols (such as sorbitol and myo-inositol), amino acids and derivatives (taurine, glutamate, glycine), and methylamines (betaine and glycerophosphocholine) [63]. These molecules act as osmoprotectants and chemical chaperones, stabilizing protein conformation, preventing aggregation, and promoting refolding under denaturing or stress conditions [64,65,66]. In the brain, osmolytes play a crucial role in maintaining protein stability, ionic balance, and cellular homeostasis. They are particularly important for protecting neural cells against volume changes induced by hypo- or hyperosmotic conditions.

Efficient osmoregulation is essential for preventing cellular swelling or shrinkage and for preserving normal neuronal and glial function. Astrocytes play a central role in this process due to their high expression of the water channel AQP4, which facilitates transmembrane water movement and helps maintain the ionic and osmotic balance required for effective neuronal signaling [20,67]. Osmotic challenges that induce astrocytic swelling activate volume-regulated anion channels (VRACs), thereby promoting the efflux of chloride ions and organic osmolytes as part of the regulatory volume decrease (RVD) response.

The resulting osmotic gradients drive water efflux through AQP4, facilitating restoration of cell volume. Conversely, under hyperosmotic conditions, osmolyte uptake prevents excessive cellular dehydration. These tightly regulated osmolyte fluxes are essential for maintaining astrocyte morphology, cytoskeletal integrity, and perivascular endfoot structure [68,69,70].

Importantly, alterations in astrocyte morphology or cell volume can influence AQP4 localization, polarization and functional efficiency, further modulating glymphatic flow (Figure 2) [71].

Astrocytic swelling or impaired volume regulation can have significant consequences for brain fluid dynamics. Swollen astrocytic endfeet may narrow perivascular spaces, increasing resistance to cerebrospinal fluid (CSF)–interstitial fluid (ISF) exchange and ultimately reducing glymphatic clearance. Consequently, efficient osmoregulation is essential to protect cells from damage caused by swelling or shrinkage, to maintain cellular homeostasis and protein stability, and to enable both neurons and glial cells to adapt to osmotic stress. Furthermore, these mechanisms contribute to the regulation of fluid balance and ionic fluxes.

While AQP4 polarization, arterial pulsatility, and sleep are well-established regulators of glymphatic flow, the contribution of osmotic homeostasis and organic osmolytes remains comparatively underexplored [39,72].

Organic osmolytes are present in high concentrations in brain cytosol: polyols, such as myo-inositol or sorbitol, amino acids such as taurine, glutamate, aspartate, or glycine and methylamines, such as betaine or glycerophosphorylcholine. These compatible osmolytes regulate cellular water balance and are therefore thought to influence glymphatic fluid dynamics by modulating transmembrane water fluxes [73,74,75,76]. Astrocytes facilitate the exchange between cerebrospinal fluid (CSF) and interstitial fluid (ISF).

**Figure 3:**
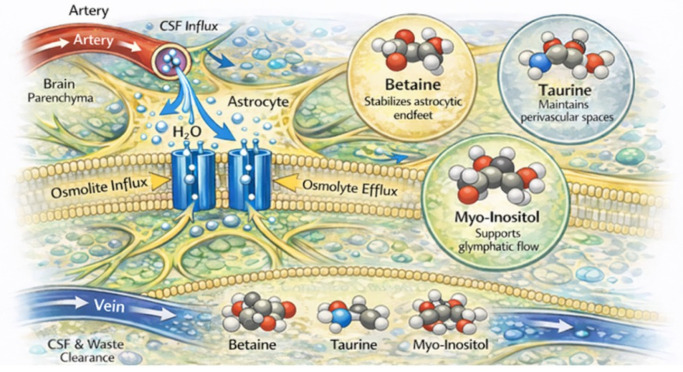
Organic osmolytes (i.e., betaine, taurine, and myo-inositol) are highly concentrated in astrocyte cytosol, where they regulate cellular osmotic balance and water exchange. Through aquaporin-4 (AQP4) water channels, astrocytes facilitate fluid movement between cerebrospinal fluid (CSF) and interstitial fluid (ISF), influencing glymphatic flow. Osmolyte influx and efflux help restore cell volume after osmotic stress, while their release under hypo-osmotic conditions limits excessive brain swelling.

The primary function of aquaporins (AQPs) as water channels underlies their role in cellular volume regulation, which notably involves the translocation of osmolytes across the cell membrane [54,77] (Figure 3).

Osmolyte fluxes enable the re-establishment of osmotic balance following anisosmotic stress; however, in pathological conditions, dysregulated osmolyte movement may itself contribute to brain cell swelling or shrinkage. During acute hypo-osmotic stress, after a rapid ion mobilization, organic osmolytes, glutamate, glutamine, betaine, creatine, and particularly taurine and myo-inositol, are released from brain cells, contributing to long-term cellular adaptation. This process accounts for an average reduction of approximately 50% in brain organic osmolyte content [78,79]. This loss is protective, limiting excessive water accumulation in the brain. Conversely, impaired osmolyte efflux leads to increased brain water content and is associated with severe neurological damage and increased mortality [80].

Accordingly, cell swelling elicits an adaptive response characterized by the activation of osmolyte fluxes driven by increased water permeability. Because water movement follows water potential, which is determined by osmotic gradients, aquaporin (AQP) activity and osmolyte transporters exert reciprocal influences [81].

These observations suggest that compatible osmolyte influx and efflux through astrocytic transporters may influence glymphatic efficiency by regulating astrocytic volume, perivascular space geometry, and water flux through AQP4.

Given that glymphatic clearance relies on unobstructed fluid movement through perivascular pathways, osmolytes such as betaine, taurine, and myo-inositol are thought to modulate glymphatic function and waste clearance, including amyloid-β and other metabolites, through their effects on cell volume, astrocytic endfoot integrity and stability, and osmotic gradients. Consequently, dysregulation of osmolytes may contribute to a glymphatic impairment in neurodegenerative diseases, particularly Alzheimer’s Disease. Moreover, chronic metabolic and inflammatory stress in AD may alter astrocyte volume regulation and potentially compress extracellular and perivascular spaces and impair clearance mechanisms. Although direct experimental studies linking osmolyte transporters to amyloid-β or tau clearance are still limited, osmolyte imbalance is increasingly recognized as a contributing factor to glymphatic insufficiency in AD. Ongoing research in AD on this topic may provide new insights into the pathogenesis and potential therapeutic strategies for cognitive dysfunction in dogs. Therefore, we discuss the functions of selected osmolytes and their transporters in the brain and explore their potential roles in the pathogenetic mechanisms of neurodegenerative disorders such as AD and CCD (Table 1).

#### 3.2.1. Betaine

In mammals, betaine (trimethylglycine) is distributed across multiple tissues, with the highest concentrations found in the kidney—particularly in the inner medulla, where it counteracts osmotic stress—and in the liver, where it plays a key role in methyl group metabolism.

Beyond its function as a methyl donor in the conversion of homocysteine to methionine, betaine is a major organic osmolyte that protects cells against osmotic stress. In addition, it stabilizes protein structure and prevents protein denaturation by acting as a chemical chaperone.

Betaine typically exerts an osmoprotective role in the kidney but is also present in the brain and in other tissues, including the eye, skin, endothelium, and cartilage, where activation of its transporter BGT-1 in response to osmotic stress has been described [63,82,83,84,85,86].

Betaine transport activity mediated by BGT-1 was first identified in Madin–Darby canine kidney (MDCK) cells [87].

Subsequent identification of a close homology between its nucleotide sequence and those of brain transporters for γ-aminobutyric acid (GABA) and norepinephrine led to its designation as betaine/GABA transporter 1 (BGT-1; SLC6A12) [88,89].

BGT-1 was later identified in the mouse [90] and human brain [91], as well as in the human liver [92] and kidney [93].

BGT-1 is generally considered the primary transporter responsible for betaine uptake in the brain; however, its precise localization within specific brain regions remains controversial, and the involvement of additional transporters has been suggested [94].

Nevertheless, several studies have demonstrated the expression of BGT-1 in both astrocytes and neurons, supporting a neuroprotective role for betaine in the central nervous system [95,96,97].

Betaine-mediated neuroprotection refers to a set of strategies and mechanisms that protect neuronal elements from damage associated with neurodegenerative disorders, such as Alzheimer’s disease: it acts as an osmoprotectant, a methyl donor regulating metabolism, and an antioxidant and anti-inflammatory agent.

Betaine regulates the GABAergic system by acting as a substrate for GABA transporters and by suppressing GABA transaminase activity, thereby supporting elevated brain GABA levels. These mechanisms suggest that the potentially beneficial effects of betaine in Alzheimer’s disease may be mediated, at least in part, through modulation of the GABA system [98,99].

An in vitro study on the conformational stability of amyloid-β demonstrated that betaine is able to preserve the peptide in its soluble form and counteract amyloid aggregation, suggesting a potential role in limiting amyloidogenic processes [100].

Betaine has also been shown to promote anti-inflammatory microglial phenotypes and inhibit pro-inflammatory signaling pathways in vitro, which may contribute to its protective effects in inflammatory brain conditions [101,102].

As mentioned in pathogenesis, both in AD and CCD, chronically activated M1 microglia and astrocytes upregulate pro-inflammatory cytokines, chemokines, and reactive oxygen and nitrogen species production, promoting further neurodegeneration. In LPS-activated microglial cells, betaine treatment is able to inhibit the TLR4/NF-κB pathways significantly reducing the production of pro-inflammatory cytokines and increasing the release of anti-inflammatory cytokines, demonstrating its ability to regulate the polarization of the microglial phenotype [103,104].

Betaine may suppress the NLRP3 inflammasome and related proteins with inhibition of pro-inflammatory cytokine levels [105].

In the central nervous system, betaine has been proposed to play a key role in preventing astrocyte swelling. Knight et al. [97] demonstrated that hippocampal slices are capable of accumulating betaine and that its presence modulates the levels of other osmolytes under hyperosmotic conditions, supporting a protective, volume-stabilizing role for this molecule. Acting as a biocompatible osmolyte, betaine may contribute to preserving the structural stability of the terminal processes (endfeet) of AQP4-expressing astrocytes. Consistent with these findings, betaine accumulation has been shown to influence the intracellular concentrations of other major osmolytes, including taurine, creatine, and myo-inositol, particularly during hyperosmotic stress. Together, these observations suggest that betaine does not act in isolation but instead contributes to the coordinated regulation of the cellular osmolyte network in response to osmotic stress in neural tissue [97].

In this context, betaine could also influence water homeostasis and glymphatic function, which are essential for the exchange between cerebrospinal fluid and the interstitial compartment, as well as for the clearance of metabolic waste products. Recent experimental work in rat models of post-traumatic syringomyelia showed that astrocytes take up betaine via BGT-1 under hypertonic conditions, and that this uptake is directly linked to the regulation of cell volume. Importantly, pharmacological inhibition of BGT-1 diminishes this protective effect, supporting a direct involvement of BGT-1-mediated betaine transport in astrocyte osmoregulation [106].

Considering the emerging evidence supporting its neuroprotective and antioxidant roles, there remains a clear need to expand detailed molecular and cellular knowledge of betaine and the BGT-1 transporter in order to substantiate their potential therapeutic use [107].

The role of betaine as an organic osmolyte in response to osmotic stress, and as a methyl donor within one-carbon metabolism, where it remethylates homocysteine to methionine via betaine-homocysteine methyltransferase (BHMT), is biologically relevant across species, including companion animals such as dogs [108].

Unfortunately, there are few direct studies on the effects of betaine in the canine brain. Moreover, CNS distribution and transport mechanisms, such as the betaine–GABA transporter BGT-1, are poorly characterized in dog brain tissue compared with other tissues. Other reports have examined the role of betaine in canine nutrition in terms of systemic metabolism, including its detoxification function, influence on lipid metabolism, and effects on the canine metabolome and immune response [109,110].

While direct research on betaine’s effects on canine brain function remains very limited, a well-established theoretical framework from comparative mammalian physiology suggests that methyl donors such as betaine can support brain health. Most evidence is derived from studies of systemic metabolism or extrapolated from rodent and other mammalian models, indicating its putative role as an osmolyte contributing to cellular resilience under metabolic stress, as well as an antioxidant and detoxifying agent [101,111,112,113,114].

Betaine is commonly included in commercial dog foods as a methyl donor and osmolyte, potentially supporting metabolic health and nutrient utilization. Although no formal dietary requirement for betaine has been established in dogs, balanced levels of methyl donors, including choline and betaine, are considered beneficial for overall metabolic function and may also support neurodevelopmental processes [115].

#### 3.2.2. Taurine

Taurine is a sulfur-containing amino acid synthesized endogenously from cysteine and widely distributed across various tissues in the body. It exerts significant cytoprotective effects, largely attributed to its antioxidative and anti-inflammatory properties.

Similarly to humans, taurine is generally considered conditionally essential in dogs because it can be synthesized from sulfur-containing amino acids such as methionine and cysteine; however, dietary intake and metabolic factors can influence systemic taurine levels. In companion animals, including dogs and cats, taurine deficiency has been associated with serious health issues, notably primary taurine-deficiency-related dilated cardiomyopathy [116,117,118].

Taurine is particularly abundant in the central nervous system where it is present in various brain regions such as the cortex, cerebellum, and hippocampus. In these regions, taurine contributes to multiple aspects of brain function, supporting neuronal homeostasis, neurotransmission, and cytoprotection [119,120].

Taurine is especially important during brain development, where it plays a crucial role by supporting the proliferation and survival of neural progenitor cells and acting as a trophic factor for their differentiation and maturation [121,122,123].

Taurine’s protective effects have been reported in a wide range of pathological conditions involving the cardiovascular, respiratory, muscular, and endocrine systems [124,125,126,127].

In the central nervous system, taurine, owing to its intrinsic neuroprotective properties, represents a promising therapeutic strategy for the management of neurodegenerative disorders, including Alzheimer’s disease.

Taurine supplementation may offer protective effects against multiple biochemical alterations associated with Alzheimer’s disease pathophysiology [128].

Numerous studies have highlighted the role of taurine in the central nervous system, demonstrating neuroprotective, antioxidant, and cytoprotective effects in animal models of Alzheimer’s disease, in which taurine supplementation improves cognitive function [105,129,130,131].

Indeed, since it is able to neutralize free radicals, taurine protects cells during oxidative stress or from the induction of apoptosis during exposure to toxic agents [132,133,134,135].

Furthermore, taurine contributes to neuronal homeostasis and neuroprotection by regulating osmotic balance, exerting antioxidant and membrane-stabilizing effects, and by providing protection against neurotoxic agents [136,137,138,139]. Some studies highlight how the neuroprotective and antiapoptotic effect may be mediated by the PI3K–Akt pathway [140,141].

Taurine supplementation has also been suggested to mitigate neuroinflammation. Taurine modulates microglial activation and pro-inflammatory signaling and enhances expression of microglial receptors associated with improved clearance of pathological proteins [101,142,143,144,145].

Neuroprotective and osmotic effects of taurine are mediated via taurine transporters (TauT/SLC6A6) and a sodium- and chloride-dependent membrane protein [146,147].

Taurine can also transport β-alanine and GABA and is highly expressed in placenta and skeletal muscle and moderately expressed the heart, brain, lung, kidney, and pancreas; notably the retina and leukocytes have high taurine demands [148].

Taurine is particularly enriched in astrocytes, the primary site of taurine synthesis and metabolism, where it is rapidly released or accumulated in response to osmotic challenges, thereby contributing to the maintenance of intracellular osmotic balance and cell volume. Importantly, direct astrocyte–neuron crosstalk has been demonstrated to regulate taurine availability and to fine-tune its neuroprotective actions [149,150].

Vitvitsky et al. [151] demonstrated the presence of an intact taurine synthesis pathway both in neurons and astrocytes and that both cells in vitro respond to hypertonic conditions with an increase in taurine synthesis.

Consistently, under hypo-osmotic stress, taurine is released through volume-regulated anion channels, facilitating regulatory volume decrease, whereas under hyperosmotic conditions its uptake contributes to the prevention of cellular dehydration. Neural/stem progenitors (NCPs) undergo important changes in cell volume during proliferation and growth; NCPs show the ability to respond to hypotonicity by increasing taurine efflux similarly to other neural cells [152].

Taurine contributes to the regulation of neuronal water content during ion fluxes associated with membrane depolarization and synaptic transmission [153]. As a neuro-osmolyte [154], it also plays a key role in maintaining astrocytic volume and homeostasis by stabilizing astrocyte volume, supporting the structural integrity of astrocytic endfeet, and preserving the proper localization of AQP4 under osmotic stress [150,155]. By preventing astrocyte swelling, taurine may help maintain the patency of the perivascular space and thereby facilitate efficient glymphatic clearance of waste products from the brain parenchyma, including amyloid-β and other metabolites. In AD, where astrocytes are exposed to chronic metabolic and inflammatory stress, insufficient taurine-mediated volume regulation may contribute to extracellular space compression and impaired glymphatic clearance. Alteration of taurine homeostasis has been associated with cerebral edema, ischemia, and neurodegenerative conditions, highlighting its relevance to brain homeostasis and neuroprotection [81].

Furthermore, taurine plays an important role in modulating glutamate and GABA neurotransmission and prevents excitotoxicity in vitro primarily through modulation of intracellular calcium homeostasis. Taurine supplementation prevents age-dependent declines in cognitive functions [128,139]. In conclusion, several in vitro, preclinical, and in vivo studies have highlighted the neuroprotective effects of taurine in the brain, which are mediated through multiple mechanisms, including (i) the maintenance of cellular energy homeostasis; (ii) the modulation of intracellular calcium signaling, osmoregulation, and cell volume regulation; (iii) protection against glutamate-induced excitotoxicity; and (iv) antioxidant effects. While most studies on brain aging have been conducted in rodents, the neuroprotective properties of taurine, such as the reduction in oxidative stress and support of cell survival and osmoregulation, may also be relevant in dogs in the context of age-related cognitive decline. Although direct studies in dogs remain limited, evidence from comparative nutrition research suggests that taurine and other amino acid metabolites play important roles in brain development, cognitive function, and behavioral regulation. Diets in dogs that are adequate in functional amino acids, including taurine, may support structural and neurotransmitter integrity of the CNS, potentially influencing cognitive health and mood; however, specific canine data remain limited and require further investigation [156,157]. Further research should clarify whether supplementation benefits dogs beyond standard nutritional adequacy, especially in aging or disease models.

#### 3.2.3. Myo-Inositol

Myo-inositol (MI) is a cyclic polyalcohol (cyclitols) and a key precursor for phosphatidylinositol signaling molecules: inositol phosphates (IPs), phosphatidylinositols (PIs or PtdIns), and their phosphorylated derivatives (phosphatidylinositol phosphates, PIPs). Myo-inositol (MI) in mammals, including dogs, can be synthesized endogenously from glucose-6-phosphate [158].

Myo-inositol is a compound present in almost all tissues, including the kidney, liver, brain, lungs, heart, and skeletal muscle. In these tissues, it acts mainly as a compatible organic osmolyte especially in the medulla of the kidney, but also it participates in general cellular functions. Studies in both humans and dogs have confirmed that the mechanism of myo-inositol accumulation and regulation is conserved across species, demonstrating its crucial role as an osmolyte in various tissues. Furthermore, myo-inositol (MI) is a key regulator of cellular signaling and participates in numerous processes, including membrane biogenesis, structure and function, osmoregulation, ion channel modulation, mRNA export, stress responses, cytoskeletal dynamics, apoptosis, and metabolic homeostasis, including glucose and cholesterol metabolism [159].

MI uptake into cells is mediated by two main types of transporters, sodium-coupled (SMIT1/SMIT2) and proton-coupled (HMIT1), with the highest expression observed in the brain [160,161,162].

While direct canine-specific neuroscience research on myo-inositol is limited, studies in other mammals provide insights into its likely mechanisms of action in the brain:

1. Osmoregulation in the brain: MI acts as a compatible osmolyte in neural tissue, aiding neurons and astrocytes in adapting to osmotic stress and contributing to the regulation of cell volume [81].
2. Signal transduction, neurotransmission, and neurobehavioral effects: inositol derivatives participate in pathways that modulate serotonin, dopamine, noradrenaline, and acetylcholine neurotransmission, processes central to mood regulation, cognition, and stress responses [163].
3. Neuronal connectivity and synaptic maturation: in non-canine models, including mice, rats, and human neurons, MI has been shown to promote synaptic connectivity by enhancing excitatory synapse density and postsynaptic structure, suggesting a role in synaptic development and neuronal network formation [164].

Furthermore, several reports indicate that inositol plays important roles during phenotypic transitions and developmental phases, influencing fetal development, neural differentiation and function, reproductive function, and osteogenesis.

Dysregulation of inositol metabolism has been implicated in several chronic conditions. Accordingly, inositol has been investigated as a therapeutic adjunct in a range of disorders, including gynecological diseases, respiratory distress syndrome, Alzheimer’s disease, metabolic syndrome, and certain cancers [165].

Myo-inositol is highly concentrated in the brain, particularly in astrocytes. Within the central nervous system, MI serves as a substrate for PI3K (phosphoinositide 3-kinase)–Akt and PLC (phospholipase C) signaling pathways, which are critical for the regulation of neuronal survival, calcium homeostasis, synaptic plasticity, and astrocyte–neuron communication. Alterations of these pathways have been implicated in Alzheimer’s disease and other neurodegenerative disorders [166,167,168].

Moreover, MI is a key cerebral osmolyte involved in the regulation of cell volume and an astrocyte-enriched metabolite, with its elevated levels reflecting increased astrocyte density and activation [169].

Indeed, MI occupies a unique position in AD research. Elevated myo-inositol levels in the brains of patients with Alzheimer’s disease compared with cognitively healthy controls have been consistently observed in proton magnetic resonance spectroscopy studies, supporting its role as an early metabolic marker of AD pathology [170,171].

Higher MI levels are also seen in adults with Down’s syndrome and it has been shown that these patients are at high risk of developing AD [172]. High MI levels are also detected in presymptomatic patients and those with mild cognitive impairment (MCI), which indicates the possibility of using MI as a marker of the early stages of AD and mild cognitive impairment [169].

In 4- to 8-year-old dogs with varying cerebrospinal fluid Aβ concentrations, metabolomic analyses revealed increased myo-inositol levels, alongside other metabolic alterations in CSF [173].

Furthermore, increased levels of myo-inositol (MI) appear to be associated with a compromised antioxidant response in the pathogenesis of Alzheimer’s disease. It has been reported that MI inhibits catalase activity, thereby disrupting the balance between oxidant and antioxidant systems and exacerbating oxidative stress [174].

In patients with Alzheimer’s disease, alterations in inositol metabolism at multiple levels contribute to the disruption of neuronal Ca^2+^ homeostasis. Because calcium regulates essential processes ranging from cell growth to apoptosis, its dysregulation can lead to profound cellular dysfunction, ultimately resulting in cell death and contributing to neurodegeneration [175].

Conversely, other studies have reported beneficial effects of MI, suggesting a potential neuroprotective role. Both scyllo-inositol and myo-inositol have been shown to inhibit Aβ production and reduce amyloid plaque burden in the brains of transgenic mouse models of Alzheimer’s disease [176]. In contrast, in vitro studies examining the folding and aggregation propensity of tau protein have indicated that myo-inositol may promote tau aggregation [177].

In the human brain, myo-inositol is the main contributor to volume control in hyponatremia [178]. Increased myo-inositol levels may initially reflect an adaptive osmotic response; however, their persistent elevation could be associated with alterations in astrocytic volume regulation, including hypertrophy and changes in astrocytic endfoot morphology. These alterations may coincide with modifications in aquaporin-4 localization and function and may be observed in the context of reduced glymphatic system efficiency.

From this perspective, dysregulation of myo-inositol may represent a potential biomarker and may be linked to glymphatic dysfunction in Alzheimer’s disease.

Taken together, available evidence suggests a dual, adaptive versus maladaptive, role of myo-inositol in Alzheimer’s disease. Under normal conditions, MI could support neuronal function and resilience by enabling astrocytes to maintain homeostasis. In preclinical AD, elevated MI might reflect early astrocyte activation around Aβ deposits potentially representing a compensatory glial response prior to overt neuronal loss or cognitive decline.

In contrast, in established AD pathology, myo-inositol serves as a marker of chronic gliosis and neuroinflammation, possibly reflecting a loss of astrocytic functions, and oxidative imbalance. At this stage, MI may no longer exert protective effects, instead representing a signature of glial dysfunction.

Although direct neurobiological studies of myo-inositol in dogs are limited, research in other mammals supports its involvement in neural connectivity and glial function. In dogs, MI has been detected in both brain tissue and cerebrospinal fluid and is primarily synthesized within the brain, with minimal uptake from plasma, indicating tight local regulation of cerebral inositol pools [158].

As previously mentioned, a study on metabolomic profiling has identified increased myo-inositol in brain tissue associated with elevated cerebrospinal amyloid β (Aβ) concentrations in dogs, even before amyloid deposition occurs, suggesting that MI changes may reflect early metabolic stress and glial activation [173].

Moreover, the neuroprotective effects of myo-inositol are likely important for canine brain function, potentially supporting cognition, mood regulation, and stress resilience through modulation of neurotransmitter pathways, particularly serotonergic signaling. Aging dogs often exhibit alterations in neurotransmission and synaptic integrity; therefore, MI supplementation may enhance neuronal signaling and membrane dynamics, thereby contributing to the maintenance of cognitive function.

Despite a few studies investigating the effects of diet on aging and cognitive dysfunction in dogs [179,180,181,182], formal studies demonstrating the cognitive benefits of myo-inositol supplementation in dogs are still lacking.

Collectively, betaine, taurine, and myo-inositol may represent key components of the cerebral osmoregulatory system, with functions extending beyond cellular volume regulation; in addition to their specific metabolic roles, they show neuroprotective effects in several preclinical models of Alzheimer’s disease (AD), supporting their ability to stabilize protein conformation, limit amyloid-β aggregation, modulate tau-related pathways, attenuate oxidative stress, regulate intracellular calcium homeostasis, and influence microglial and astrocytic inflammatory responses, while also indirectly sustaining glymphatic clearance mechanisms—central to AD pathophysiology—by preserving astrocytic volume regulation and aquaporin-4-dependent water fluxes.

Although mechanistic and preclinical AD studies support osmolyte-mediated effects on protein aggregation, neuroinflammation, and astrocyte-dependent clearance pathways, direct investigations in dogs—particularly in CCD—remain scarce. Given the conserved nature of osmolyte transport systems and astrocyte physiology across mammals, controlled canine studies integrating diet, imaging, and biomarker endpoints are needed to determine whether osmolyte-based strategies can support brain resilience during aging and cognitive decline. While alterations in metabolites such as myo-inositol have been detected in aged dogs with amyloid-related changes [173], and taurine and betaine are acknowledged as metabolically relevant in canine physiology [115,116,117,118], controlled studies specifically addressing their role in canine brain aging are still lacking.

The convergence of data from rodent models and human studies strongly supports the biological plausibility that osmolyte-mediated mechanisms may also operate in the canine brain. Considering the conserved nature of osmolyte transport systems and astrocyte physiology across mammalian species, it is reasonable to hypothesize that similar protective pathways could be harnessed in dogs.

Therefore, a critical appraisal of preclinical data derived from AD models may support the emerging hypothesis that these compounds may contribute to neuroprotection, enhanced cellular resilience, and modulation of behavioral responses in dogs.

Taken together, these considerations indicate that betaine, taurine, and myo-inositol warrant evaluation within canine nutritional strategies designed to support optimal central nervous system (CNS) health, especially during aging or in pathological conditions such as CCD.

Therefore, advances in mammalian research may provide a valuable framework for expanding investigations in dogs, particularly in the context of canine cognitive dysfunction. Expanding research in this direction may not only advance therapeutic approaches for CCD but also strengthen the translational bridge between veterinary and human neurodegenerative medicine, supporting the development of mechanism-based nutritional interventions aimed at promoting brain resilience during aging.

**Table 1:** Roles of major organic osmolytes in brain: taurine, betaine, and myo-inositol.

Osmolyte	Main Cellular Localization	Primary Transporters	Osmoregulation Function	Additional Neurobiological Roles	Alterations in Brain Pathology	Key References
Taurine	Astrocytes > neurons	TauT (SLC6A6)	Major neuro-osmolyte regulating intracellular osmotic balance; mediates regulatory volume decrease under hypo-osmotic stress and prevents cellular dehydration under hyperosmotic conditions; stabilizes astrocytic volume and perivascular space integrity and may support of glymphatic clearance	Neuromodulation (modulate GABAergic signaling); Ca^2+^ homeostasis, membrane stabilization, antioxidant and cytoprotective effects; trophic factor during brain development; anti-inflammatory modulation of microglial activation;	Altered taurine homeostasis is associated with cerebral edema, ischemia, and neurodegeneration; increased vulnerability to excitotoxicity insufficient taurine-mediated volume regulation may contribute to impaired glymphatic clearance in AD; canine data are limited but suggest relevance for age-related cognitive decline	[81,105,128,129,135,136,156,183]
Betaine	Astrocytes and neurons (region-dependent)	BGT-1/GAT2 (SLC6A12)	Compatible organic osmolyte involved in cell volume regulation, particularly under hyperosmotic stress; prevents astrocyte swelling and supports perivascular space integrity	Methyl donor for remethylation of homocysteine; chemical chaperone for protein conformation; modulation of oxidative stress, neuroprotection, regulation of microglial polarization; potential support of astrocytic endfeet integrity and AQP4-related water homeostasis and of glymphatic function modulation of GABAergic neurotransmission	Evidence suggests neuroprotective effects in AD models, including inhibition of amyloid-β aggregation, suppression of inflammasome signaling, promotion of anti-inflammatory microglial phenotypes, and modulation of GABA metabolism; reduced transport or dysregulation may impair glymphatic clearance and exacerbate neuronal stress; data in dogs are scarce, but metabolomic and comparative physiology studies support a putative protective role in cognitive dysfunction	[63,82,84,87,95,97,98,99,100,107]
Myo-inositol	Predominantly astrocytes	SMIT1 (SLC5A3); SMIT2 (SLC5A11) HMIT1 (SLC2A13)	Slow but sustained osmoadaptation; accumulation during chronic osmotic stress	Precursor of phosphoinositides, intracellular signaling, synaptic connectivity, neurotransmission, and osmoregulation A of astrocyte markers in neuroimaging	Elevated levels in Alzheimer’s disease reflect gliosis and osmotic stress, afailure to resolve inflammation, a loss of normal astrocytic support functions, and imbalance of the oxidant/antioxidant system. Altered MI functions may influence glymphatic dysfunction	[81,156,159,167,168]

## 4. Pathogenesis

A central question in comparative neuropathology is whether canine cognitive dysfunction and human Alzheimer’s disease represent distinct entities or points along a shared continuum of age-related neurodegeneration [184]. Increasing histopathological and molecular evidence supports the continuum model: both conditions feature progressive amyloid deposition and tau pathology, with diffuse and neuritic Aβ plaques and hyperphosphorylated, misfolded tau emerging in analogous cortical and hippocampal networks, albeit with species-specific differences in lesion burden, distribution, and clinical expression [4,5,6,185,186].

### 4.1. Genetics

In humans, most AD cases are sporadic late-onset Alzheimer’s disease (LOAD) with multifactorial and largely unknown etiology [187]. A minority of cases represent early-onset familial AD, which is driven by highly penetrant mutations in genes directly involved in Aβ production, most notably APP and the presenilins PSEN1 and PSEN2 [187,188]. For LOAD, the APOE ε4 allele is the major genetic risk factor, with additional contributions from variants in TREM2 and multiple loci identified by genome-wide association studies (e.g., CLU, CR1, PICALM, BIN1, ABCA7, CD33, SORL1), although the mechanistic links between many of these loci and AD pathogenesis remain incompletely defined [189,190]. APOE4 is of particular interest because it is unique to humans and may have arisen from an ancestral primate APOE form in a way that facilitated susceptibility to AD [191,192,193].

By contrast, no specific mutations or risk loci have been robustly linked to CCD or canine CAA, and potential genetic predispositions remain largely undefined [4]. DNA sequence variants in numerous genes have been associated with a range of progressive neurodegenerative and neurodegenerative-like diseases in dogs, including degenerative myelopathy, neuronal ceroid lipofuscinoses, leukodystrophies, and lysosomal storage disorders—such as GM1 and GM2 gangliosidoses and mucopolysaccharidoses [194,195,196,197,198,199,200,201,202,203]; variants in some of these genes are also associated with progressive cognitive decline, although the underlying mechanisms likely differ from those involved in CCD.

### 4.2. Amyloid Deposition and Amyloidogenic Processing

Both CCD and AD are characterized by aberrant processing of amyloid precursor protein (APP) along the amyloidogenic pathway, resulting in accumulation of β-amyloid (Aβ) peptides. Under non-pathological conditions, APP is preferentially cleaved by α-secretase within the Aβ domain, thereby precluding Aβ formation. In aging brains, a shift toward β-secretase (BACE1) and γ-secretase cleavage generates Aβ_1_–_40_ and Aβ_1_–_42_, with Aβ_1_–_42_ being more aggregation-prone and neurotoxic [204].

Longer Aβ species, such as Aβ_42_, exhibit a greater propensity to aggregate into fibrils and plaques compared with Aβ_40_, and the plasma Aβ_42_/Aβ_40_ ratio has therefore emerged as an important biomarker of early Alzheimer-related brain changes and disease progression. In dogs, both diffuse and compact Aβ plaques are found predominantly in the prefrontal cortex, hippocampus, and association cortices—regions that closely correspond to those affected in human AD [5]. The regional distribution of plaques in CCD correlates with impairments in spatial learning and executive function, paralleling patterns observed in humans [205,206]. Increasing evidence indicates that soluble oligomeric Aβ species, rather than fibrillar plaques alone, are the principal mediators of synaptic toxicity. These oligomers interfere with synaptic plasticity, disrupt long-term potentiation (LTP), alter NMDA receptor signaling, and perturb intracellular calcium homeostasis [207,208]. Consistent with this view, increased levels of soluble Aβ have been detected in aged canine brains, supporting the presence of a similar oligomer-driven pathogenic mechanism in CCD [5,209]. Across both species, overall plaque burden tends to correlate with disease stage, whereas soluble Aβ levels, synaptic loss, and impaired clearance mechanisms—including reduced glymphatic flow—show a closer association with the severity of cognitive decline [208,210].

### 4.3. APOE4, Alzheimer’s Disease, and Canine Cognitive Dysfunction: Molecular Parallels and Translational Implications

The apolipoprotein E (APOE) gene encodes a lipid transport protein critical for synaptic repair, cholesterol homeostasis, and neuronal resilience. In humans, the APOE ε4 allele (APOE-4) is the most important common genetic risk factor for late-onset AD: heterozygosity increases disease risk and lowers age at onset, whereas ε4 homozygosity further amplifies both risk and severity [211,212,213]. Mechanistically, the APOE ε4 allele is associated with enhanced amyloid-β aggregation and impaired clearance, exacerbation of tau pathology, disruption of lipid metabolism and synaptic homeostasis, and heightened neuroinflammatory responses, collectively driving progressive synaptic loss and neurodegeneration in Alzheimer’s disease [213,214,215]. In humans, carriers of the APOE ε4 allele frequently show accelerated rates of hippocampal atrophy and ventricular enlargement compared with non-carriers, even at preclinical or prodromal stages of the disease, thereby linking this genetic risk factor to a measurable neuroimaging phenotype [216,217].

All canine breeds examined shared an identical APOE amino acid sequence spanning residues 96–265, with arginine at positions 112 and 158, consistent with an isoform analogous to human APOE4 [218]. Phylogenetic analysis of APOE amino acid sequences revealed two clearly separated lineages, comprising distinct canine and human clusters [218].

Current data on dogs do not support the presence of a defined ε2/ε3/ε4 APOE polymorphism structure, and a direct canine orthologue of the human APOE4 risk allele has not yet been clearly established [4].

Nevertheless, APOE is expressed in the canine brain, and biological pathways central to APOE ε4-mediated risk in human Alzheimer’s disease—such as lipid metabolism, amyloid-β handling, neuroinflammatory responses, and synaptic integrity—also appear to play a critical role in the pathophysiology of CCD [5,8,219]. Accordingly, comparative and longitudinal studies of cognitively aging dogs are increasingly being used to elucidate how APOE-related mechanisms, together with other genetic and environmental factors, influence susceptibility to neurodegeneration. These investigations aim not only to improve the clinical management of CCD but also to inform preventive and therapeutic strategies relevant to human Alzheimer’s disease [185,219,220].

Molecular and phylogenetic data suggest that canine APOE4-like isoforms may confer an increased risk for neurological disorders and provide foundational genetic information to support future disease risk assessment and diagnostic approaches in dogs [218].

### 4.4. Presenilin

Presenilins (PSEN1, PSEN2) are highly conserved transmembrane proteins that constitute the catalytic core of the γ-secretase complex, mediating regulated intramembrane proteolysis of type I membrane proteins such as APP and Notch [221]. γ-secretase cleavage of APP generates Aβ peptides and AICD, while Notch processing releases NICD, a transcriptional regulator essential for cell fate decisions and neurogenesis; genetic studies show that presenilins are indispensable for Notch signaling and embryonic development [209]. Beyond their role in γ-secretase activity, presenilins also regulate key neuronal processes, including synaptic plasticity, endoplasmic reticulum Ca^2+^ homeostasis, and autophagy–lysosomal function [184]. Loss of presenilin function in neurons leads to impairments in long-term potentiation (LTP), memory deficits, and age-dependent neurodegeneration, underscoring their broader contribution to neuronal maintenance and resilience [222,223].

In humans, autosomal-dominant familial Alzheimer’s disease is strongly linked to mutations in PSEN1 and PSEN2 that reduce γ-secretase processivity, thereby increasing the relative production of longer, more aggregation-prone amyloid-β species such as Aβ_42_ and Aβ_43_, which promote plaque formation [224].

Clinically, these mutations are associated with an early disease onset and a particularly heavy cortical amyloid burden [225].

Canine PSEN1/PSEN2 are highly homologous to the human orthologs, and aged dogs naturally accumulate Aβ—particularly Aβ_42_—in cortical regions via analogous APP–γ-secretase processing, with plaque distribution and morphology closely paralleling human AD [5]. The extent of cortical Aβ deposition in dogs correlates with impairments in learning and memory [226].

Although presenilin gene mutations have not yet been systematically defined in canine cognitive dysfunction [185,206], the conservation of presenilin-dependent APP processing and amyloid pathology between dogs and humans supports aged dogs as a translational model of spontaneously occurring, amyloid-associated cognitive decline. Dogs exhibit β-amyloid (especially Aβ42) deposition and plaque formation via APP–γ-secretase pathways analogous to those in human AD [5,11], and the cortical Aβ burden correlates with impairments in learning and memory [5,226]. These neuropathological and cognitive parallels indicate that aged dogs recapitulate key aspects of human AD pathophysiology, despite the current lack of a clearly defined presenilin mutation-driven form of disease in this species [185,206].

### 4.5. Tau Biology and Isoforms

Tau is a microtubule-associated protein predominantly expressed in neurons, where it stabilizes microtubules and supports axonal transport [227,228]. It is generated from the MAPT gene through alternative mRNA splicing, producing isoforms that differ in both their N-terminal “projection” domain and C-terminal microtubule-binding repeats [229,230]. The N-terminal and C-terminal domains are linked by a proline-rich central region, giving tau a natively unfolded, highly soluble conformation under physiological conditions [231].

Tau–microtubule interactions are dynamically regulated through phosphorylation at multiple serine and threonine residues. At the N-terminus, alternative inclusion or exclusion of exons 2 and 3 generates tau isoforms containing zero, one, or two inserts (0N, 1N, or 2N), whereas at the C-terminus, splicing of exon 10 determines the presence of either four-repeat (4R) or three-repeat (3R) tau isoforms [232,233,234]. In the adult human brain, alternative splicing of the MAPT gene gives rise to six major tau isoforms—0N3R, 1N3R, 2N3R, 0N4R, 1N4R, and 2N4R—ranging from 352 to 441 amino acids in length. The relative abundance of 3R versus 4R tau is a critical determinant of tau fibril structure and, consequently, of disease phenotype [235].

In humans, specific combinations of tau isoforms and post-translational modifications give rise to distinct tau fibril “folds” that define a spectrum of primary tauopathies. Cryo-electron microscopy and biochemical analyses have identified at least eight canonical tau-misfolding diseases, including Alzheimer’s disease, primary age-related tauopathy (PART), chronic traumatic encephalopathy (CTE), Pick’s disease (PiD), corticobasal degeneration (CBD), argyrophilic grain disease (AGD), progressive supranuclear palsy (PSP), and globular glial tauopathy (GGT).

These entities differ in their predominant tau isoforms (mixed 3R + 4R in AD, PART, CTE; predominantly 3R in PiD; predominantly 4R in PSP, CBD, AGD, GGT), post-translational modification patterns, and disease-specific fibril conformations [235].

#### 4.5.1. Tau Isoforms in Pathology

Tau pathology in CCD remains less well characterized than in human tauopathies. Isoform-specific profiling of canine tau (3R vs. 4R balance and N-terminal insert usage) in CCD brains is limited, and no cryo-EM structures of canine tau filaments have yet been reported. Consequently, it is not known whether CCD harbors a single AD-like tau fibril fold or a spectrum of distinct folds analogous to the multiple structurally defined human tauopathies [235], as summarized in Table 2.

Available evidence indicates that aged dogs with CCD develop Alzheimer-like neuropathological changes, including cortical and hippocampal accumulation of hyperphosphorylated tau and, in some cases, tau aggregates, frequently in association with β-amyloid plaques [185,226,237]. In contrast, the widespread formation of classical neurofibrillary tangles (NFTs) that typifies advanced human Alzheimer’s disease is uncommon in dogs [14]. The prevailing interpretation is that, although tau lesions in CCD are less extensive, their coexistence with Aβ pathology and cognitive impairment supports the view that CCD lies within the same mechanistic spectrum as AD, with interspecies differences likely reflecting variations in lifespan, brain organization, and tau isoform expression [185,220]. Petersen et al. (2026) [238] employed a plasma p-tau217 assay to predict the future onset of Alzheimer’s disease in cognitively unimpaired individuals. Their predictive model estimates the age at which an individual first develops biological markers of Alzheimer’s pathology and forecasts the subsequent emergence of clinical symptoms, with a mean prediction error of approximately three to four years. Samelson et al. (2026) [239], through genome-wide screening of human stem cell-derived neurons, identified key regulators of toxic tau accumulation, implicating the CRL5–SOCS4 complex in tau homeostasis. This complex appears to facilitate tau protein clearance and may be associated with resilience in patients with Alzheimer’s disease. The proposed mechanism suggests that mitochondrial dysfunction initiates tau fragmentation into pathogenic species that contribute to disease progression. These findings highlight a critical pathway in tau aggregation and identify a promising target for therapeutic intervention.

#### 4.5.2. Tau Misfolding, Aggregation, and Thermodynamic Perspective

##### Hyperphosphorylation and Post-Translational Modification

Hyperphosphorylation of tau is a key early event in its pathological transformation. In AD and CCD-like conditions, tau becomes abnormally hyperphosphorylated at multiple serine/threonine residues (e.g., Ser202/Thr205, Ser396/404), primarily through kinases such as GSK-3β, CDK5, and MAPKs, which are upregulated by chronic metabolic stress, inflammation, and Aβ exposure [240,241]. This reduces tau’s affinity for microtubules, causing detachment, mislocalization from axons to somatodendritic compartments, and expansion of the cytosolic, aggregation-prone tau pool [231,240,242].

Additional post-translational modifications—including truncation, acetylation, ubiquitination, and glycation—further destabilize tau’s native conformation, promote β-sheet formation, and expose aggregation-prone hexapeptide motifs (e.g., PHF6* [VQIINK] and PHF6 [VQIVYK]), enabling tau monomers to assemble into soluble oligomers and ultimately into paired helical filaments (PHFs) and straight filaments. Biophysical and structural studies show that the PHF6 hexapeptide (VQIVYK) forms exceptionally stable steric-zipper β-sheet fibrils and is a dominant driver of tau aggregation, whereas PHF6* (VQIINK), although aggregation-prone, appears less capable of forming such stable fibrillar cores [243,244].

The accumulation of these filaments as NFTs, neuropil threads, and dystrophic neurites is a hallmark of AD and related tauopathies [231,245,246].

##### Thermodynamic Hypothesis of Tau Aggregation

Tau folding and misfolding in humans and dogs can be interpreted within the thermodynamic hypothesis of protein aggregation, which posits that many proteins are expressed near their supersaturation limits, such that native conformations are only metastable relative to more stable amyloid states [247,248,249]. In human tauopathies such as AD, PSP, and CBD, cryo-EM has revealed β-sheet-rich tau fibril folds that correspond to low-free-energy states increasingly populated as aging, mutations, and post-translational modifications erode proteostasis and destabilize soluble tau [235,245,250].

Aged dogs with CCD develop Alzheimer-like cortical and hippocampal lesions with hyperphosphorylated and aggregated tau, indicating that canine tau can likewise cross the free-energy barrier toward aggregation-prone conformations when supersaturation overwhelms neuronal quality control systems [185]. Although canine tau fibrils have not yet been structurally subclassified as in human tauopathies, the shared features of age-dependent tau accumulation, amyloid co-pathology, and a proteome “living on the edge of solubility” support common thermodynamic principles of tau misfolding across species [249,250].

##### Prion-like Propagation of Tau in AD and CCD

A growing body of evidence supports a prion-like mechanism of tau spread in AD and CCD-related tauopathies. Misfolded tau assemblies can be released from affected neurons via exocytosis, ectosomes, exosomes, or passive leakage and are subsequently taken up by neighboring cells through endocytosis, macropinocytosis, or receptor-mediated mechanisms [251]. Once internalized, these seeds induce templated misfolding of endogenous soluble tau, amplifying pathology across anatomically connected networks [251,252].

This propagative behavior mirrors the stereotyped progression of tau pathology described by Braak staging, in which early involvement of transentorhinal and hippocampal regions is followed by spreading into association cortices and, ultimately, primary sensory–motor areas [246]. In the setting of chronic vascular insufficiency, blood–brain barrier (BBB) disruption and low-grade neuroinflammation may further facilitate the extracellular persistence and interstitial transport of tau seeds, thereby enhancing their regional dissemination. Each step is summarized in Table 3.

#### 4.5.3. Interplay Between Tau Pathology, Parenchymal Amyloid Plaques, and Cerebral Amyloid Angiopathy

In AD, tau pathology coexists and interacts closely with Aβ amyloid pathology. Aβ is generated from APP by sequential β- and γ-secretase cleavage, producing Aβ40 and Aβ42 peptides that misfold and aggregate into oligomers and fibrils, ultimately forming neuritic plaques in the brain parenchyma [204]. Genetic, experimental, and biomarker data support a model in which Aβ aggregation is an upstream event that accelerates or amplifies tau pathology, rather than tau misfolding occurring in isolation [256,257].

Aβ oligomers and plaques promote tau hyperphosphorylation and mislocalization by activating kinases such as GSK-3β, CDK5, and MAPKs, and inhibiting phosphatases such as PP2A [241]. Aβ also induces synaptic dysfunction, intracellular Ca^2+^ dysregulation, oxidative stress, and microglia- and astrocyte-mediated inflammation, further favoring tau misfolding and aggregation [242,257]. In transgenic mouse models, expression of human APP/Aβ enhances tau pathology and neurodegeneration, whereas tau reduction mitigates Aβ-induced toxicity, indicating that tau is a key downstream effector of Aβ [258].

Neuritic plaques are typically composed of a dense amyloid-β (Aβ) core surrounded by dystrophic neurites enriched in hyperphosphorylated and aggregated tau, underscoring a focal convergence of amyloid and tau pathology within affected brain regions [259]. Positron emission tomography (PET) studies in humans indicate that cortical Aβ deposition generally precedes the widespread accumulation of tau within the neocortex; however, once tau pathology extends beyond mesial temporal structures, its burden shows a stronger association than Aβ with regional brain atrophy and cognitive decline [256,260].

Thus, across AD and CCD-like dementias, Aβ acts predominantly as an initiator and amplifier of tau misfolding, whereas misfolded tau is a principal effector of synaptic dysfunction and neuronal loss.

### 4.6. Cerebral Amyloid Angiopathy (CAA)

Aβ deposition, predominantly Aβ40, in leptomeningeal and cortical vessel walls is frequent in AD and is also observed in aged dogs with CCD [5,261]. CAA arises from impaired perivascular and glymphatic clearance of Aβ along intramural periarterial drainage pathways, processes that decline with age, vascular disease, and structural vessel changes [13,262]. Vascular Aβ deposition leads to vessel wall thickening, smooth-muscle cell loss, microaneurysm formation, and increased susceptibility to microbleeds and lobar intracerebral hemorrhage [261]. These vascular alterations lead to chronic cerebral hypoperfusion, focal ischemic injury, and disruption of the blood–brain barrier, thereby creating an environment characterized by oxidative stress and sustained inflammatory activation that can indirectly promote tau hyperphosphorylation and aggregation within the surrounding brain parenchyma [241,263,264]. Neuropathological studies further indicate that severe cerebral amyloid angiopathy (CAA), particularly capillary CAA (type 1), frequently co-occurs with substantial cortical tau pathology in Alzheimer’s disease and mixed dementias [265]. In hereditary forms of CAA, such as those associated with Dutch-type APP mutations, pronounced vascular Aβ deposition is accompanied by cortical tau lesions and progressive cognitive decline, even in the presence of relatively modest parenchymal plaque burden. These observations suggest that vascular amyloidosis alone is sufficient to establish a tau-permissive environment that contributes to neurodegenerative progression [263,266].

Impaired perivascular and glymphatic clearance in CAA likely hinders removal of extracellular tau species, including oligomers and seeds, from the interstitial fluid [13,258]. This compromised clearance may extend the half-life of pathogenic tau conformers and facilitate their spread along dysfunctional perivascular routes in both CCD and human vascular-degenerative dementias.

Collectively, tau misfolding and aggregation in AD, CCD, and related disorders occur within a broader landscape of proteopathic and vascular pathology. Aβ aggregation in the brain parenchyma forms neuritic plaques that colocalize with tau-positive dystrophic neurites, while vascular Aβ deposition in CAA perturbs neurovascular coupling, BBB integrity, and clearance pathways, all of which intensify tau pathology [259,261,262]. For CCD and human AD spectrum dementias, this integrated view implies that effective therapies will likely need to target both misfolded tau and Aβ and to restore vascular health and clearance mechanisms—by reducing tau phosphorylation and aggregation, lowering Aβ production or aggregation, and enhancing glymphatic/perivascular clearance and neurovascular unit stability.

### 4.7. Neuronal and Synaptic Loss

Neuronal and synaptic degeneration represents the structural basis of cognitive decline in both conditions. In Alzheimer’s disease, the pronounced loss of pyramidal neurons in the hippocampal CA1 region, subiculum, and association cortices is well documented [267], and reductions in synaptic density—rather than amyloid plaque burden—constitute the strongest histopathological correlate of dementia severity [210,268]. Aged dogs with CCD display comparable patterns of neurodegeneration, including neuronal loss and synaptic alterations, particularly within the frontal cortex and hippocampus, supporting a shared substrate for cognitive impairment across species [205].

### 4.8. Neurotransmitter System Dysfunction

The cholinergic hypothesis of AD posits that degeneration of basal forebrain cholinergic neurons and consequent cortical cholinergic deficiency contribute critically to attentional and mnemonic deficits [269,270]. Post-mortem studies confirm decreased choline acetyltransferase (ChAT) activity and reduced acetylcholine (ACh) release in the AD cortex [271].

Comparable cholinergic dysfunction has been described in CCD. Aged dogs show reduced ChAT activity and degeneration of cholinergic neurons in the basal forebrain and hippocampus [272], which clinically manifests as impaired learning, disorientation, and altered sleep–wake cycles [206]. Additionally, alterations in dopaminergic and noradrenergic systems have been reported in both diseases, potentially contributing to affective and motivational disturbances [272,273].

### 4.9. Mitochondrial Dysfunction and Oxidative Stress

Mitochondrial dysfunction and oxidative stress are well-established hallmarks of Alzheimer’s disease pathology [274,275]. Both amyloid-β and tau disrupt mitochondrial dynamics, impair respiratory chain efficiency, increase the generation of reactive oxygen species (ROS), and compromise intracellular calcium buffering. The resulting oxidative damage to lipids, proteins, and nucleic acids further undermines synaptic integrity and neuronal viability, thereby contributing to progressive neurodegeneration [276,277].

In aged canine brains, elevated markers of oxidative injury—such as protein carbonyls, 4-hydroxynonenal (4-HNE), and 8-hydroxy-2′-deoxyguanosine (8-OHdG)—closely mirror those reported in human Alzheimer’s disease [8]. Importantly, dietary supplementation with antioxidants in aged dogs has been shown to improve specific cognitive functions and reduce oxidative biomarkers, reinforcing the pathogenic role of oxidative stress in CCD [157,179]. These interventional findings in dogs provide experimental support for mechanisms that, in humans, are largely inferred from observational studies.

### 4.10. Neuroinflammation and Glial Activation

Microglial and astrocytic responses to Aβ, tau, and damaged neurons are initially protective but become maladaptive when chronically activated [278]. In AD, microglial activation is associated with upregulation of pro-inflammatory cytokines (IL-1β, TNF-α, IL-6), complement factors, ROS, and nitric oxide, all of which can impair synaptic plasticity and promote further neurodegeneration [278,279,280].

Homologous phenomena occur in CCD. Aged dogs with a high Aβ burden show increased microglial and astrocytic reactivity in the cortex and hippocampus [2,5]. Elevated inflammatory markers correlate with cognitive impairment, suggesting that neuroinflammation in CCD is not merely an epiphenomenon but a driver of disease progression [185]. Chronic inflammation can also alter astrocyte polarity and aquaporin-4 (AQP4) distribution, thereby impairing glymphatic function and further reducing clearance of Aβ and tau [13,23].

### 4.11. Glymphatic System

The brain parenchyma lacks a conventional lymphatic network and instead depends on the glymphatic system, a perivascular clearance pathway in which cerebrospinal fluid enters the brain from the subarachnoid space along penetrating arteries, exchanges with interstitial fluid, and is subsequently cleared along paravenous routes toward meningeal lymphatic vessels and cervical lymph nodes [13,281]. This convective transport mechanism facilitates the removal of extracellular solutes, including amyloid-β (Aβ), tau, and α-synuclein, particularly from deep parenchymal regions that are relatively distant from the blood–brain barrier [262]. From a pathogenetic perspective, efficient glymphatic transport critically depends on aquaporin-4 water channels, which are highly enriched and radially polarized at astrocytic endfeet surrounding perivascular spaces [18,54]. Experimental ablation of Aqp4 or loss of perivascular AQP4 localization markedly reduces CSF influx and impairs the clearance of Aβ and tau, resulting in increased parenchymal deposition of these proteins [13,21]. Aging, vascular risk factors, sleep disruption, and dysfunction of meningeal lymphatic drainage converge to reduce glymphatic efficiency [282], thereby providing a mechanistic link between these factors and the accumulation of amyloid pathology in the brain [23,39,283].

In Alzheimer’s disease, perivascular AQP4 polarization is disrupted and AQP4 redistributes to non-end-foot astrocytic membranes [23]. Post-mortem human studies by Zeppenfeld and colleagues link preserved perivascular AQP4 to lower Aβ/tau burden and better cognition, whereas AQP4 depolarization associates with more severe pathology [48,284]. Valenza and others therefore propose a feed-forward loop, in which initial protein aggregation perturbs AQP4 anchoring, further impairs perivascular clearance, and accelerates aggregation [285]. Experimental modulation of AQP4—for example, with 5-caffeoylquinic acid—partially restores perivascular localization, enhances Aβ efflux, and improves cognition in AD mouse models, directly implicating the AQP4–glymphatic axis in disease pathogenesis and suggesting a tractable therapeutic target [41,286].

Keil and Buccellato et al. extend this framework beyond Alzheimer’s disease. In transgenic rodent models, Aqp4 deletion or mislocalization, as well as aging, hypertension, diabetes, cerebrovascular disease, traumatic brain injury, and chronic sleep disruption, consistently reduce glymphatic transport and are associated with increased accumulation of Aβ/tau or α-synuclein pathology [15,287]. In models of Parkinson’s disease (PD), reduced AQP4 expression exacerbates α-synuclein deposition, while ablation of meningeal lymphatic vessels worsens synucleinopathy, suggesting that impairment of both parenchymal and meningeal clearance pathways facilitates proteopathic seeding and spread [288]. Human genetic studies further indicate that AQP4 polymorphisms influence cerebral amyloid burden, sleep–Aβ interactions, and the rate of cognitive decline in AD and PD, supporting the concept that inter-individual differences in glymphatic efficiency may modify disease progression [289,290,291,292].

Neuroimaging and fluid biomarker studies provide converging, though largely correlative, evidence for this pathogenic model. Intrathecal gadolinium-enhanced MRI enables direct visualization of perivascular CSF–interstitial fluid exchange, demonstrates increased clearance during sleep, and reveals age-related slowing, findings consistent with an age-associated decline in glymphatic function [14,293,294]. Indirect imaging markers, including enlarged perivascular spaces, diffusion tensor imaging along the perivascular space (DTI-ALPS), and arterial spin-labeling-based water exchange measures, also suggest altered perivascular fluid dynamics in AD and cerebral small-vessel disease, although their quantitative relationship to solute clearance remains incompletely validated [17,295,296,297].

Taya et al. [59] provide longitudinal clinical evidence linking glymphatic dysfunction to disease progression. In participants from the Alzheimer’s Disease Neuroimaging Initiative, a lower ALPS index—interpreted as reduced glymphatic activity—was associated with faster Aβ PET accumulation, accelerated cortical atrophy in AD-vulnerable regions, increased risk of conversion from Aβ-negative to Aβ-positive biomarker status, and a higher likelihood of progression from cognitive normality or mild cognitive impairment to AD dementia. Similar associations were observed in the UK Biobank cohort [44,298]. Mediation analyses indicated that the relationship between ALPS and cognitive decline was fully mediated by increased amyloid burden and brain atrophy, positioning glymphatic dysfunction upstream of established AD biomarkers within a proposed pathogenic cascade [44,299].

Integrating experimental, imaging, and clinical findings, it was proposed glymphatic failure—driven by AQP4 depolarization, vascular dysfunction, sleep and circadian disturbances, and impairment of meningeal lymphatic drainage—as a shared pathogenic mechanism linking idiopathic normal pressure hydrocephalus, AD, PD, and progressive supranuclear palsy [43,293,300,301,302,303,304]. They further discuss experimental interventions aimed at restoring glymphatic function, including focused ultrasound, hypertonic saline, mannitol, dexmedetomidine, and AQP4-targeted approaches, as potential strategies to address upstream clearance deficits and downstream proteopathic neurodegeneration [300,301,302,303].

Taken together, studies by Silva, Keil, Buccellato, Huang, and Ohara [43,44,45,47,304] support a unified pathogenic model in which AQP4-dependent glymphatic transport represents a modifiable interface between vascular integrity, immune regulation, sleep physiology, and the accumulation of Aβ, tau, and α-synuclein. Establishing causality in humans will require the development of standardized, non-invasive measures of glymphatic function and interventional trials to determine whether enhancement of perivascular clearance can meaningfully alter biomarker trajectories and clinical outcomes.

## 5. Neuropathology of Alzheimer’s Disease

### 5.1. Gross Pathology

**Figure 4:**
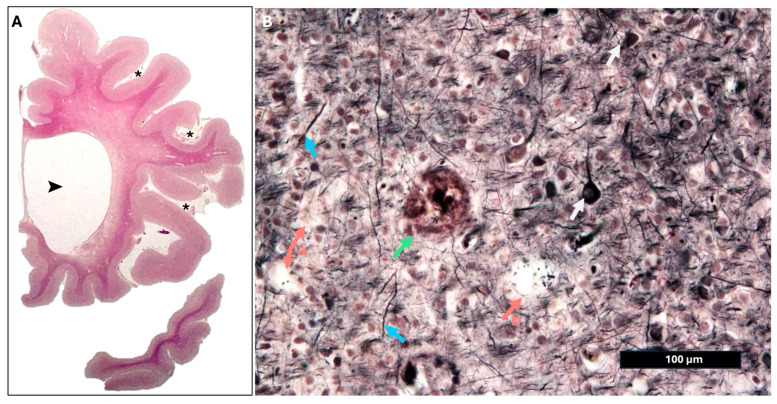
Alzheimer’s disease. (**A**) Coronal section of the human cerebral cortex—right hemisphere at the mesencephalic level—showing widening of the cortical sulci secondary to diffuse atrophy of the cerebral gyri (*), with dilation of the right lateral ventricle (➤). Hematoxylin and eosin staining of embedding dehydrated tissue in nitrocellulose (celloidin) 15 micron; magnification 1×. (**B**) Histological microphotograph of the motor cerebral cortex showing neuropil threads (light blue arrow), a neuritic plaque (green arrow), neurofibrillary tangles (white arrow), neuropil rarefaction (coral red—a), and vacuolization (coral red—b). Bielschowsky silver stain; magnification 20×; scale bar = 100 μm. Courtesy of Prof. Giorgio Pilleri (1985). From Hirnanatomisches des Menschen, Waldau, Bern, Switzerland.

In advanced AD, the brain shows diffuse cortical atrophy, most pronounced in the medial temporal lobes (hippocampus, entorhinal cortex), association neocortex, and posterior cingulate cortex, with relative sparing of primary motor and sensory cortices in earlier stages [9,246]. Sulci are widened and gyri thinned, especially in frontal, temporal, and parietal regions; overall brain weight is reduced (Figure 4). The hippocampus and amygdala are notably shrunken, and in coronal sections of the temporal lobe, atrophy of the hippocampal formation (CA1, subiculum) and entorhinal cortex is conspicuous [305]. The ventricles are enlarged secondary to ex vacuo hydrocephalus (Figure 4). In late stages, atrophy becomes more generalized, involving deep gray matter structures such as the diencephalon and basal forebrain, particularly the nucleus basalis of Meynert, correlating with cholinergic deficits [306]. Grossly visible focal lesions are not typical, but cerebral amyloid angiopathy (CAA) with lobar microbleeds or macrohemorrhages can occur and is more prevalent in APOE ε4 carriers [307,308].

### 5.2. Histopathology

The defining histologic lesions of AD are extracellular amyloid-β (Aβ) plaques and intraneuronal neurofibrillary tangles (NFTs) (Figure 3). Additional microscopic abnormalities are observed in neurons, synapses, glia, and the cerebrovascular wall.

Amyloid plaques

(a) Plaques are composed predominantly of Aβ peptides (Aβ_40_/Aβ_42_) derived from the amyloid precursor protein (APP) via β- and γ-secretase cleavage.
(b) Neuritic (senile) plaques, the diagnostic hallmark of AD, consist of a dense fibrillar Aβ core surrounded by dystrophic neurites, activated microglia and reactive astrocytes [9,259].
(c) Diffuse plaques, often Aβ_42_-rich, appear earlier in disease and may lack neuritic elements and dense fibrils.
(d) Plaque deposition is initially prominent in the neocortex, then involves the hippocampus and limbic structures and finally subcortical regions as pathology advances [259].

### 5.3. Neurofibrillary Tangles and Tau Pathology

NFTs are composed of paired helical filaments formed by abnormally hyperphosphorylated tau, predominantly comprising mixed 3R and 4R tau isoforms, which accumulate within neuronal cell bodies and proximal dendrites [246,309]. NFTs are accompanied by neuropil threads and tau-positive dystrophic neurites, reflecting the spread of tau pathology into axonal and dendritic compartments. The regional distribution of tau pathology follows the characteristic Braak staging pattern, progressing from the transentorhinal and entorhinal cortex (stages I–II) to the hippocampus and limbic regions (stages III–IV), and ultimately involving widespread neocortical areas in advanced disease (stages V–VI) [246]. Importantly, the burden and distribution of tau pathology show a stronger correlation with cognitive impairment than amyloid plaque load, underscoring the central role of tau-mediated neurodegeneration in clinical disease expression [310].

### 5.4. Neuronal and Synaptic Pathology

Neuronal degeneration is a prominent feature of Alzheimer’s disease and is particularly evident in pyramidal neurons of the hippocampal CA1 region, the entorhinal cortex, association cortices, and subcortical cholinergic nuclei, including the nucleus basalis of Meynert [306]. In parallel, synaptic loss—assessed either ultrastructurally or through synaptic protein markers—represents one of the most robust pathological correlates of dementia severity [210,268]. A loss of synaptic connectivity precedes overt neuronal death in many regions and is closely associated with impairments in learning, memory, and executive function, highlighting synaptic dysfunction as a key substrate of cognitive decline.

### 5.5. Glial and Vascular Changes

Glial and vascular alterations are integral components of Alzheimer’s disease pathology. Reactive astrogliosis, characterized by increased GFAP expression, and microgliosis, marked by activation of Iba1- or CD68-positive microglia, are commonly observed surrounding amyloid plaques and neurofibrillary tangles, reflecting a state of chronic neuroinflammation [9,278]. Vascular pathology is also frequent, most notably in the form of cerebral amyloid angiopathy (CAA), in which β-amyloid—predominantly Aβ40—accumulates within leptomeningeal and cortical vessel walls, increasing susceptibility to microhemorrhages and cortical superficial siderosis [307,311]. In addition, mild white matter degeneration and myelin loss can be detected, particularly in later disease stages, further contributing to network disconnection and cognitive impairment.

### 5.6. Immunohistochemistry in Alzheimer’s Disease

Immunohistochemistry plays a central role in the definitive classification and neuropathological staging of Alzheimer’s disease. Immunostaining for β-amyloid using antibodies such as 4G8, 6E10, anti-Aβ42, and anti-Aβ40 robustly labels both diffuse and cored amyloid plaques, as well as vascular amyloid deposits, thereby enabling semiquantitative assessment of parenchymal plaque burden and cerebral amyloid angiopathy (CAA) [259,305]. Phosphorylated tau is detected using antibodies such as AT8, PHF-1, and CP13, which label neurofibrillary tangles, neuropil threads, and tau-positive dystrophic neurites and are routinely employed to assign Braak neurofibrillary stages [242,283]. Neuronal markers, including NeuN and MAP2, are used to highlight neuronal loss and dendritic pathology, while synaptic markers such as synaptophysin and PSD-95 reveal reductions in synaptic density. Glial activation is assessed using astrocytic markers such as GFAP and microglial markers including Iba1 or CD68, which demonstrate plaque-associated and regionally increased gliosis. In addition, vascular markers such as collagen IV and smooth muscle actin, when combined with Aβ immunostaining, allow for the visualization of CAA and associated vessel wall alterations, providing insight into the vascular component of Alzheimer’s disease pathology.

### 5.7. National Institute on Aging–Alzheimer’s Association (NIA-AA) Guidelines and ABC Scoring System

In current practice, Alzheimer’s disease pathology is integrated using the NIA–AA “ABC” criteria, which combine three complementary semiquantitative assessment systems: the Thal phase of amyloid-β (Aβ) plaque deposition (A; stages I–V), the Braak neurofibrillary tangle (NFT) stage (B; stages 0–VI), and the CERAD neuritic plaque score (C; scores 0–3) [305,312]. Together, these frameworks provide a standardized approach for classifying the extent and distribution of amyloid and tau pathology in the human brain. By contrast, no formal analog of this integrated “ABC” scoring system has yet been established for canine cognitive dysfunction.

The Thal staging system describes the spatial progression of Aβ deposition in AD. In the earliest phase (phase I), Aβ plaques are largely confined to the neocortex. With advancing disease, deposition extends sequentially to the hippocampal and entorhinal regions (phase II), the basal ganglia and diencephalon (phase III), brainstem structures (phase IV), and ultimately the cerebellum (phase V). This Aβ-centric framework complements, but is conceptually distinct from, Braak staging, which focuses on tau pathology.

Braak NFT staging characterizes the topographical spread of neurofibrillary tangles across the brain. In stages I–II, tangles are predominantly restricted to the transentorhinal cortex. During intermediate stages (III–IV), tau pathology extends into limbic regions, including the hippocampus, while in advanced stages (V–VI) it involves widespread association neocortical areas. This tau-based staging scheme reflects the stereotyped propagation of neurofibrillary pathology and should be distinguished from the severity and distribution of senile Aβ plaques, which follow a different spatial trajectory.

In addition, the CERAD neuritic plaque score provides a semiquantitative measure of cortical neuritic plaque density in Alzheimer’s disease. Based on standardized microscopic assessment of selected neocortical regions, plaque burden is classified as none, sparse, moderate, or frequent (scores 0–3), thereby integrating both amyloid deposition and associated neuritic pathology into a single grading system.

## 6. Neuropathology of Canine Cognitive Dysfunction

### 6.1. Gross Pathology

Dogs affected by canine cognitive dysfunction typically exhibit generalized cortical atrophy accompanied by ventricular enlargement and widening of cortical sulci, changes that resemble those observed in human Alzheimer’s disease, although they are usually less severe in magnitude [6,185]. Cortical atrophy is most pronounced in the frontal and temporal lobes and, in some cases, also involves the parietal cortex, and is associated with a reduction in overall brain weight compared with age-matched control dogs [10]. Magnetic resonance imaging and postmortem investigations consistently demonstrate hippocampal volume loss, which correlates with cognitive impairment, particularly deficits in spatial learning and memory [10,313]. The cerebellum is relatively spared. Gross hemorrhagic lesions are uncommon; however, age-related vascular alterations and cerebral microbleeds may be present.

### 6.2. Histopathology

#### 6.2.1. Amyloid-β Pathology

The most consistent histopathological feature of CCD is amyloid-β (Aβ) deposition, which occurs predominantly within the cerebral cortex—especially in frontal, temporal, and parietal association areas—and, to a lesser extent, in the hippocampus [5,220]. Diffuse Aβ plaques are frequently observed and may be present even in cognitively normal aged dogs, whereas cored or neuritic plaques tend to be more prevalent or abundant in dogs with clinically documented CCD [5,6]. The deposited peptide is primarily Aβ42, resembling early-stage human amyloid plaques, although Aβ40-containing deposits are also detected within cerebral vessel walls [220]. Across both longitudinal and cross-sectional studies, cortical Aβ burden correlates with measures of cognitive decline, particularly impairments in learning and memory [5,10].

#### 6.2.2. Tau and Other Proteinopathies

Classical neurofibrillary tangles composed of hyperphosphorylated tau, which are a defining feature of advanced human Alzheimer’s disease, are infrequent or absent in most studies of canine cognitive dysfunction [185]. Although scattered phospho-tau-positive neurons or neuropil threads have been reported in some cases, these findings do not exhibit the extensive, laminar, Braak-like progression characteristic of human AD [185]. Taken together, the available evidence supports the view that CCD represents a predominantly amyloid-β-centric pathology, with relatively limited tau involvement compared with human Alzheimer’s disease.

#### 6.2.3. Neuronal and Synaptic Changes

Neuronal and synaptic alterations are consistently reported in canine cognitive dysfunction, although their severity can be variable. Neuronal loss and shrinkage have been described in the frontal and temporal cortices as well as in the hippocampus; however, distinguishing these changes from those associated with normal aging can be challenging in some cases [185]. Synaptic loss, assessed using synaptophysin or other synaptic markers, appears to parallel cognitive decline and is particularly evident in the hippocampus and frontal cortex, supporting a close relationship between synaptic integrity and cognitive performance in CCD [10,185]. In addition, degeneration of white matter tracts and demyelination—especially within the frontal lobes—have been documented in older dogs and may further contribute to cognitive impairment by disrupting cortical connectivity [205].

#### 6.2.4. Glial and Vascular Changes

Glial and vascular pathology represents an important component of CCD. Both astrogliosis and microgliosis are commonly observed and are frequently associated with amyloid-β plaques, reflecting a neuroinflammatory response that closely resembles that seen in Alzheimer’s disease [220]. Cerebral amyloid angiopathy, characterized by Aβ deposition within cerebral and leptomeningeal vessel walls, is a common finding in aged dogs, regardless of overt cognitive impairment, but tends to be more pronounced in animals with clinically evident CCD [7]. In addition, age-related microinfarcts and small-vessel disease may coexist with amyloid pathology, although these vascular lesions have not yet been characterized as systematically in dogs as they have been in human mixed dementias.

#### 6.2.5. Immunohistochemistry in Canine Cognitive Dysfunction

Immunohistochemical approaches are widely used to characterize neuropathological changes in CCD. Antibodies raised against human amyloid-β, including 4G8, 6E10, and anti-Aβ42 or anti-Aβ40, cross-react with canine Aβ and robustly label both parenchymal plaques and vascular amyloid deposits, allowing for quantitative assessment of plaque burden and regional distribution across cortical and hippocampal areas [5,220]. In contrast, phospho-tau immunostaining using antibodies such as AT8 typically reveals only limited or focal immunoreactivity and rarely demonstrates fully developed neurofibrillary tangles, underscoring the relative paucity of classic tauopathy in CCD compared with human Alzheimer’s disease [185]. Glial markers, including GFAP for astrocytes and Iba1 or CD68 for microglia, demonstrate plaque-associated and regionally increased gliosis, enabling direct comparison of neuroinflammatory responses between CCD and AD [314]. Neuronal and synaptic markers such as NeuN, MAP2, and synaptophysin further document neuronal and synaptic loss in relation to amyloid burden and clinical cognitive scores [10]. Vascular amyloid deposition can be visualized using double immunolabeling for Aβ and vascular markers such as collagen IV, confirming the presence of cerebral amyloid angiopathy and associated vasculopathy [7].

AD and CCD neuropathological profiles are summarized in Table 4.

## 7. Pathological Diversity and Similarity Between Alzheimer’s Disease and Canine Cognitive Dysfunction

AD and CCD exhibit both convergent and divergent neuropathological features. Both disorders show progressive cortical and hippocampal atrophy, ventricular enlargement, and Aβ deposition in association cortices and the hippocampus, accompanied by CAA and glial activation [5,220,259]. In both species, Aβ plaques can appear in cognitively normal aged individuals, but higher plaque burden and more pronounced neuritic features tend to correlate with more severe cognitive dysfunction [5,9,10]. Immunohistochemically, human and canine Aβ are sufficiently conserved that similar antibody label plaques and vascular deposits, and plaque-associated astrocytosis and microgliosis can be demonstrated with comparable glial markers [220] (Table 2).

The most pronounced divergence between Alzheimer’s disease and canine cognitive dysfunction lies in tau pathology and overall lesion composition. Human AD is defined by abundant accumulation of hyperphosphorylated tau in the form of neurofibrillary tangles, neuropil threads, and a highly stereotyped Braak staging pattern that closely parallels the progression of cognitive decline [246]. By contrast, CCD typically exhibits minimal or absent classical NFTs on histological and immunohistochemical examination and lacks a clearly defined Braak-like pattern of tau propagation [185]. Accordingly, whereas AD represents a combined amyloid-β and tau proteinopathy, CCD is best characterized as a predominantly Aβ-dominant encephalopathy with limited or inconsistent tau involvement. This fundamental difference likely contributes to the generally milder or more heterogeneous patterns of neuronal and synaptic loss observed in CCD compared with advanced stages of human AD, as well as to differences in clinical course and progression. Although vascular pathology—particularly cerebral amyloid angiopathy—is a shared feature in both species, the spectrum, severity, and clinical consequences of mixed vascular–degenerative lesions are far more comprehensively characterized in human AD than in CCD [308]. Overall, CCD can be regarded as a partial, Aβ-centered analog of AD, recapitulating key amyloid-related and neuroinflammatory features while lacking the full tau-driven neurodegenerative cascade that typifies human AD.

Neuropathological similarities and differences between Alzheimer’s disease and canine cognitive dysfunction are summarized in Table 5. This comparison highlights shared features, including cortical and hippocampal atrophy, parenchymal Aβ deposition, cerebral amyloid angiopathy, and glial activation, alongside species-specific differences in lesion burden, distribution, and molecular composition. Particular emphasis is placed on the markedly more extensive and stereotyped tau pathology characteristic of human AD, in contrast to the predominantly Aβ-centric and sparsely tau-positive phenotype observed in CCD.

By integrating structural, molecular, and inflammatory parameters, Table 4 delineates how CCD recapitulates key aspects of the AD spectrum while lacking the full tau-driven neurodegenerative cascade.

This comparative framework underpins the use of CCD as a partial but informative translational model for human Alzheimer-type pathology.

## 8. Translational Impact Related to Alzheimer’s Disease and Canine Cognitive Dysfunction Through Transmission Electron Microscopy

### 8.1. Alzheimer’s Disease, Canine Cognitive Dysfunction and Transmission Electron Microscopy

At the ultrastructural level, brains affected by AD exhibit dystrophic neurites, synaptic loss, mitochondrial abnormalities, and characteristic Aβ fibrils and paired helical filaments. These alterations can be visualized and quantified by transmission electron microscopy, which has been essential for delineating the morphology of amyloid and tau aggregates [210,245].

In aged dogs and in canine cognitive dysfunction, TEM has demonstrated ultrastructural alterations analogous to those observed in human AD, including synaptic degeneration, swollen and dystrophic neurites, mitochondrial alterations, and Aβ fibrils within parenchymal plaques and vascular walls [6,220]. These observations support the use of aged dogs as a model of early-stage AD and permit direct cross-species comparisons at the nanoscale.

Because aged dogs develop spontaneous Aβ pathology and cognitive decline under naturalistic conditions, they constitute an intermediate translational model between rodents and humans. TEM-based ultrastructural analyses, in conjunction with behavioral assessments and neuroimaging, can be employed as follows:

(a) To validate candidate biomarkers across species;
(b) To evaluate disease-modifying interventions at synaptic and fibrillar scales;
(c) To define the temporal relationship between Aβ accumulation, synaptic alterations, and cognitive impairment.

The integration of TEM findings with molecular, imaging, and behavioral data in both dogs and humans is likely to elucidate conserved mechanisms of neurodegeneration and to facilitate the development of more predictive translational approaches for Alzheimer’s disease.

#### 8.1.1. Amyloid Fibrils and Plaques

TEM established the fibrillar nature of amyloid deposits in AD and enabled precise measurement of fibril diameter, periodicity, and packing [210]. Subsequent developments in TEM and cryo-EM have resolved near-atomic structures of tau filaments and Aβ fibrils [245].

#### 8.1.2. Neurofibrillary Tangles

Paired helical and straight tau filaments can be directly visualized and morphometrically analyzed using TEM, allowing for the correlation of filament architecture with biochemical composition and clinical phenotype [245].

#### 8.1.3. Synapses and Organelles

TEM permits quantification of synaptic density, synaptic vesicle pools, mitochondrial morphology, and autophagic structures, thereby linking cognitive impairment to synaptic and subcellular pathology in AD and CCD [6,210].

#### 8.1.4. Myelin and Axons

Age-associated axonal degeneration, myelin splitting, and spheroid formation can be systematically evaluated by TEM in both humans and dogs, providing a structural basis for white matter alterations associated with cognitive dysfunction [10].

The translational impact of transmission electron microscopy-derived observations in Alzheimer’s disease and canine cognitive dysfunction is summarized in Table 6.

TEM enables ultrastructural characterization of amyloid fibrils, tau filaments, synapses, and organelles, allowing for direct comparison of lesion morphology between humans and dogs.

By correlating these nanoscale alterations with cognitive profiles and imaging biomarkers, TEM helps validate CCD as a spontaneous, clinically relevant model of early-stage AD.

Ultrastructural readouts also provide sensitive endpoints for preclinical trials, permitting assessment of how candidate therapeutics modify synaptic integrity and aggregate burden.

Together, these TEM-based insights support more reliable cross-species extrapolation of mechanistic findings and therapeutic responses in AD research.

## 9. Neuroclinical Aspects

Canine Cognitive Dysfunction is a progressive, age-related neurodegenerative disorder in dogs, characterized by a diverse array of behavioral abnormalities that reflect widespread cerebral involvement. There is no clear correlation between breed and predisposition to CCD. However, a higher prevalence of advanced cognitive dysfunction has been reported in small-breed dogs. This is believed to be due to their longer life expectancy [316].

The heterogeneous presentation and slow, insidious onset of clinical signs make CCD particularly challenging to diagnose in veterinary medicine. To support consistent identification and standardized documentation of behavioral changes, the DISHAA acronym has been widely adopted as a practical clinical framework, encompassing the principal domains affected by the disease: disorientation, altered social interactions, disturbances of the sleep–wake cycle, house-soiling, changes in activity, and increased anxiety [2,3].

Disorientation is one of the most commonly reported and recognizable features of CCD. Affected dogs frequently appear confused within previously familiar environments, including their own homes or gardens [1,2]. Typical behaviors include aimless staring, becoming trapped in corners or behind furniture, and impaired spatial navigation. Deficits in learned responses, including failure to respond to familiar commands or the dog’s name, indicate underlying impairments in memory and executive function (Figure 4) [180,317,318].

Social behavioral alterations are a prominent feature of CCD and may significantly compromise the human–animal bond. Many affected dogs show reduced social engagement, manifested by diminished greeting behavior, withdrawal from physical contact, or apparent apathy toward owners and other animals [1,2,319]. In contrast, some dogs develop excessive attachment and attention-seeking behavior, often described as increased “clinginess.” In certain cases, irritability or uncharacteristic aggression may also emerge, likely reflecting impaired emotional regulation and a reduced tolerance to environmental stimuli.

Disturbances of the sleep–wake cycle are widely regarded as a hallmark of CCD and closely mirror circadian rhythm disruption observed in human neurodegenerative disorders [3,318]. Affected dogs frequently sleep excessively during the day while exhibiting nocturnal restlessness, pacing, or vocalization [1]. These behavioral changes are particularly disruptive for owners and often represent a primary reason for seeking veterinary consultation.

House-soiling constitutes another highly distressing manifestation of CCD. Dogs may urinate or defecate indoors in inappropriate locations despite a long-standing history of reliable housetraining [3,180,317]. Rather than reflecting limited access to outdoor elimination areas, this behavior results from cognitive deficits affecting spatial awareness, memory, and appropriate signaling.

Alterations in activity levels are also commonly reported, although the direction of change varies among individuals. Many dogs exhibit a reduction in purposeful activity, with diminished interest in exploration, play, and social interaction, consistent with apathy and motivational impairment [2,3].

**Figure 5:**
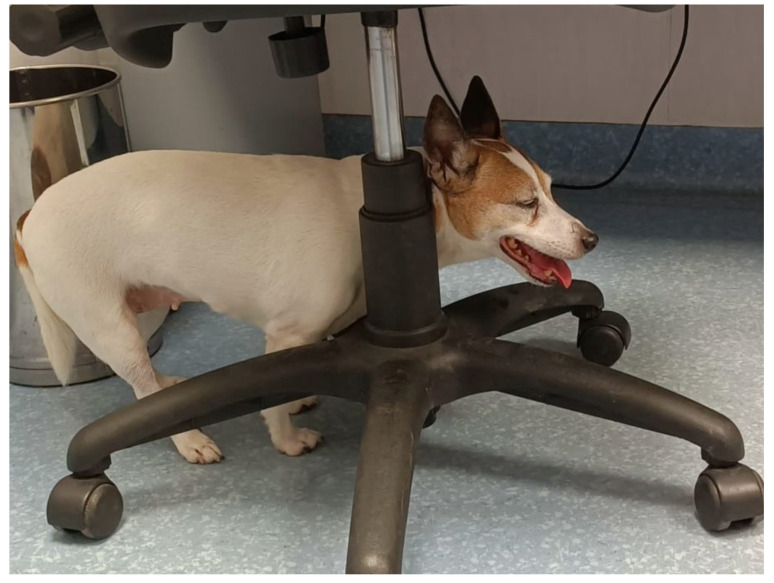
The image shows an 11-year-old female Jack Russell terrier with cognitive dysfunction, stuck between the legs of a chair and unable to get out due to severe disorientation.

In contrast, some dogs develop repetitive, non-goal-directed behaviors such as pacing, circling, or fixed-route wandering (Figure 5), which may reflect dysfunction of cortical–subcortical circuits involved in behavioral inhibition [320].

Increased anxiety is increasingly recognized as a central component of CCD rather than a secondary consequence of aging. Dogs may develop heightened sensitivity to previously tolerated stimuli, new-onset phobias, or generalized anxiety [1,321]. Notably, separation anxiety may newly emerge or worsen in cognitively impaired dogs, resulting in substantial welfare implications for both the animal and the owner [321]. Anxiety-related behaviors contribute significantly to reduced quality of life and may accelerate caregiver burden.

Despite growing awareness, CCD lacks a definitive antemortem diagnostic test. Consequently, diagnosis remains one of exclusion and requires a comprehensive and systematic approach to rule out alternative causes of behavioral change [1,322]. The absence of a standardized diagnostic protocol has been widely acknowledged as a major limitation in both clinical practice and research settings [322].

The diagnostic process begins with detailed history taking and thorough physical and neurological examination. Numerous conditions can mimic or exacerbate CCD-like behaviors, including endocrine disorders such as hypothyroidism and hyperadrenocorticism, sensory decline due to vision or hearing loss, chronic pain—particularly osteoarthritis—and metabolic or organ system disease [2,321,322]. Intracranial disease, including primary or secondary brain neoplasia, must be carefully excluded, especially in cases with acute onset or focal neurological deficits.

Baseline diagnostic testing typically includes a complete blood count, serum biochemistry profile, urinalysis, thyroid hormone measurement and blood pressure assessment [2,317]. When routine diagnostics fail to identify an alternative cause and clinical suspicion remains high, advanced neuroimaging is recommended.

Given the behavioral nature of CCD, owner-reported assessment tools form the foundation of antemortem diagnosis. Structured questionnaires provide a systematic method for quantifying the presence and severity of behavioral abnormalities and reduce reliance on anecdotal reporting [1,180,317]. Among the available instruments, the Canine Cognitive Dysfunction Rating (CCDR) scale and the Canine DEmentia Scale (CADES) are the most widely validated [319,323]. While the CCDR is well established for identifying affected dogs, the CADES appears particularly useful for disease staging and detection of early cognitive impairment, as well as longitudinal monitoring of disease progression [2]. Nevertheless, all questionnaire-based tools remain inherently subjective and influenced by owner perception and awareness [319].

Magnetic resonance imaging (MRI) plays an important adjunctive role in CCD diagnosis. Its primary function is to exclude other structural brain disorders, including neoplasia, inflammatory disease and cerebrovascular accidents [2,314]. In addition, MRI may provide supportive evidence of CCD through identification of age-inappropriate cerebral atrophy, ventricular enlargement and widening of cortical sulci [180,317,324]. Quantitative MRI studies have demonstrated significant hippocampal volume loss in dogs with CCD, paralleling a key imaging biomarker of human Alzheimer’s disease [325]. However, overlap with normal aging currently limits the diagnostic utility of hippocampal volumetry at the individual level. Current research increasingly focuses on identifying fluid-based biomarkers that reflect CCD neuropathology and may enable earlier, more objective diagnosis. Amyloid-β, neurofilament light chain, and glial fibrillary acidic protein have emerged as leading candidates. These biomarkers reflect cerebral amyloidosis, neuroaxonal damage, and astroglial activation, respectively [157,326]. While promising, these biomarkers lack sufficient specificity when used in isolation. Consequently, current research is exploring multimarker panels and machine learning-based approaches to improve diagnostic accuracy using minimally invasive blood samples [317]. Collectively, advances in behavioral assessment, neuroimaging and biomarker research are progressively refining the diagnostic landscape of CCD. Integration of these modalities will be critical to improve early detection, guide intervention strategies, and enhance the welfare of aging companion dogs.

## 10. Magnetic Resonance Imaging

MRI represents the imaging modality of choice for the evaluation of senile brain degeneration (Canine Cognitive Dysfunction) in dogs.

Signs of brain atrophy include widened and well-demarcated cerebral sulci, ventricular enlargement, and a reduced thickness of the interthalamic adhesion [327].

It was observed that the thickness of the interthalamic adhesion as measured on transaxial T1-weighted and T2-weighted MRI was significantly smaller in dogs with CCD compared with dogs without CCD; an interthalamic adhesion thickness of 5 mm or less was found to be consistent with a diagnosis of CCD [219,328].

**Figure 6:**
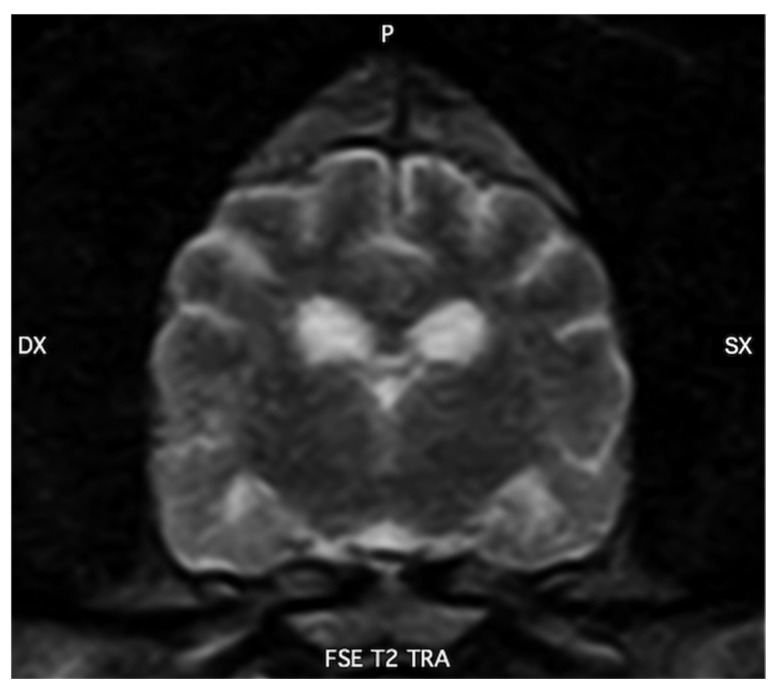
Dog, mixed breed, 15 y old, male castrated. Transverse T2-weighted image (0.25 T MRI unit, Esaote, Italy). Well demarcated cerebral sulci suggest brain atrophy. Mild ventricular enlargement.

A more recent study confirmed the interthalamic adhesion thickness measurement in CCD. It also demonstrated that the ratio of interthalamic adhesion thickness to brain height as well as the ratio between this value and the lateral ventricle height–to–brain height ratio were accurate predictors of CCD [219,329]. Spontaneous intraparenchymal brain hemorrhage has been documented in both AD and CCD. Although MRI evidence of microhemorrhages is common in patients with AD, the prevalence of this finding in dogs with CCD has not been specifically investigated [219]. T2*-weighted MRI sequence has been shown to be useful in identifying hemorrhagic brain lesions in both people and dogs. In people with AD, MRI evidence of microhemorrhages on T2*-weighted imaging is common and is attributed to cerebrovascular amyloid angiopathy due to accumulation in the brain of β-amyloid protein, which accumulates in the brain of dogs with CCD and humans with AD and forms plaques within the brain parenchyma and also contributes to cerebrovascular disease [205,206,219,330,331,332]. Occasionally, macrohemorrhages are also evident on T2*-weighted images in these patients [219,333]. In a retrospective investigation of brain microhemorrhages in dogs that were imaged with T2*-weighted MRI sequences, older dogs of smaller breeds were significantly more represented. Microhemorrhages, typically evident on T2*-weighted images of dogs’ brains, were more common in older, smaller breeds, often seen with brain atrophy (Figure 6), proteinuria, and vestibular signs [219,334].

**Figure 7:**
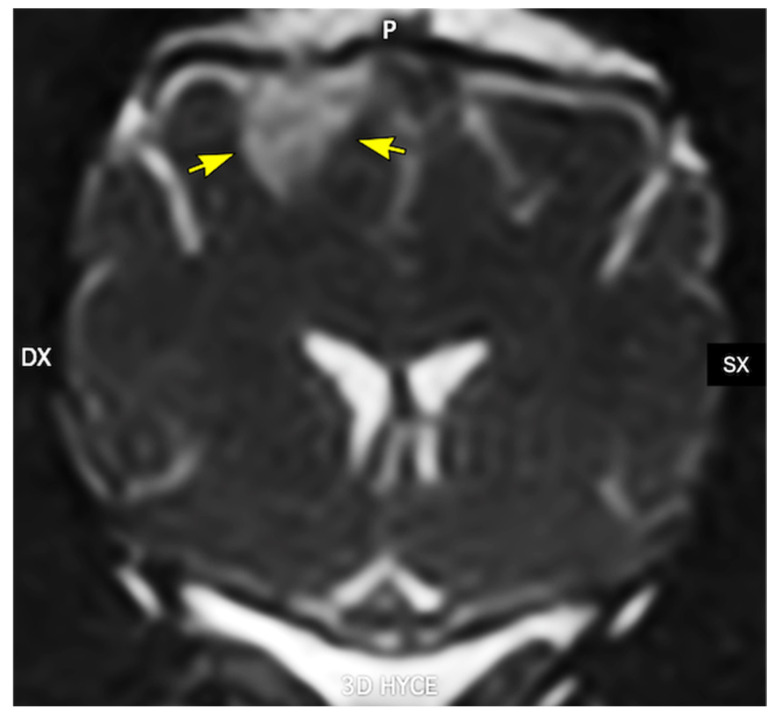
Dog, mixed breed, 15 y old, male castrated. Three-dimensional Hyce image–transverse MPR, 1 mm slice thickness (0.25 T MRI unit, Esaote, Italy). A triangular shaped hyperintense area on the right side of the cortex of the temporal lobe is suggestive of vascular disease (yellow arrows).

The identification of white matter hyperintensities (Figure 7) (white spots in T2-weighted/FLAIR images), usually periventricular, called leukoaraiosis, suggestive of small-vessel diseases/demyelination, may indicate senile brain degeneration [219,335].

**Figure 8:**
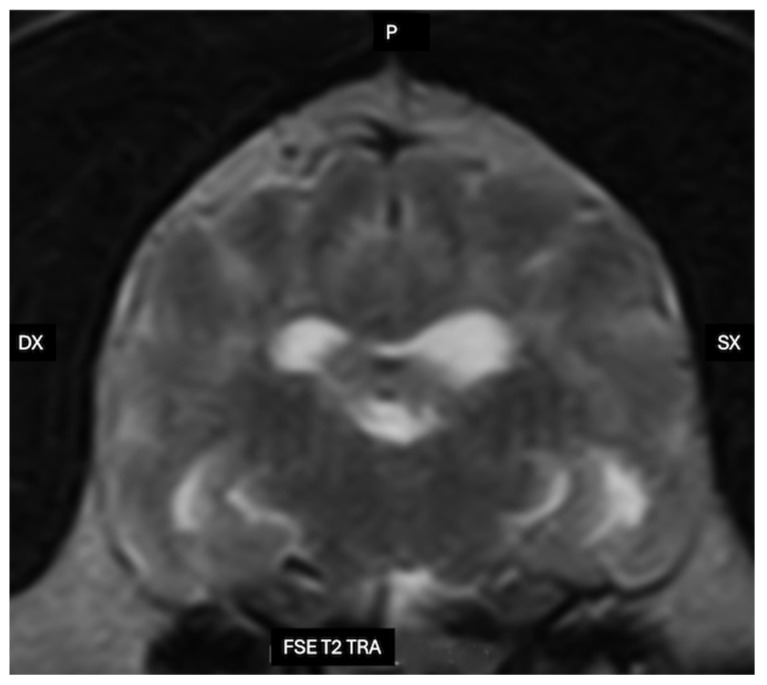
Dog, Drahathar, 14 y old, male castrated. Transverse T2-weighted image (0.25 T MRI unit, Esaote, Italy). No sign of brain atrophy. Mild ventricular enlargement.

MRI brain imaging of patients with CCD can also be normal (false negative findings) (Figure 8) or may reveal, as previously described, brain atrophy, ventricular enlargement, widened and well-demarcated cerebral sulci, and brain lesions particularly evident in the medial temporal lobes of the cerebral cortex [219]. On the other hand, consistent MRI findings associated with brain aging can also be found in some older patients without evidence of CCD (false positive).

## 11. Conclusions

Canine cognitive dysfunction and human Alzheimer’s disease share a broad set of structural, molecular, and clinical features that support viewing them as points along a common spectrum of age-related neurodegeneration rather than entirely separate entities. In both species, progressive cortical and hippocampal atrophy, synaptic and neuronal loss, β-amyloid accumulation in parenchyma and vessel walls, oxidative stress, mitochondrial dysfunction, and chronic neuroinflammation converge to drive cognitive decline.

In contrast to human Alzheimer’s disease, dogs typically exhibit a predominantly amyloid-β-centric pathology with relatively limited and less stereotyped tau involvement, whereas AD is defined by combined Aβ–tau proteinopathy characterized by extensive neurofibrillary tangle formation and well-defined Braak stage progression. A central mechanism unifying both conditions is dysfunction of brain clearance systems, particularly the glymphatic pathway and intramural periarterial drainage. Aging, small-vessel disease, cerebral amyloid angiopathy, astrocytic gliosis, and disruption or mislocalization of aquaporin-4 at astrocytic endfeet impair cerebrospinal fluid–interstitial fluid exchange and perivascular transport, thereby promoting Aβ and tau retention and aggregation [282]. Anatomical, magnetic resonance imaging, and ultrastructural studies indicate that dogs share the fundamental components of these clearance pathways with humans, including Virchow–Robin spaces, basement membrane-based drainage routes, and polarized perivascular AQP4 expression. This close anatomical and functional correspondence makes CCD particularly well suited for investigating glymphatic dysfunction and the interplay between vascular pathology and protein aggregation in a large, spontaneously affected brain [300].

Clinically, CCD presents as a progressive and multifaceted behavioral syndrome captured by the DISHAA framework, encompassing disorientation, altered social interactions, disturbances of the sleep–wake cycle, house-soiling, changes in activity, and increased anxiety. Neuroimaging findings, including cortical and hippocampal atrophy, ventricular enlargement, reduced interthalamic adhesion thickness, white matter hyperintensities, and cerebral microhemorrhages, closely parallel established imaging biomarkers of AD and provide objective correlates of owner-reported cognitive and behavioral changes. At the ultrastructural level, transmission electron microscopy in both species reveals convergent signatures of neurodegeneration, such as amyloid fibrils, synaptic degeneration, mitochondrial abnormalities, and age-related myelin and axonal pathology. At the same time, important interspecies differences must be acknowledged, particularly the greater prominence of tau pathology and the well-defined genetic risk architecture of human AD (including APOE ε4 and PSEN1/2 mutations), contrasted with the current absence of equivalent major risk alleles in CCD. Consequently, CCD more closely models the early to intermediate, Aβ-dominated stages of human Alzheimer’s disease rather than its late, tau-heavy phases. Taken together, the available comparative evidence supports aged dogs with CCD as a powerful and complementary translational model for AD.

Canine Cognitive Dysfunction offers:

(a) A naturally occurring, environmentally relevant platform to study how Aβ, vascular pathology, and impaired glymphatic/perivascular clearance interact over time.
(b) The opportunity to link behavior, advanced imaging, fluid biomarkers, and ultrastructure in ways that are difficult to achieve in humans and not fully recapitulated in rodent models.
(c) A clinically meaningful setting in which to test multimodal interventions aimed at reducing Aβ burden, modulating tau phosphorylation and aggregation, restoring neurovascular and glymphatic function, and supporting synaptic and mitochondrial health [280].

Future work should prioritize (1) standardizing neuropathological and imaging staging frameworks for CCD analogous to NIA–AA “ABC” criteria; (2) refining and validating behavioral and owner-reported scales for early detection and longitudinal monitoring; (3) developing and testing blood- and CSF-based biomarker panels that integrate Aβ, tau, neuroaxonal and glial markers; and (4) systematically evaluating therapies that target clearance pathways, vascular integrity, and protein aggregation in longitudinal canine cohorts.

Integrating pathology, imaging, clinical assessment, and biomarker research in dogs and humans will clarify shared mechanisms of neurodegeneration and accelerate the development of disease-modifying strategies for both CCD and Alzheimer’s disease.

## Figures and Tables

**Table 2:** Human and Canine Tau Isoforms.

Item/Disease	Exon 10 (R2) Status	Tau Repeat Type in Fibrils	Main Isoforms/Pool	Predominant Isoform Composition	Notes/Key References
Structural rule	Exon 10 excluded	3R	0N3R, 1N3R, 2N3R	—	Exon 10− → loss of R2 → 3-repeat tau (3R) [229,235]
Structural rule	Exon 10 included	4R	0N4R, 1N4R, 2N4R	—	Exon 10+ → inclusion of R2 → 4-repeat tau (4R) [229,235]
Adult human brain tau	Both exon 10− and exon 10+	3R + 4R	Six major isoforms (352–441 aa)	Mixed 3R + 4R pool	MAPT alternative splicing generates 0N/1N/2N × 3R/4R [229].
Alzheimer’s disease (AD)	Mixed exon 10−/exon 10+	3R + 4R	Mixed 3R and 4R isoforms in filaments	Mixed 3R + 4R	Classic mixed 3R/4R tauopathy [235].
Primary age-related tauopathy (PART)	Mixed exon 10−/exon 10+	3R + 4R	Mixed 3R and 4R	Mixed 3R + 4R	AD-like but with more restricted distribution [235].
Chronic traumatic encephalopathy (CTE)	Mixed exon 10−/exon 10+	3R + 4R	Mixed 3R and 4R	Mixed 3R + 4R	Trauma-associated mixed 3R/4R fold [235].
Pick’s disease (PiD)	Mainly exon 10−	Predominantly 3R	3R-only filaments	3R	Prototypical 3R-dominant tauopathy [235].
Corticobasal degeneration (CBD)	Mainly exon 10+	Predominantly 4R	4R-only filaments	4R	Prototypical 4R tauopathy [235].
Argyrophilic grain disease (AGD)	Mainly exon 10+	Predominantly 4R	4R-only filaments	4R	4R-dominant tauopathy [235].
Progressive supranuclear palsy (PSP)	Mainly exon 10+	Predominantly 4R	4R-only filaments	4R	4R tauopathy with characteristic PSP fold [235].
Globular glial tauopathy (GGT)	Mainly exon 10+	Predominantly 4R	4R-only filaments	4R	4R-dominant glial tauopathy [235]
Canine cognitive dysfunction (CCD)	Not structurally resolved (AD-like)	Likely mixed 3R + 4R pool ***	Canine tau orthologues of 3R/4R isoforms	AD-like, presumed 3R + 4R	Hyperphosphorylated/aggregated tau with AD-like pathology; 3R/4R fibril composition not yet defined [185,236]

Legend: Human: six canonical brain isoforms—0N3R, 1N3R, 2N3R; 0N4R, 1N4R, 2N4R (352–441 aa); Canine: CCD shows AD-like tau pathology; *** the exact 3R/4R fibril composition and high-resolution filament structure have not yet been determined by cryo-EM.

**Table 3:** Steps of Prion-Like Propagation of Tau in Alzheimer’s Disease and Canine Cognitive Dysfunction.

Step	Element	Key Details	References
1	Initial tau misfolding	Soluble tau → misfolded/aggregated tau assemblies; early sites: transentorhinal cortex, hippocampus	[246]
2	Release of misfolded tau from affected neurons	Active release: exocytosis, ectosomes (microvesicles), exosomes; passive release: leakage from degenerating neurons	[251]
3	Extracellular tau seeds in brain interstitium	Misfolded tau persists in extracellular space; in chronic vascular insufficiency, BBB compromise, low-grade neuroinflammation → enhanced extracellular persistence and interstitial transport	[251,253,254]
4	Uptake of tau seeds by neighboring cells	Mechanisms: endocytosis, macropinocytosis, receptor-mediated uptake	[251]
5	Intracellular seeding and templated misfolding	Internalized seeds induce misfolding of endogenous soluble tau; amplification of misfolded/aggregated tau within neuron	[251,252]
6	Spread along anatomically connected networks	Seed-containing neurons release new tau assemblies; spread via synaptic and network connections; mirrors Braak staging: transentorhinal/hippocampal regions → association cortices → primary sensory–motor areas	[246]
7	Regional tau pathology and clinical progression	Progressive accumulation of tau pathology in connected regions; neurodegeneration and cognitive decline; in chronic vascular insufficiency: vascular, BBB, and neuroinflammatory factors further facilitate propagation	[246,253,254,255]

**Table 4:** Neuropathology of Alzheimer’s Disease vs. Canine Cognitive Dysfunction.

Aspect	Alzheimer’s Disease (AD)	Canine Cognitive Dysfunction (CCD)	Key References
Gross lesions—distribution and atrophy pattern	Marked diffuse cortical atrophy, most pronounced in medial temporal lobes (hippocampus, entorhinal cortex) and association neocortex; relative early sparing of primary motor/sensory cortex; hippocampus and amygdala shrunken; enlarged ventricles (ex vacuo hydrocephalus); atrophy of basal forebrain (nucleus basalis of Meynert).	Generalized cortical atrophy, usually less severe than in advanced AD; sulcal widening and ventricular enlargement; atrophy particularly in frontal and temporal (± parietal) cortex; hippocampal volume loss correlates with cognitive decline; cerebellum relatively spared.	[6,9,10,185,246,305,306,313]
Gross lesions—vascular/hemorrhagic changes	Cerebral amyloid angiopathy (CAA) frequent; may cause lobar microbleeds or macrohemorrhages, cortical superficial siderosis; more prevalent/severe in some genetic backgrounds (e.g., APOE ε4).	CAA common in aged dogs with or without CCD; Aβ in leptomeningeal and cortical vessel walls; associated with microbleeds and vasculopathy; overt large hemorrhages less systematically described than in human AD.	[6,7,307,308,311]
Histopathology—Aβ plaques	Abundant Aβ plaques: diffuse and neuritic (cored) plaques. Neuritic plaques have dense fibrillar Aβ core with dystrophic neurites, microglia, astrocytes. Early deposition in neocortex → limbic → subcortical areas (Thal phases 1–5). Aβ40 and Aβ42 species present; Aβ42 enriched in parenchymal plaques.	Aβ deposition is the most consistent lesion: mainly in association cortices (frontal, temporal, parietal) and less in hippocampus. Diffuse plaques common in aged dogs; cored/neuritic plaques more frequent or abundant in clinically affected CCD cases. Aβ42 predominates in plaques; Aβ40 more in vascular deposits.	[5,9,11,220,259,305]
Histopathology—Tau/NFTs	Defining lesion: abundant neurofibrillary tangles (NFTs) composed of hyperphosphorylated tau (paired helical filaments) in neuronal soma and dendrites; associated neuropil threads and tau-positive dystrophic neurites. Stereotyped progression (Braak stages I–VI) from transentorhinal/entorhinal → hippocampus/limbic → widespread neocortex. NFT/tau burden correlates strongly with cognitive decline.	Classic NFTs largely absent or very sparse. Phospho-tau-positive neurons/threads occasionally reported but without widespread, layered Braak-like progression. No standardized staging system analogous to Braak in dogs. CCD is therefore considered primarily an Aβ-centric pathology with limited tauopathy.	[185,246,309,310]
Histopathology—neuronal & synaptic changes	Marked neuronal loss in hippocampal CA1, entorhinal cortex, association cortices, and basal forebrain cholinergic nuclei; prominent synapse loss (e.g., in frontal and temporal cortex) tightly correlates with severity of dementia.	Neuronal loss/shrinkage in frontal and temporal cortex and hippocampus, but generally less dramatic and more variable; synaptic loss (e.g., decreased synaptophysin) correlates with cognitive deficits; white matter degeneration/demyelination, especially frontal, can contribute to dysfunction.	[9,10,185,205,210,268,306]
Histopathology—glial and inflammatory changes	Robust astrogliosis and microgliosis surrounding plaques and tangles; chronic innate immune activation in affected regions; microglia often clustered around plaques, astrocytes hypertrophic and GFAP-positive; modest white matter degeneration.	Astrogliosis and microgliosis often associated with cortical Aβ plaques and CAA; gliosis more variable but present in regions with heavy Aβ burden; white matter gliosis and degeneration described in aged dogs. Extent of neuroinflammation generally less well characterized than in human AD.	[5,9,205,220,278]
Histopathology—vascular pathology	CAA common: Aβ (especially Aβ40) in leptomeningeal and cortical vessel walls, sometimes with smooth muscle cell loss and fibrosis; associated microbleeds, microinfarcts, superficial siderosis; often coexists with small-vessel disease.	CAA frequent in aged dogs: Aβ in cortical and leptomeningeal arterioles/arteries; associated wall thickening, smooth muscle degeneration, and occasional microbleeds; microinfarcts and small-vessel disease less systematically described but present in some series.	[6,7,307,308,311]
Immunohistochemistry—Aβ	Anti-Aβ antibodies (e.g., 4G8, 6E10, Aβ40, Aβ42) used to detect diffuse and cored plaques and CAA; support Thal amyloid phase scoring; enable quantification of plaque burden and vascular deposition.	Human Aβ antibodies cross-react with canine Aβ; 4G8, 6E10, Aβ42, Aβ40 commonly used to grade plaque and CAA burden; IHC confirms predominance of Aβ42 in plaques and Aβ40 in vessels; used to correlate lesion load with cognitive scores.	[5,11,220,259,305,312]
Immunohistochemistry—tau	Phospho-tau antibodies (AT8, PHF-1, CP13, etc.) robustly label NFTs, neuropil threads, and dystrophic neurites; applied to assign Braak NFT stages (NIA-AA “B” score).	Phospho-tau antibodies (e.g., AT8) usually show minimal to focal immunoreactivity; classic NFT morphology and extensive distribution are rare; no established canine tau staging scheme.	[185,246,305,309,312]
Immunohistochemistry—neuronal & synaptic markers	NeuN, MAP2 for neuronal cell bodies and dendrites; synaptophysin, PSD-95 for synapses; reduced immunoreactivity in hippocampus and association cortex correlates with cognitive impairment.	NeuN/MAP2 used to document neuronal loss/atrophy; synaptophysin and other synaptic markers diminished in frontal cortex and hippocampus in CCD, correlating with behavioral measures of cognitive dysfunction.	[9,10,185,210,268]
Immunohistochemistry—glial markers	GFAP stains reactive astrocytes; Iba1, CD68, and other microglial markers highlight activated microglia surrounding plaques and tangles; support assessment of neuroinflammation.	GFAP and Iba1/CD68 demonstrate plaque-associated astrocytosis and microgliosis; patterns broadly similar to AD but often less extensive and less systematically staged in the literature.	[5,9,220,278]
Immunohistochemistry—integrated staging frameworks	NIA–AA “ABC” scheme: A = Thal Aβ phase; B = Braak NFT stage; C = CERAD neuritic plaque score; IHC for Aβ and phospho-tau central to classification.	No universally accepted standardized staging comparable to NIA–AA. Most studies use semi-quantitative or regional scoring of Aβ plaques and CAA, with limited tau assessment and without a formal composite stage.	[220,305,312]

**Table 5:** Pathological Similarities and Differences Between Alzheimer’s Disease and Canine Cognitive Dysfunction.

Dimension	Similarities (AD-CCD)	Differences (AD vs. CCD)	References
Global brain atrophy	Both show age-associated cortical and hippocampal atrophy, with ventricular enlargement and sulcal widening. Hippocampal volume loss correlates with cognitive decline in both species.	AD typically exhibits more severe and widespread atrophy in advanced stages, especially in medial temporal structures and association neocortex; CCD atrophy tends to be milder and more variable, often accentuated in frontal lobes.	[9,10,185,246,305,313]
Aβ plaque pathology	Both develop cortical Aβ plaques (diffuse and cored/neuritic), particularly in association cortices; Aβ42 is prominent in parenchymal plaques in both species. Plaque burden generally correlates with aspects of cognitive impairment.	AD usually shows a more stereotyped spatiotemporal evolution of Aβ deposition (Thal phases) and frequently high neuritic plaque density; CCD may have abundant diffuse plaques in cognitively normal aged dogs, with less clear threshold between “normal aging” and disease; overall neuritic change tends to be less pronounced than in advanced AD.	[5,9,11,220,259]
Tau pathology	Both species express tau and can show phospho-tau immunoreactivity, and isolated tau-positive neurons/threads may appear in aged dog brains.	AD is defined by abundant hyperphosphorylated tau NFTs, neuropil threads, and tau-positive dystrophic neurites, with well-defined Braak staging that strongly correlates with cognitive decline; CCD typically lacks classic NFTs and does not show a Braak-like laminar progression; tau pathology is sparse, inconsistent, and not central to diagnosis.	[185,246,309,310]
Neuronal and synaptic degeneration	Both exhibit neuronal loss and synaptic depletion in cortex and hippocampus; synapse loss correlates with cognitive dysfunction in each disease. Both show neuron shrinkage and dendritic abnormalities in affected regions.	Neuronal and synaptic loss are generally more extensive and stereotyped in AD, involving hippocampus, entorhinal cortex, multimodal association cortices, and cholinergic basal forebrain nuclei. In CCD, neuronal and synaptic loss can be significant but is more variable, and it is harder to distinguish from changes of “normal” canine aging; cholinergic system involvement is less well characterized.	[9,185,205,210,268,306]
Glial response and neuroinflammation	Both show plaque-associated astrocytosis and microgliosis; GFAP-positive astrocytes and Iba1/CD68-positive microglia cluster around Aβ deposits; chronic low-grade neuroinflammation is a shared feature.	In AD, microglial and astrocytic activation has been deeply characterized and linked to genetic risk (e.g., TREM2, APOE) and disease progression; in CCD, glial responses are documented but less comprehensively studied, and their temporal dynamics and causal contribution to cognitive decline remain less clear.	[5,9,205,220,278]
Vascular Aβ and CAA	Both develop CAA with Aβ deposition in leptomeningeal and cortical vessel walls; Aβ40 frequently enriched in vascular deposits; CAA can be associated with microbleeds and microinfarcts in both species.	In AD, the clinical and pathological spectrum of CAA (microbleeds, macrohemorrhages, superficial siderosis, ischemic lesions) is well defined and integrated into mixed-dementia concepts; in CCD, CAA is common but the full clinical impact and relationship to cognitive signs are less clearly delineated and less systematically quantified.	[6,7,307,308,311]
Clinicopathologic correlation & staging frameworks	In both, increasing cortical/hippocampal pathology (Aβ, neuronal/synaptic loss) tracks with worsening cognition; aged but cognitively normal individuals may still have some Aβ burden.	AD has formal NIA–AA neuropathologic criteria (ABC score) integrating Aβ phase, NFT stage, and neuritic plaque density; cognition correlates particularly with Braak stage and synaptic loss. CCD lacks standardized staging; most studies apply semi-quantitative scoring for Aβ and gliosis, with limited tau assessment, making cross-study comparisons and clinicopathologic correlations less robust.	[210,220,305,312]
Overall disease “type” (proteinopathy profile)	Combined Aβ- and tau-driven proteinopathy with strong tau contribution to neurodegenerative cascade; often accompanied by vascular and other co-pathologies in aged humans.	Largely Aβ-dominant encephalopathy with prominent plaques and CAA but minimal classic tauopathy; therefore, considered a partial analog of AD, modeling especially the amyloid and vascular aspects rather than the full tau-driven neurodegenerative spectrum.	[9,220,246,259]

**Table 6:** Translational impact of TEM-based findings in Alzheimer’s disease and canine cognitive dysfunction.

Pathological Feature (TEM)	Ultrastructural Observations	Translational Impact	References
Amyloid fibrils and plaques	Fibrillar Aβ deposits with defined diameter, periodicity, and packing in human cerebrovascular and parenchymal amyloid; analogous Aβ fibrils within plaques and vessel walls in aged dogs	Confirms that dogs develop human-like Aβ fibrils and cerebral amyloid angiopathy, supporting aged dogs as a spontaneous large-animal model for amyloidogenesis and anti-amyloid therapies	[6,210,220,245]
Neurofibrillary tangles and tau filaments	Paired helical filaments and straight tau filaments with conserved ultrastructural motifs in human AD; age-related tau alterations documented in canine brain	Structural conservation of tau assemblies informs cross-species mechanisms of tauopathy, enabling preclinical evaluation of tau-targeting agents in dogs that model early or limited tau pathology	[220,245,315]
Synaptic degeneration	Reduced synaptic density, dystrophic synaptic profiles, altered vesicle pools, and associated mitochondrial abnormalities in AD cortex and hippocampus; comparable synaptic degeneration in CCD	Establishes synapse loss and ultrastructural synaptic pathology as common correlates of cognitive decline in humans and dogs, supporting synaptic metrics as translational outcome measures for disease-modifying interventions	[5,6,210,220]
Mitochondrial and autophagic changes	Abnormal mitochondrial morphology (swelling, cristae disruption) and accumulation of autophagic vacuoles in neurons and neurites in AD; similar mitochondrial alterations described in aged/CCD canine brain	Supports conserved pathways of bioenergetic failure and impaired proteostasis across species, justifying the use of dogs for testing mitochondrial and autophagy-modulating therapies	[210,220]
Myelin and axonal pathology	Axonal swellings, spheroids, myelin splitting, and degeneration in aging human white matter; TEM evidence of analogous age-related axon/myelin pathology in dogs	Provides a structural basis for shared white matter dysfunction and cerebrovascular contributions to cognitive impairment, enabling translational studies of vascular and myelin-protective strategies	[6,10,220]
Correlation of Aβ burden with cognition	Quantitative TEM and histopathology show that increasing amyloid load and neuritic pathology correlate with cognitive deficits in both species	Validates aged dogs as a model of early–intermediate AD stages, in which Aβ load and neuritic pathology can be linked to behavioral measures, improving prediction of clinical efficacy from preclinical trials	[1,5,185,219]

## Data Availability

No new data were created or analyzed in this study. Data sharing is not applicable to this article.

## References

[1] Fast R., Schütt T., Toft N., Møller A., Berendt M. (2013). An Observational Study with Long-Term Follow-up of Canine Cognitive Dysfunction: Clinical Characteristics, Survival, and Risk Factors. J. Vet. Intern. Med..

[2] Vite C.H., Head E. (2014). Aging in the Canine and Feline Brain. Vet. Clin. N. Am. Small Anim. Pract..

[3] Blanchard T., Eppe J., Mugnier A., Delfour F., Meynadier A. (2025). Enhancing Cognitive Functions in Aged Dogs and Cats: A Systematic Review of Enriched Diets and Nutraceuticals. Geroscience.

[4] Prpar Mihevc S., Majdič G. (2019). Canine Cognitive Dysfunction and Alzheimer’s Disease—Two Facets of the Same Disease?. Front. Neurosci..

[5] Cummings B.J., Head E., Afagh A.J., Milgram N.W., Cotman C.W. (1996). Beta-Amyloid Accumulation Correlates with Cognitive Dysfunction in the Aged Canine. Neurobiol. Learn. Mem..

[6] Colle M.A., Hauw J.J., Crespeau F., Uchihara T., Akiyama H., Checler F., Pageat P., Duykaerts C. (2000). Vascular and Parenchymal Abeta Deposition in the Aging Dog: Correlation with Behavior. Neurobiol. Aging.

[7] Uchida K., Nakayama H., Goto N. (1991). Pathological Studies on Cerebral Amyloid Angiopathy, Senile Plaques and Amyloid Deposition in Visceral Organs in Aged Dogs. J. Vet. Med. Sci..

[8] Head E., Liu J., Hagen T.M., Muggenburg B.A., Milgram N.W., Ames B.N., Cotman C.W. (2002). Oxidative Damage Increases with Age in a Canine Model of Human Brain Aging. J. Neurochem..

[9] Serrano-Pozo A., Frosch M.P., Masliah E., Hyman B.T. (2011). Neuropathological Alterations in Alzheimer Disease. Cold Spring Harb. Perspect. Med..

[10] Head E., Rofina J., Zicker S. (2008). Oxidative Stress, Aging, and Central Nervous System Disease in the Canine Model of Human Brain Aging. Vet. Clin. N. Am. Small Anim. Pract..

[11] Head E., McCleary R., Hahn F.F., Milgram N.W., Cotman C.W. (2000). Region-Specific Age at Onset of Beta-Amyloid in Dogs. Neurobiol. Aging.

[12] Jessen N.A., Munk A.S.F., Lundgaard I., Nedergaard M. (2015). The Glymphatic System: A Beginner’s Guide. Neurochem. Res..

[13] Iliff J.J., Wang M., Liao Y., Plogg B.A., Peng W., Gundersen G.A., Benveniste H., Vates G.E., Deane R., Goldman S.A. (2012). A Paravascular Pathway Facilitates CSF Flow through the Brain Parenchyma and the Clearance of Interstitial Solutes, Including Amyloid β. Sci. Transl. Med..

[14] Iliff J.J., Wang M., Zeppenfeld D.M., Venkataraman A., Plog B.A., Liao Y., Deane R., Nedergaard M. (2013). Cerebral Arterial Pulsation Drives Paravascular CSF-Interstitial Fluid Exchange in the Murine Brain. J. Neurosci..

[15] Mestre H., Hablitz L.M., Xavier A.L., Feng W., Zou W., Pu T., Monai H., Murlidharan G., Rivera R.M.C., Simon M.J. (2018). Aquaporin-4-Dependent Glymphatic Solute Transport in the Rodent Brain. eLife.

[16] de Lahunta A., Glass E., Kent M. (2021). Veterinary Neuroanatomy and Clinical Neurology.

[17] Taoka T., Masutani Y., Kawai H., Nakane T., Matsuoka K., Yasuno F., Kishimoto T., Naganawa S. (2017). Evaluation of Glymphatic System Activity with the Diffusion MR Technique: Diffusion Tensor Image Analysis along the Perivascular Space (DTI-ALPS) in Alzheimer’s Disease Cases. Jpn. J. Radiol..

[18] Nielsen S., Nagelhus E.A., Amiry-Moghaddam M., Bourque C., Agre P., Ottersen O.P. (1997). Specialized Membrane Domains for Water Transport in Glial Cells: High-Resolution Immunogold Cytochemistry of Aquaporin-4 in Rat Brain. J. Neurosci..

[19] Nagelhus E.A., Ottersen O.P. (2013). Physiological Roles of Aquaporin-4 in Brain. Physiol. Rev..

[20] Papadopoulos M.C., Verkman A.S. (2013). Aquaporin Water Channels in the Nervous System. Nat. Rev. Neurosci..

[21] Iliff J.J., Chen M.J., Plog B.A., Zeppenfeld D.M., Soltero M., Yang L., Singh I., Deane R., Nedergaard M. (2014). Impairment of Glymphatic Pathway Function Promotes Tau Pathology after Traumatic Brain Injury. J. Neurosci..

[22] Álvarez P., Blasco E., Pumarola M., Wessmann A. (2021). Aquaporin-4 Protein Expression in Normal Canine Brains. BMC Vet. Res..

[23] Kress B.T., Iliff J.J., Xia M., Wang M., Wei H.S., Zeppenfeld D., Xie L., Kang H., Xu Q., Liew J.A. (2014). Impairment of Paravascular Clearance Pathways in the Aging Brain. Ann. Neurol..

[24] Klostranec J.M., Vucevic D., Bhatia K.D., Kortman H.G.J., Krings T., Murphy K.P., terBrugge K.G., Mikulis D.J. (2021). Current Concepts in Intracranial Interstitial Fluid Transport and the Glymphatic System: Part I-Anatomy and Physiology. Radiology.

[25] Morris A.W.J., Sharp M.M., Albargothy N.J., Fernandes R., Hawkes C.A., Verma A., Weller R.O., Carare R.O. (2016). Vascular Basement Membranes as Pathways for the Passage of Fluid into and out of the Brain. Acta Neuropathol..

[26] Hasan-Olive M.M., Enger R., Hansson H.-A., Nagelhus E.A., Eide P.K. (2019). Loss of Perivascular Aquaporin-4 in Idiopathic Normal Pressure Hydrocephalus. Glia.

[27] Rasschaert M., Schroeder J.A., Wu T.-D., Marco S., Emerit A., Siegmund H., Fischer C., Fretellier N., Idée J.-M., Corot C. (2018). Multimodal Imaging Study of Gadolinium Presence in Rat Cerebellum: Differences Between Gd Chelates, Presence in the Virchow-Robin Space, Association with Lipofuscin, and Hypotheses About Distribution Pathway. Investig. Radiol..

[28] Harvey N.D. (2021). How Old Is My Dog? Identification of Rational Age Groupings in Pet Dogs Based Upon Normative Age-Linked Processes. Front. Vet. Sci..

[29] López-Otín C., Blasco M.A., Partridge L., Serrano M., Kroemer G. (2013). The Hallmarks of Aging. Cell.

[30] López-Otín C., Blasco M.A., Partridge L., Serrano M., Kroemer G. (2023). Hallmarks of Aging: An Expanding Universe. Cell.

[31] Guelfi G., Capaccia C., Tedeschi M., Bufalari A., Leonardi L., Cenci-Goga B., Maranesi M. (2024). Dog Aging: A Comprehensive Review of Molecular, Cellular, and Physiological Processes. Cells.

[32] Damkier H.H., Brown P.D., Praetorius J. (2013). Cerebrospinal Fluid Secretion by the Choroid Plexus. Physiol. Rev..

[33] Nielsen S., Smith B.L., Christensen E.I., Agre P. (1993). Distribution of the Aquaporin CHIP in Secretory and Resorptive Epithelia and Capillary Endothelia. Proc. Natl. Acad. Sci. USA.

[34] Damkier H.H., Praetorius J. (2012). Genetic Ablation of Slc4a10 Alters the Expression Pattern of Transporters Involved in Solute Movement in the Mouse Choroid Plexus. Am. J. Physiol. Cell Physiol..

[35] Oshio K., Watanabe H., Song Y., Verkman A.S., Manley G.T. (2005). Reduced Cerebrospinal Fluid Production and Intracranial Pressure in Mice Lacking Choroid Plexus Water Channel Aquaporin-1. FASEB J..

[36] Bulat M., Lupret V., Orehković D., Klarica M. (2008). Transventricular and Transpial Absorption of Cerebrospinal Fluid into Cerebral Microvessels. Coll. Antropol..

[37] Orešković D., Klarica M. (2014). A New Look at Cerebrospinal Fluid Movement. Fluids Barriers CNS.

[38] Yamada S., Miyazaki M., Yamashita Y., Ouyang C., Yui M., Nakahashi M., Shimizu S., Aoki I., Morohoshi Y., McComb J.G. (2013). Influence of Respiration on Cerebrospinal Fluid Movement Using Magnetic Resonance Spin Labeling. Fluids Barriers CNS.

[39] Xie L., Kang H., Xu Q., Chen M.J., Liao Y., Thiyagarajan M., O’Donnell J., Christensen D.J., Nicholson C., Iliff J.J. (2013). Sleep Drives Metabolite Clearance from the Adult Brain. Science.

[40] Lee D.-H., Torchetti M.K., Winker K., Ip H.S., Song C.-S., Swayne D.E. (2015). Intercontinental Spread of Asian-Origin H5N8 to North America through Beringia by Migratory Birds. J. Virol..

[41] Rasmussen M.K., Mestre H., Nedergaard M. (2018). The Glymphatic Pathway in Neurological Disorders. Lancet Neurol..

[42] Nedergaard M., Goldman S.A. (2020). Glymphatic Failure as a Final Common Pathway to Dementia. Science.

[43] Buccellato F.R., D’Anca M., Serpente M., Arighi A., Galimberti D. (2022). The Role of Glymphatic System in Alzheimer’s and Parkinson’s Disease Pathogenesis. Biomedicines.

[44] Huang S.-Y., Zhang Y.-R., Guo Y., Du J., Ren P., Wu B.-S., Feng J.-F., Cheng W., Yu J.-T. (2024). Alzheimer’s Disease Neuroimaging Initiative. Glymphatic System Dysfunction Predicts Amyloid Deposition, Neurodegeneration, and Clinical Progression in Alzheimer’s Disease. Alzheimers Dement..

[45] Silva I., Silva J., Ferreira R., Trigo D. (2021). Glymphatic System, AQP4, and Their Implications in Alzheimer’s Disease. Neurol. Res. Pract..

[46] Ni R. (2021). Magnetic Resonance Imaging in Animal Models of Alzheimer’s Disease Amyloidosis. Int. J. Mol. Sci..

[47] Keil S.A., Jansson D., Braun M., Iliff J.J. (2025). Glymphatic Dysfunction in Alzheimer’s Disease: A Critical Appraisal. Science.

[48] Simon M., Wang M.X., Ismail O., Braun M., Schindler A.G., Reemmer J., Wang Z., Haveliwala M.A., O’Boyle R.P., Han W.Y. (2022). Loss of Perivascular Aquaporin-4 Localization Impairs Glymphatic Exchange and Promotes Amyloid β Plaque Formation in Mice. Alzheimers Res. Ther..

[49] MacAulay N. (2021). Molecular Mechanisms of Brain Water Transport. Nat. Rev. Neurosci..

[50] Rossi A., Ratelade J., Papadopoulos M.C., Bennett J.L., Verkman A.S. (2012). Neuromyelitis Optica IgG Does Not Alter Aquaporin-4 Water Permeability, Plasma Membrane M1/M23 Isoform Content, or Supramolecular Assembly. Glia.

[51] Saini H., Fernandez G., Kerr D., Levy M. (2010). Differential Expression of Aquaporin-4 Isoforms Localizes with Neuromyelitis Optica Disease Activity. J. Neuroimmunol..

[52] Lien C.F., Hazai D., Yeung D., Tan J., Füchtbauer E.-M., Jancsik V., Górecki D.C. (2007). Expression of Alpha-Dystrobrevin in Blood-Tissue Barriers: Sub-Cellular Localisation and Molecular Characterisation in Normal and Dystrophic Mice. Cell Tissue Res..

[53] Neely J.D., Amiry-Moghaddam M., Ottersen O.P., Froehner S.C., Agre P., Adams M.E. (2001). Syntrophin-Dependent Expression and Localization of Aquaporin-4 Water Channel Protein. Proc. Natl. Acad. Sci. USA.

[54] Salman M.M., Kitchen P., Halsey A., Wang M.X., Törnroth-Horsefield S., Conner A.C., Badaut J., Iliff J.J., Bill R.M. (2022). Emerging Roles for Dynamic Aquaporin-4 Subcellular Relocalization in CNS Water Homeostasis. Brain.

[55] Nycz B., Mandera M. (2021). The Features of the Glymphatic System. Auton. Neurosci..

[56] Reith W., Haußmann A. (2018). Importance of Virchow-Robin spaces. Radiologe.

[57] Verkman A.S., Tradtrantip L., Smith A.J., Yao X. (2017). Aquaporin Water Channels and Hydrocephalus. Pediatr. Neurosurg..

[58] Kitchen P., Salman M.M., Halsey A.M., Clarke-Bland C., MacDonald J.A., Ishida H., Vogel H.J., Almutiri S., Logan A., Kreida S. (2020). Targeting Aquaporin-4 Subcellular Localization to Treat Central Nervous System Edema. Cell.

[59] Taya K., Marmarou C.R., Okuno K., Prieto R., Marmarou A. (2010). Effect of Secondary Insults upon Aquaporin-4 Water Channels Following Experimental Cortical Contusion in Rats. J. Neurotrauma.

[60] Hu H., Yao H., Zhang W., Zhang L., Ding W., Zhang S., Chen Z., Wei E. (2005). Increased Expression of Aquaporin-4 in Human Traumatic Brain Injury and Brain Tumors. J. Zhejiang Univ. Sci. B.

[61] Braun M., Sevao M., Keil S.A., Gino E., Wang M.X., Lee J., Haveliwala M.A., Klein E., Agarwal S., Pedersen T. (2024). Macroscopic Changes in Aquaporin-4 Underlie Blast Traumatic Brain Injury-Related Impairment in Glymphatic Function. Brain.

[62] Schmidt M.J., Rummel C., Hauer J., Kolecka M., Ondreka N., McClure V., Roth J. (2016). Increased CSF Aquaporin-4, and Interleukin-6 Levels in Dogs with Idiopathic Communicating Internal Hydrocephalus and a Decrease after Ventriculo-Peritoneal Shunting. Fluids Barriers CNS.

[63] Burg M.B., Ferraris J.D., Dmitrieva N.I. (2007). Cellular Response to Hyperosmotic Stresses. Physiol. Rev..

[64] Kushwah N., Jain V., Yadav D. (2020). Osmolytes: A Possible Therapeutic Molecule for Ameliorating the Neurodegeneration Caused by Protein Misfolding and Aggregation. Biomolecules.

[65] Mueed Z., Mehta D., Rai P.K., Kamal M.A., Poddar N.K. (2020). Cross-Interplay between Osmolytes and mTOR in Alzheimer’s Disease Pathogenesis. Curr. Pharm. Des..

[66] Khan S., Siraj S., Shahid M., Haque M.M., Islam A. (2023). Osmolytes: Wonder Molecules to Combat Protein Misfolding against Stress Conditions. Int. J. Biol. Macromol..

[67] Woo J., Kim J.E., Im J.J., Lee J., Jeong H.S., Park S., Jung S.-Y., An H., Yoon S., Lim S.M. (2018). Astrocytic Water Channel Aquaporin-4 Modulates Brain Plasticity in Both Mice and Humans: A Potential Gliogenetic Mechanism Underlying Language-Associated Learning. Mol. Psychiatry.

[68] Nilius B., Eggermont J., Voets T., Buyse G., Manolopoulos V., Droogmans G. (1997). Properties of Volume-Regulated Anion Channels in Mammalian Cells. Prog. Biophys. Mol. Biol..

[69] Parkerson K.A., Sontheimer H. (2004). Biophysical and Pharmacological Characterization of Hypotonically Activated Chloride Currents in Cortical Astrocytes. Glia.

[70] Han Y.-E., Kwon J., Won J., An H., Jang M.W., Woo J., Lee J.S., Park M.G., Yoon B.-E., Lee S.E. (2019). Tweety-Homolog (Ttyh) Family Encodes the Pore-Forming Subunits of the Swelling-Dependent Volume-Regulated Anion Channel (VRACswell) in the Brain. Exp. Neurobiol..

[71] Chen S., Wang H., Zhang L., Xi Y., Lu Y., Yu K., Zhu Y., Regina I., Bi Y., Tong F. (2025). Glymphatic System: A Self-Purification Circulation in Brain. Front. Cell Neurosci..

[72] Mestre H., Mori Y., Nedergaard M. (2020). The Brain’s Glymphatic System: Current Controversies. Trends Neurosci..

[73] Pasantes-Morales H., Cardin V., Tuz K. (2000). Signaling Events during Swelling and Regulatory Volume Decrease. Neurochem. Res..

[74] Pasantes-Morales H., Franco R., Torres-Marquez M.E., Hernández-Fonseca K., Ortega A. (2000). Amino Acid Osmolytes in Regulatory Volume Decrease and Isovolumetric Regulation in Brain Cells: Contribution and Mechanisms. Cell Physiol. Biochem..

[75] Pasantes-Morales H., Lezama R.A., Ramos-Mandujano G., Tuz K.L. (2006). Mechanisms of Cell Volume Regulation in Hypo-Osmolality. Am. J. Med..

[76] Pasantes-Morales H., Franco R., Ordaz B., Ochoa L.D. (2002). Mechanisms Counteracting Swelling in Brain Cells during Hyponatremia. Arch. Med. Res..

[77] Zhou Z., Zhan J., Cai Q., Xu F., Chai R., Lam K., Luan Z., Zhou G., Tsang S., Kipp M. (2022). The Water Transport System in Astrocytes-Aquaporins. Cells.

[78] Melton J.E., Patlak C.S., Pettigrew K.D., Cserr H.F. (1987). Volume Regulatory Loss of Na, Cl, and K from Rat Brain during Acute Hyponatremia. Am. J. Physiol..

[79] Verbalis J.G., Gullans S.R. (1991). Hyponatremia Causes Large Sustained Reductions in Brain Content of Multiple Organic Osmolytes in Rats. Brain Res..

[80] Sterns R.H., Baer J., Ebersol S., Thomas D., Lohr J.W., Kamm D.E. (1993). Organic Osmolytes in Acute Hyponatremia. Am. J. Physiol..

[81] Pasantes-Morales H., Cruz-Rangel S. (2010). Brain Volume Regulation: Osmolytes and Aquaporin Perspectives. Neuroscience.

[82] Lang F., Busch G.L., Ritter M., Völkl H., Waldegger S., Gulbins E., Häussinger D. (1998). Functional Significance of Cell Volume Regulatory Mechanisms. Physiol. Rev..

[83] Kopecká J., Krijt J., Raková K., Kožich V. (2011). Restoring Assembly and Activity of Cystathionine β-Synthase Mutants by Ligands and Chemical Chaperones. J. Inherit. Metab. Dis..

[84] Kempson S.A., Zhou Y., Danbolt N.C. (2014). The Betaine/GABA Transporter and Betaine: Roles in Brain, Kidney, and Liver. Front. Physiol..

[85] De Angelis E., Borghetti P., Passeri B., Cavalli V., Ferrari L., Andrani M., Martelli P., Saleri R. (2024). Hyperosmotic Stress Induces the Expression of Organic Osmolyte Transporters in Porcine Intestinal Cells and Betaine Exerts a Protective Effect on the Barrier Function. Biomedicines.

[86] de Angelis E., Petronini P.G., Borghetti P., Borghetti A.F., Wheeler K.P. (1999). Induction of Betaine-γ-Aminobutyric Acid Transport Activity in Porcine Chondrocytes Exposed to Hypertonicity. J. Physiol..

[87] Tanaka K., Masu M., Nakanishi S. (1990). Structure and Functional Expression of the Cloned Rat Neurotensin Receptor. Neuron.

[88] Gadea A., López-Colomé A.M. (2001). Glial Transporters for Glutamate, Glycine, and GABA: II. GABA Transporters. J. Neurosci. Res..

[89] Eulenburg V., Gomeza J. (2010). Neurotransmitter Transporters Expressed in Glial Cells as Regulators of Synapse Function. Brain Res. Rev..

[90] Lopez-Corcuera B., Liu Q.R., Mandiyan S., Nelson H., Nelson N. (1992). Expression of a Mouse Brain cDNA Encoding Novel Gamma-Aminobutyric Acid Transporter. J. Biol. Chem..

[91] Borden L.A., Smith K.E., Gustafson E.L., Branchek T.A., Weinshank R.L. (1995). Cloning and Expression of a Betaine/GABA Transporter from Human Brain. J. Neurochem..

[92] Burnham C.E., Buerk B., Schmidt C., Bucuvalas J.C. (1996). A Liver-Specific Isoform of the Betaine/GABA Transporter in the Rat: cDNA Sequence and Organ Distribution. Biochim. Biophys. Acta.

[93] Rasola A., Galietta L.J., Barone V., Romeo G., Bagnasco S. (1995). Molecular Cloning and Functional Characterization of a GABA/Betaine Transporter from Human Kidney. FEBS Lett..

[94] Nishimura T., Yagi R., Usuda M., Oda K., Yamazaki M., Suda S., Takahashi Y., Okazaki F., Sai Y., Higuchi K. (2014). System A Amino Acid Transporter SNAT2 Shows Subtype-Specific Affinity for Betaine and Hyperosmotic Inducibility in Placental Trophoblasts. Biochim. Biophys. Acta.

[95] Zhu X.-M., Ong W.-Y. (2004). A Light and Electron Microscopic Study of Betaine/GABA Transporter Distribution in the Monkey Cerebral Neocortex and Hippocampus. J. Neurocytol..

[96] Rowley N.M., Smith M.D., Lamb J.G., Schousboe A., White H.S. (2011). Hippocampal Betaine/GABA Transporter mRNA Expression Is Not Regulated by Inflammation or Dehydration Post-Status Epilepticus. J. Neurochem..

[97] Knight L.S., Piibe Q., Lambie I., Perkins C., Yancey P.H. (2017). Betaine in the Brain: Characterization of Betaine Uptake, Its Influence on Other Osmolytes and Its Potential Role in Neuroprotection from Osmotic Stress. Neurochem. Res..

[98] Ibi D., Tsuchihashi A., Nomura T., Hiramatsu M. (2019). Involvement of GAT2/BGT-1 in the Preventive Effects of Betaine on Cognitive Impairment and Brain Oxidative Stress in Amyloid β Peptide-Injected Mice. Eur. J. Pharmacol..

[99] Zhang Y., Ke Z., Luo J., Chen Q., Jiang X., Xiong J., Deng L. (2025). Betaine: A Promising Natural Product for Neurological and Psychiatric Diseases. Curr. Neuropharmacol..

[100] Santoro A., Buonocore M., D’Ursi A.M. (2025). Effect of Osmolytes on the Conformational Stability of Aβ(25-35): A Circular Dichroism Analysis. Biochim. Biophys. Acta Biomembr..

[101] Zhao H., Qu J., Li Q., Cui M., Wang J., Zhang K., Liu X., Feng H., Chen Y. (2018). Taurine Supplementation Reduces Neuroinflammation and Protects against White Matter Injury after Intracerebral Hemorrhage in Rats. Amino Acids.

[102] Amiraslani B., Sabouni F., Abbasi S., Nazem H., Sabet M. (2012). Recognition of Betaine as an Inhibitor of Lipopolysaccharide-Induced Nitric Oxide Production in Activated Microglial Cells. Iran. Biomed. J..

[103] Zhang Y., Jia J. (2023). Betaine Mitigates Amyloid-β-Associated Neuroinflammation by Suppressing the NLRP3 and NF-κB Signaling Pathways in Microglial Cells. J. Alzheimers Dis..

[104] Shi H., Wang X.-L., Quan H.-F., Yan L., Pei X.-Y., Wang R., Peng X.-D. (2019). Effects of Betaine on LPS-Stimulated Activation of Microglial M1/M2 Phenotypes by Suppressing TLR4/NF-κB Pathways in N9 Cells. Molecules.

[105] Kim H.Y., Kim H.V., Yoon J.H., Kang B.R., Cho S.M., Lee S., Kim J.Y., Kim J.W., Cho Y., Woo J. (2014). Taurine in Drinking Water Recovers Learning and Memory in the Adult APP/PS1 Mouse Model of Alzheimer’s Disease. Sci. Rep..

[106] Pukale D.D., Farrag M., Gudneppanavar R., Baumann H.J., Konopka M., Shriver L.P., Leipzig N.D. (2021). Osmoregulatory Role of Betaine and Betaine/γ-Aminobutyric Acid Transporter 1 in Post-Traumatic Syringomyelia. ACS Chem. Neurosci..

[107] Bhatt M., Di Iacovo A., Romanazzi T., Roseti C., Bossi E. (2023). Betaine-The Dark Knight of the Brain. Basic. Clin. Pharmacol. Toxicol..

[108] Buonaiuto G., Federiconi A., Vecchiato C.G., Benini E., Mordenti A.L. (2025). Betaine Dietary Supplementation: Healthy Aspects in Human and Animal Nutrition. Antioxidants.

[109] Jewell D.E., Tavener S.K., Creech R., Panickar K.S. (2024). Betaine and L-Carnitine Synergistically Influence the Metabolome and Immune Response in Dogs. Animals.

[110] Jewell D.E., Jackson M.I. (2022). Dietary Betaine and Fatty Acids Change Circulating Single-Carbon Metabolites and Fatty Acids in the Dog. Animals.

[111] Abu Ahmad N., Raizman M., Weizmann N., Wasek B., Arning E., Bottiglieri T., Tirosh O., Troen A.M. (2019). Betaine Attenuates Pathology by Stimulating Lipid Oxidation in Liver and Regulating Phospholipid Metabolism in Brain of Methionine-Choline-Deficient Rats. FASEB J..

[112] Ephraim E., Jewell D.E. (2020). Effect of Added Dietary Betaine and Soluble Fiber on Metabolites and Fecal Microbiome in Dogs with Early Renal Disease. Metabolites.

[113] Ko J.W., Lee Y., Jang Y., Kwon Y.H. (2024). Protective Effects of Taurine and Betaine against Neurotoxicity via Inhibition of Endoplasmic Reticulum Stress and Inflammation Signaling in the Brain of Mice Fed a Western Diet. J. Funct. Foods.

[114] Yang Z.-J., Huang S.-Y., Zhong K.-Y., Huang W.-G., Huang Z.-H., He T.-T., Yang M.-T., Wusiman M., Zhou D.-D., Chen S. (2024). Betaine Alleviates Cognitive Impairment Induced by Homocysteine through Attenuating NLRP3-Mediated Microglial Pyroptosis in an m6A-YTHDF2-Dependent Manner. Redox Biol..

[115] Pauline Rovers-Paap, Central Technical Manager, Orffa Additives, The Netherlands. The Unrivalled Benefit of Betaine in Pet Food. https://files.orffa.com/production/publications/ORF230605-Publication-Pet-Food-Supplement.pdf?dm=1762251666.

[116] Freeman L.M., Stern J.A., Fries R., Adin D.B., Rush J.E. (2018). Diet-Associated Dilated Cardiomyopathy in Dogs: What Do We Know?. J. Am. Vet. Med. Assoc..

[117] Furlanello T., Masti R., Bertolini F.M., Ongaro V., Zoia A., Sanchez Del Pulgar J. (2024). Development and Validation of a Robust and Straightforward LC-MS Method for Measuring Taurine in Whole Blood and Plasma of Dogs and Reference Intervals Calculation. Animals.

[118] Pion P.D., Kittleson M.D., Rogers Q.R., Morris J.G. (1987). Myocardial Failure in Cats Associated with Low Plasma Taurine: A Reversible Cardiomyopathy. Science.

[119] Wu J.-Y., Prentice H. (2010). Role of Taurine in the Central Nervous System. J. Biomed. Sci..

[120] Schaffer S., Kim H.W. (2018). Effects and Mechanisms of Taurine as a Therapeutic Agent. Biomol Ther.

[121] Hernández-Benítez R., Pasantes-Morales H., Saldaña I.T., Ramos-Mandujano G. (2010). Taurine Stimulates Proliferation of Mice Embryonic Cultured Neural Progenitor Cells. J. Neurosci. Res..

[122] Hernández-Benítez R., Ramos-Mandujano G., Pasantes-Morales H. (2012). Taurine Stimulates Proliferation and Promotes Neurogenesis of Mouse Adult Cultured Neural Stem/Progenitor Cells. Stem Cell Res..

[123] Pasantes-Morales H., Ramos-Mandujano G., Hernández-Benítez R. (2015). Taurine Enhances Proliferation and Promotes Neuronal Specification of Murine and Human Neural Stem/Progenitor Cells. Adv. Exp. Med. Biol..

[124] Miyamoto T.-A., Ueno T., Iguro Y., Yotsumoto G., Fukumoto Y., Nakamura K., Sakata R. (2009). Taurine-Mediated Cardioprotection Is Greater When Administered upon Reperfusion than Prior to Ischemia. Adv. Exp. Med. Biol..

[125] Terrill J.R., Pinniger G.J., Graves J.A., Grounds M.D., Arthur P.G. (2016). Increasing Taurine Intake and Taurine Synthesis Improves Skeletal Muscle Function in the Mdx Mouse Model for Duchenne Muscular Dystrophy. J. Physiol..

[126] Ahmadian M., Roshan V.D., Aslani E., Stannard S.R. (2017). Taurine Supplementation Has Anti-Atherogenic and Anti-Inflammatory Effects before and after Incremental Exercise in Heart Failure. Ther. Adv. Cardiovasc. Dis..

[127] Heidari R., Jamshidzadeh A., Niknahad H., Mardani E., Ommati M.M., Azarpira N., Khodaei F., Zarei A., Ayarzadeh M., Mousavi S. (2016). Effect of Taurine on Chronic and Acute Liver Injury: Focus on Blood and Brain Ammonia. Toxicol. Rep..

[128] Tognoni C.M., Biswas R.G., Suar Z.M., Carreras I., Dedeoglu A., Jenkins B.G. (2025). Protection of Multiple Aspects of Alzheimer’s Disease Pathology Using Dietary Supplementation with Taurine. Res. Sq..

[129] Huf F., Gutierres J.M., da Silva G.N., Zago A.M., Koenig L.F.C., Fernandes M.C. (2024). Neuroprotection Elicited by Taurine in Sporadic Alzheimer-like Disease: Benefits on Memory and Control of Neuroinflammation in the Hippocampus of Rats. Mol. Cell Biochem..

[130] Jang H., Lee S., Choi S.L., Kim H.Y., Baek S., Kim Y. (2017). Taurine Directly Binds to Oligomeric Amyloid-β and Recovers Cognitive Deficits in Alzheimer Model Mice. Adv. Exp. Med. Biol..

[131] Oh S.J., Lee H.-J., Jeong Y.J., Nam K.R., Kang K.J., Han S.J., Lee K.C., Lee Y.J., Choi J.Y. (2020). Evaluation of the Neuroprotective Effect of Taurine in Alzheimer’s Disease Using Functional Molecular Imaging. Sci. Rep..

[132] Xu S., He M., Zhong M., Li L., Lu Y., Zhang Y., Zhang L., Yu Z., Zhou Z. (2015). The Neuroprotective Effects of Taurine against Nickel by Reducing Oxidative Stress and Maintaining Mitochondrial Function in Cortical Neurons. Neurosci. Lett..

[133] Zhang B., Yang X., Gao X. (2010). Taurine Protects against Bilirubin-Induced Neurotoxicity in Vitro. Brain Res..

[134] Das J., Ghosh J., Manna P., Sinha M., Sil P.C. (2009). Taurine Protects Rat Testes against NaAsO(2)-Induced Oxidative Stress and Apoptosis via Mitochondrial Dependent and Independent Pathways. Toxicol. Lett..

[135] Baliou S., Adamaki M., Ioannou P., Pappa A., Panayiotidis M.I., Spandidos D.A., Christodoulou I., Kyriakopoulos A.M., Zoumpourlis V. (2021). Protective Role of Taurine against Oxidative Stress (Review). Mol. Med. Rep..

[136] Wu H., Jin Y., Wei J., Jin H., Sha D., Wu J.-Y. (2005). Mode of Action of Taurine as a Neuroprotector. Brain Res..

[137] Ripps H., Shen W. (2012). Review: Taurine: A “Very Essential” Amino Acid. Mol. Vis..

[138] Menzie J., Prentice H., Wu J.-Y. (2013). Neuroprotective Mechanisms of Taurine against Ischemic Stroke. Brain Sci..

[139] Kumari N., Prentice H., Wu J.-Y. (2013). Taurine and Its Neuroprotective Role. Adv. Exp. Med. Biol..

[140] Liu C., He P., Guo Y., Tian Q., Wang J., Wang G., Zhang Z., Li M. (2022). Taurine Attenuates Neuronal Ferroptosis by Regulating GABAB/AKT/GSK3β/β-Catenin Pathway after Subarachnoid Hemorrhage. Free Radic. Biol. Med..

[141] Li K., Wang D., Zhou X., Shao J., Li Y., Liu X., Zhang C., Zuo E., Shi X., Piao F. (2019). Taurine Protects Against Arsenic-Induced Apoptosis Via PI3K/Akt Pathway in Primary Cortical Neurons. Adv. Exp. Med. Biol..

[142] Ahmed S., Ma N., Kawanokuchi J., Matsuoka K., Oikawa S., Kobayashi H., Hiraku Y., Murata M. (2024). Taurine Reduces Microglia Activation in the Brain of Aged Senescence-Accelerated Mice by Increasing the Level of TREM2. Sci. Rep..

[143] Su Y., Fan W., Ma Z., Wen X., Wang W., Wu Q., Huang H. (2014). Taurine Improves Functional and Histological Outcomes and Reduces Inflammation in Traumatic Brain Injury. Neuroscience.

[144] Reeta K.H., Singh D., Gupta Y.K. (2017). Chronic Treatment with Taurine after Intracerebroventricular Streptozotocin Injection Improves Cognitive Dysfunction in Rats by Modulating Oxidative Stress, Cholinergic Functions and Neuroinflammation. Neurochem. Int..

[145] Aytan N., Choi J.-K., Carreras I., Brinkmann V., Kowall N.W., Jenkins B.G., Dedeoglu A. (2016). Fingolimod Modulates Multiple Neuroinflammatory Markers in a Mouse Model of Alzheimer’s Disease. Sci. Rep..

[146] Liu Q.R., López-Corcuera B., Mandiyan S., Nelson H., Nelson N. (1993). Molecular Characterization of Four Pharmacologically Distinct Gamma-Aminobutyric Acid Transporters in Mouse Brain [Corrected]. J. Biol. Chem..

[147] Ramamoorthy S., Leibach F.H., Mahesh V.B., Han H., Yang-Feng T., Blakely R.D., Ganapathy V. (1994). Functional Characterization and Chromosomal Localization of a Cloned Taurine Transporter from Human Placenta. Biochem. J..

[148] Kang Y.-S., Ohtsuki S., Takanaga H., Tomi M., Hosoya K.-I., Terasaki T. (2002). Regulation of Taurine Transport at the Blood-Brain Barrier by Tumor Necrosis Factor-Alpha, Taurine and Hypertonicity. J. Neurochem..

[149] Han X., Patters A.B., Jones D.P., Zelikovic I., Chesney R.W. (2006). The Taurine Transporter: Mechanisms of Regulation. Acta Physiol..

[150] Ramírez-Guerrero S., Guardo-Maya S., Medina-Rincón G.J., Orrego-González E.E., Cabezas-Pérez R., González-Reyes R.E. (2022). Taurine and Astrocytes: A Homeostatic and Neuroprotective Relationship. Front. Mol. Neurosci..

[151] Vitvitsky V., Garg S.K., Banerjee R. (2011). Taurine Biosynthesis by Neurons and Astrocytes. J. Biol. Chem..

[152] Hernández-Benítez R., Sedeño-Cortés A., Ramos-Mandujano G., Pasantes-Morales H. (2014). Regulatory Volume Decrease in Neural Precursor Cells: Taurine Efflux and Gene Microarray Analysis. Cell Physiol. Biochem..

[153] Olson J.E., Martinho E. (2006). Regulation of Taurine Transport in Rat Hippocampal Neurons by Hypo-Osmotic Swelling. J. Neurochem..

[154] Hackett M.J., Paterson P.G., Pickering I.J., George G.N. (2016). Imaging Taurine in the Central Nervous System Using Chemically Specific X-Ray Fluorescence Imaging at the Sulfur K-Edge. Anal. Chem..

[155] Pasantes Morales H., Schousboe A. (1988). Volume Regulation in Astrocytes: A Role for Taurine as an Osmoeffector. J. Neurosci. Res..

[156] Wu G. (2024). Roles of Nutrients in the Brain Development, Cognitive Function, and Mood of Dogs and Cats. Adv. Exp. Med. Biol..

[157] Milgram N.W., Zicker S.C., Head E., Muggenburg B.A., Murphey H., Ikeda-Douglas C.J., Cotman C.W. (2002). Dietary Enrichment Counteracts Age-Associated Cognitive Dysfunction in Canines. Neurobiol. Aging.

[158] Margolis R.U., Press R., Altszuler N., Stewart M.A. (1971). Inositol Production by the Brain in Normal and Alloxan-Diabetic Dogs. Brain Res..

[159] Lepore E., Lauretta R., Bianchini M., Mormando M., Di Lorenzo C., Unfer V. (2021). Inositols Depletion and Resistance: Principal Mechanisms and Therapeutic Strategies. Int. J. Mol. Sci..

[160] Uldry M., Ibberson M., Horisberger J.D., Chatton J.Y., Riederer B.M., Thorens B. (2001). Identification of a Mammalian H(+)-Myo-Inositol Symporter Expressed Predominantly in the Brain. EMBO J..

[161] Fu H., Li B., Hertz L., Peng L. (2012). Contributions in Astrocytes of SMIT1/2 and HMIT to Myo-Inositol Uptake at Different Concentrations and pH. Neurochem. Int..

[162] Dai G., Yu H., Kruse M., Traynor-Kaplan A., Hille B. (2016). Osmoregulatory Inositol Transporter SMIT1 Modulates Electrical Activity by Adjusting PI(4,5)P2 Levels. Proc. Natl. Acad. Sci. USA.

[163] Shaldubina A., Buccafusca R., Johanson R.A., Agam G., Belmaker R.H., Berry G.T., Bersudsky Y. (2007). Behavioural Phenotyping of Sodium-Myo-Inositol Cotransporter Heterozygous Knockout Mice with Reduced Brain Inositol. Genes Brain Behav..

[164] Paquette A.F., Carbone B.E., Vogel S., Israel E., Maria S.D., Patil N.P., Sah S., Chowdhury D., Kondratiuk I., Labhart B. (2023). The Human Milk Component Myo-Inositol Promotes Neuronal Connectivity. Proc. Natl. Acad. Sci. USA.

[165] Bizzarri M., Fuso A., Dinicola S., Cucina A., Bevilacqua A. (2016). Pharmacodynamics and Pharmacokinetics of Inositol(s) in Health and Disease. Expert. Opin. Drug Metab. Toxicol..

[166] Cantley L.C. (2002). The Phosphoinositide 3-Kinase Pathway. Science.

[167] Balla T. (2013). Phosphoinositides: Tiny Lipids with Giant Impact on Cell Regulation. Physiol. Rev..

[168] Berridge M.J. (2016). The Inositol Trisphosphate/Calcium Signaling Pathway in Health and Disease. Physiol. Rev..

[169] Voevodskaya O., Simmons A., Nordenskjöld R., Kullberg J., Ahlström H., Lind L., Wahlund L.-O., Larsson E.-M., Westman E. (2014). Alzheimer’s Disease Neuroimaging Initiative. The Effects of Intracranial Volume Adjustment Approaches on Multiple Regional MRI Volumes in Healthy Aging and Alzheimer’s Disease. Front. Aging Neurosci..

[170] Miller B.L., Moats R.A., Shonk T., Ernst T., Woolley S., Ross B.D. (1993). Alzheimer Disease: Depiction of Increased Cerebral Myo-Inositol with Proton MR Spectroscopy. Radiology.

[171] Haris M., Cai K., Singh A., Hariharan H., Reddy R. (2011). In Vivo Mapping of Brain Myo-Inositol. Neuroimage.

[172] Huang W., Alexander G.E., Daly E.M., Shetty H.U., Krasuski J.S., Rapoport S.I., Schapiro M.B. (1999). High Brain Myo-Inositol Levels in the Predementia Phase of Alzheimer’s Disease in Adults with Down’s Syndrome: A 1H MRS Study. Am. J. Psychiatry.

[173] Borghys H., Schwab A., Keppler B. (2024). Middle-Aged Dogs with Low and High Aβ CSF Concentrations Show Differences in Energy and Stress Related Metabolic Profiles in CSF. Heliyon.

[174] Ali F., Manzoor U., Bhattacharya R., Bansal A.K., Chandrashekharaiah K.S., Singh L.R., Saraswati S.M., Uversky V., Dar T.A. (2022). Brain Metabolite, Myo-Inositol, Inhibits Catalase Activity: A Mechanism of the Distortion of the Antioxidant Defense System in Alzheimer’s Disease. ACS Omega.

[175] Wang X., Zheng W. (2019). Ca^2+^ Homeostasis Dysregulation in Alzheimer’s Disease: A Focus on Plasma Membrane and Cell Organelles. FASEB J..

[176] Fenili D., Brown M., Rappaport R., McLaurin J. (2007). Properties of Scyllo-Inositol as a Therapeutic Treatment of AD-like Pathology. J. Mol. Med..

[177] Arar S., Haque M.A., Bhatt N., Zhao Y., Kayed R. (2024). Effect of Natural Osmolytes on Recombinant Tau Monomer: Propensity of Oligomerization and Aggregation. ACS Chem. Neurosci..

[178] Videen J.S., Michaelis T., Pinto P., Ross B.D. (1995). Human Cerebral Osmolytes during Chronic Hyponatremia. A Proton Magnetic Resonance Spectroscopy Study. J. Clin. Investig..

[179] Cotman C.W., Head E., Muggenburg B.A., Zicker S., Milgram N.W. (2002). Brain Aging in the Canine: A Diet Enriched in Antioxidants Reduces Cognitive Dysfunction. Neurobiol. Aging.

[180] Pan Y., Landsberg G., Mougeot I., Kelly S., Xu H., Bhatnagar S., Gardner C.L., Milgram N.W. (2018). Efficacy of a Therapeutic Diet on Dogs with Signs of Cognitive Dysfunction Syndrome (CDS): A Prospective Double Blinded Placebo Controlled Clinical Study. Front. Nutr..

[181] Pero M.E., Cortese L., Mastellone V., Tudisco R., Musco N., Scandurra A., D’Aniello B., Vassalotti G., Bartolini F., Lombardi P. (2019). Effects of a Nutritional Supplement on Cognitive Function in Aged Dogs and on Synaptic Function of Primary Cultured Neurons. Animals.

[182] Haake J., Meyerhoff N., Meller S., Twele F., Charalambous M., Wilke V., Volk H. (2023). Investigating Owner Use of Dietary Supplements in Dogs with Canine Cognitive Dysfunction. Animals.

[183] Milgram N.W., Head E., Zicker S.C., Ikeda-Douglas C.J., Murphey H., Muggenburg B., Siwak C., Tapp D., Cotman C.W. (2005). Learning Ability in Aged Beagle Dogs Is Preserved by Behavioral Enrichment and Dietary Fortification: A Two-Year Longitudinal Study. Neurobiol. Aging.

[184] Hein Z.M., Vishnumukkala T., Karikalan B., Alkatiri A., Hussan F., Jagadeesan S., Kamaruzzaman M.A., Che Ramli M.D., Che Mohd Nassir C.M.N., Gopalakrishna P.K. (2025). Autophagy and Alzheimer’s Disease: Mechanisms and Impact Beyond the Brain. Cells.

[185] Schütt T., Toft N., Berendt M. (2015). Cognitive Function, Progression of Age-Related Behavioral Changes, Biomarkers, and Survival in Dogs More Than 8 Years Old. J. Vet. Intern. Med..

[186] Youssef S.A., Capucchio M.T., Rofina J.E., Chambers J.K., Uchida K., Nakayama H., Head E. (2016). Pathology of the Aging Brain in Domestic and Laboratory Animals, and Animal Models of Human Neurodegenerative Diseases. Vet. Pathol..

[187] Scheltens P., De Strooper B., Kivipelto M., Holstege H., Chételat G., Teunissen C.E., Cummings J., van der Flier W.M. (2021). Alzheimer’s Disease. Lancet.

[188] Bekris L.M., Yu C.-E., Bird T.D., Tsuang D.W. (2010). Genetics of Alzheimer Disease. J. Geriatr. Psychiatry Neurol..

[189] Jansen I.E., Savage J.E., Watanabe K., Bryois J., Williams D.M., Steinberg S., Sealock J., Karlsson I.K., Hägg S., Athanasiu L. (2019). Genome-Wide Meta-Analysis Identifies New Loci and Functional Pathways Influencing Alzheimer’s Disease Risk. Nat. Genet..

[190] Kunkle B.W., Grenier-Boley B., Sims R., Bis J.C., Damotte V., Naj A.C., Boland A., Vronskaya M., van der Lee S.J., Amlie-Wolf A. (2019). Genetic Meta-Analysis of Diagnosed Alzheimer’s Disease Identifies New Risk Loci and Implicates Aβ, Tau, Immunity and Lipid Processing. Nat. Genet..

[191] Raichlen D.A., Alexander G.E. (2014). Exercise, APOE Genotype, and the Evolution of the Human Lifespan. Trends Neurosci..

[192] Yamazaki Y., Zhao N., Caulfield T.R., Liu C.-C., Bu G. (2019). Apolipoprotein E and Alzheimer Disease: Pathobiology and Targeting Strategies. Nat. Rev. Neurol..

[193] Parhizkar S., Holtzman D.M. (2022). APOE Mediated Neuroinflammation and Neurodegeneration in Alzheimer’s Disease. Semin. Immunol..

[194] Awano T., Johnson G.S., Wade C.M., Katz M.L., Johnson G.C., Taylor J.F., Perloski M., Biagi T., Baranowska I., Long S. (2009). Genome-Wide Association Analysis Reveals a SOD1 Mutation in Canine Degenerative Myelopathy That Resembles Amyotrophic Lateral Sclerosis. Proc. Natl. Acad. Sci. USA.

[195] Awano T., Katz M.L., O’Brien D.P., Sohar I., Lobel P., Coates J.R., Khan S., Johnson G.C., Giger U., Johnson G.S. (2006). A Frame Shift Mutation in Canine TPP1 (the Ortholog of Human CLN2) in a Juvenile Dachshund with Neuronal Ceroid Lipofuscinosis. Mol. Genet. Metab..

[196] Katz M.L., Farias F.H., Sanders D.N., Zeng R., Khan S., Johnson G.S., O’Brien D.P. (2011). A Missense Mutation in Canine CLN6 in an Australian Shepherd with Neuronal Ceroid Lipofuscinosis. J. Biomed. Biotechnol..

[197] Zeng R., Coates J.R., Johnson G.C., Hansen L., Awano T., Kolicheski A., Ivansson E., Perloski M., Lindblad-Toh K., O’Brien D.P. (2014). Breed Distribution of SOD1 Alleles Previously Associated with Canine Degenerative Myelopathy. J. Vet. Intern. Med..

[198] Melville S.A., Wilson C.L., Chiang C.S., Studdert V.P., Lingaas F., Wilton A.N. (2005). A Mutation in Canine CLN5 Causes Neuronal Ceroid Lipofuscinosis in Border Collie Dogs. Genomics.

[199] Yamato O., Ochiai K., Masuoka Y., Hayashida E., Tajima M., Omae S., Iijima M., Umemura T., Maede Y. (2000). GM1 Gangliosidosis in Shiba Dogs. Vet. Rec..

[200] Haskins M.E., Desnick R.J., DiFerrante N., Jezyk P.F., Patterson D.F. (1984). Beta-Glucuronidase Deficiency in a Dog: A Model of Human Mucopolysaccharidosis VII. Pediatr. Res..

[201] Kolicheski A., Johnson G.S., Villani N.A., O’Brien D.P., Mhlanga-Mutangadura T., Wenger D.A., Mikoloski K., Eagleson J.S., Taylor J.F., Schnabel R.D. (2017). GM2 Gangliosidosis in Shiba Inu Dogs with an In-Frame Deletion in HEXB. J. Vet. Intern. Med..

[202] Raj K., Berman-Booty L., Foureman P., Giger U. (2020). ARSB Gene Variants Causing Mucopolysaccharidosis VI in Miniature Pinscher and Miniature Schnauzer Dogs. Anim. Genet..

[203] Wrzosek M., Giza E., Płonek M., Podgórski P., Vandevelde M. (2015). Alexander Disease in a Dog: Case Presentation of Electrodiagnostic, Magnetic Resonance Imaging and Histopathologic Findings with Review of Literature. BMC Vet. Res..

[204] Selkoe D.J., Hardy J. (2016). The Amyloid Hypothesis of Alzheimer’s Disease at 25 Years. EMBO Mol. Med..

[205] Rofina J.E., van Ederen A.M., Toussaint M.J.M., Secrève M., van der Spek A., van der Meer I., Van Eerdenburg F.J.C.M., Gruys E. (2006). Cognitive Disturbances in Old Dogs Suffering from the Canine Counterpart of Alzheimer’s Disease. Brain Res..

[206] Landsberg G.M., Nichol J., Araujo J.A. (2012). Cognitive Dysfunction Syndrome: A Disease of Canine and Feline Brain Aging. Vet. Clin. N. Am. Small Anim. Pract..

[207] Walsh D.M., Selkoe D.J. (2007). A Beta Oligomers—A Decade of Discovery. J. Neurochem..

[208] Shankar G.M., Li S., Mehta T.H., Garcia-Munoz A., Shepardson N.E., Smith I., Brett F.M., Farrell M.A., Rowan M.J., Lemere C.A. (2008). Amyloid-Beta Protein Dimers Isolated Directly from Alzheimer’s Brains Impair Synaptic Plasticity and Memory. Nat. Med..

[209] Shen J., Kelleher R.J. (2007). The Presenilin Hypothesis of Alzheimer’s Disease: Evidence for a Loss-of-Function Pathogenic Mechanism. Proc. Natl. Acad. Sci. USA.

[210] Terry R.D., Masliah E., Salmon D.P., Butters N., DeTeresa R., Hill R., Hansen L.A., Katzman R. (1991). Physical Basis of Cognitive Alterations in Alzheimer’s Disease: Synapse Loss Is the Major Correlate of Cognitive Impairment. Ann. Neurol..

[211] Corder E.H., Saunders A.M., Strittmatter W.J., Schmechel D.E., Gaskell P.C., Small G.W., Roses A.D., Haines J.L., Pericak-Vance M.A. (1993). Gene Dose of Apolipoprotein E Type 4 Allele and the Risk of Alzheimer’s Disease in Late Onset Families. Science.

[212] Farrer L.A., Cupples L.A., Haines J.L., Hyman B., Kukull W.A., Mayeux R., Myers R.H., Pericak-Vance M.A., Risch N., van Duijn C.M. (1997). Effects of Age, Sex, and Ethnicity on the Association between Apolipoprotein E Genotype and Alzheimer Disease. A Meta-Analysis. APOE and Alzheimer Disease Meta Analysis Consortium. JAMA.

[213] Liu C.-C., Liu C.-C., Kanekiyo T., Xu H., Bu G. (2013). Apolipoprotein E and Alzheimer Disease: Risk, Mechanisms and Therapy. Nat. Rev. Neurol..

[214] Bu G. (2009). Apolipoprotein E and Its Receptors in Alzheimer’s Disease: Pathways, Pathogenesis and Therapy. Nat. Rev. Neurosci..

[215] Verghese P.B., Castellano J.M., Holtzman D.M. (2011). Apolipoprotein E in Alzheimer’s Disease and Other Neurological Disorders. Lancet Neurol..

[216] Haller S., Montandon M.-L., Rodriguez C., Ackermann M., Herrmann F.R., Giannakopoulos P. (2017). APOE*E4 Is Associated with Gray Matter Loss in the Posterior Cingulate Cortex in Healthy Elderly Controls Subsequently Developing Subtle Cognitive Decline. AJNR Am. J. Neuroradiol..

[217] Jack C.R., Shiung M.M., Gunter J.L., O’Brien P.C., Weigand S.D., Knopman D.S., Boeve B.F., Ivnik R.J., Smith G.E., Cha R.H. (2004). Comparison of Different MRI Brain Atrophy Rate Measures with Clinical Disease Progression in AD. Neurology.

[218] Castro-Fuentes R., Socas-Pérez R. (2025). Unveiling the Significance of Dog Domestication in Cognitive Dysfunction: Are Wolves Protected?. Open Vet. J..

[219] Dewey C.W., Davies E.S., Xie H., Wakshlag J.J. (2019). Canine Cognitive Dysfunction: Pathophysiology, Diagnosis, and Treatment. Vet. Clin. N. Am. Small Anim. Pract..

[220] Head E. (2013). A Canine Model of Human Aging and Alzheimer’s Disease. Biochim. Biophys. Acta.

[221] Haapasalo A., Kovacs D.M. (2011). The Many Substrates of Presenilin/γ-Secretase. J. Alzheimers Dis..

[222] Saura C.A., Choi S.-Y., Beglopoulos V., Malkani S., Zhang D., Shankaranarayana Rao B.S., Chattarji S., Kelleher R.J., Kandel E.R., Duff K. (2004). Loss of Presenilin Function Causes Impairments of Memory and Synaptic Plasticity Followed by Age-Dependent Neurodegeneration. Neuron.

[223] Lee J.-H., Yu W.H., Kumar A., Lee S., Mohan P.S., Peterhoff C.M., Wolfe D.M., Martinez-Vicente M., Massey A.C., Sovak G. (2010). Lysosomal Proteolysis and Autophagy Require Presenilin 1 and Are Disrupted by Alzheimer-Related PS1 Mutations. Cell.

[224] Sun L., Zhou R., Yang G., Shi Y. (2017). Analysis of 138 Pathogenic Mutations in Presenilin-1 on the in Vitro Production of Aβ42 and Aβ40 Peptides by γ-Secretase. Proc. Natl. Acad. Sci. USA.

[225] Tanzi R.E. (2012). The Genetics of Alzheimer Disease. Cold Spring Harb. Perspect. Med..

[226] Head E., Callahan H., Muggenburg B.A., Cotman C.W., Milgram N.W. (1998). Visual-Discrimination Learning Ability and Beta-Amyloid Accumulation in the Dog. Neurobiol. Aging.

[227] Weingarten M.D., Lockwood A.H., Hwo S.Y., Kirschner M.W. (1975). A Protein Factor Essential for Microtubule Assembly. Proc. Natl. Acad. Sci. USA.

[228] Cleveland D.W., Hwo S.Y., Kirschner M.W. (1977). Physical and Chemical Properties of Purified Tau Factor and the Role of Tau in Microtubule Assembly. J. Mol. Biol..

[229] Andreadis A., Brown W.M., Kosik K.S. (1992). Structure and Novel Exons of the Human Tau Gene. Biochemistry.

[230] Wang Y., Mandelkow E. (2016). Tau in Physiology and Pathology. Nat. Rev. Neurosci..

[231] Morris M., Maeda S., Vossel K., Mucke L. (2011). The Many Faces of Tau. Neuron.

[232] Goedert M., Spillantini M.G., Jakes R., Rutherford D., Crowther R.A. (1989). Multiple Isoforms of Human Microtubule-Associated Protein Tau: Sequences and Localization in Neurofibrillary Tangles of Alzheimer’s Disease. Neuron.

[233] Spillantini M.G., Goedert M. (2013). Tau Pathology and Neurodegeneration. Lancet Neurol..

[234] El Mammeri N., Dregni A.J., Duan P., Wang H.K., Hong M. (2022). Microtubule-Binding Core of the Tau Protein. Sci. Adv..

[235] Shi Y., Zhang W., Yang Y., Murzin A.G., Falcon B., Kotecha A., van Beers M., Tarutani A., Kametani F., Garringer H.J. (2021). Structure-Based Classification of Tauopathies. Nature.

[236] Smolek T., Madari A., Farbakova J., Kandrac O., Jadhav S., Cente M., Brezovakova V., Novak M., Zilka N. (2016). Tau Hyperphosphorylation in Synaptosomes and Neuroinflammation Are Associated with Canine Cognitive Impairment. J. Comp. Neurol..

[237] Buée L., Bussière T., Buée-Scherrer V., Delacourte A., Hof P.R. (2000). Tau Protein Isoforms, Phosphorylation and Role in Neurodegenerative Disorders. Brain Res. Brain Res. Rev..

[238] Petersen K.K., Milà-Alomà M., Li Y., Du L., Xiong C., Tosun D., Saef B., Saad Z.S., Du-Cuny L., Coomaraswamy J. (2026). Predicting Onset of Symptomatic Alzheimer’s Disease with Plasma p-Tau217 Clocks. Nat. Med..

[239] Samelson A.J., Ariqat N., McKetney J., Rohanitazangi G., Parra Bravo C., Bose R.S., Travaglini K.J., Lam V.L., Goodness D., Ta T. (2026). CRISPR Screens in iPSC-Derived Neurons Reveal Principles of Tau Proteostasis. Cell.

[240] Avila J., Lucas J.J., Perez M., Hernandez F. (2004). Role of Tau Protein in Both Physiological and Pathological Conditions. Physiol. Rev..

[241] Hernández F., Avila J. (2007). Tauopathies. Cell. Mol. Life Sci..

[242] Zempel H., Mandelkow E. (2014). Lost after Translation: Missorting of Tau Protein and Consequences for Alzheimer Disease. Trends Neurosci..

[243] von Bergen M., Friedhoff P., Biernat J., Heberle J., Mandelkow E.M., Mandelkow E. (2000). Assembly of Tau Protein into Alzheimer Paired Helical Filaments Depends on a Local Sequence Motif ((306)VQIVYK(311)) Forming Beta Structure. Proc. Natl. Acad. Sci. USA.

[244] Sawaya M.R., Sambashivan S., Nelson R., Ivanova M.I., Sievers S.A., Apostol M.I., Thompson M.J., Balbirnie M., Wiltzius J.J.W., McFarlane H.T. (2007). Atomic Structures of Amyloid Cross-Beta Spines Reveal Varied Steric Zippers. Nature.

[245] Fitzpatrick A.W.P., Falcon B., He S., Murzin A.G., Murshudov G., Garringer H.J., Crowther R.A., Ghetti B., Goedert M., Scheres S.H.W. (2017). Cryo-EM Structures of Tau Filaments from Alzheimer’s Disease. Nature.

[246] Braak H., Braak E. (1991). Neuropathological Stageing of Alzheimer-Related Changes. Acta Neuropathol..

[247] Tartaglia G.G., Pechmann S., Dobson C.M., Vendruscolo M. (2007). Life on the Edge: A Link between Gene Expression Levels and Aggregation Rates of Human Proteins. Trends Biochem. Sci..

[248] Baldwin A.J., Knowles T.P.J., Tartaglia G.G., Fitzpatrick A.W., Devlin G.L., Shammas S.L., Waudby C.A., Mossuto M.F., Meehan S., Gras S.L. (2011). Metastability of Native Proteins and the Phenomenon of Amyloid Formation. J. Am. Chem. Soc..

[249] Vecchi G., Sormanni P., Mannini B., Vandelli A., Tartaglia G.G., Dobson C.M., Hartl F.U., Vendruscolo M. (2020). Proteome-Wide Observation of the Phenomenon of Life on the Edge of Solubility. Proc. Natl. Acad. Sci. USA.

[250] Knowles T.P.J., Vendruscolo M., Dobson C.M. (2014). The Amyloid State and Its Association with Protein Misfolding Diseases. Nat. Rev. Mol. Cell Biol..

[251] Goedert M., Eisenberg D.S., Crowther R.A. (2017). Propagation of Tau Aggregates and Neurodegeneration. Annu. Rev. Neurosci..

[252] Sanders D.W., Kaufman S.K., DeVos S.L., Sharma A.M., Mirbaha H., Li A., Barker S.J., Foley A.C., Thorpe J.R., Serpell L.C. (2014). Distinct Tau Prion Strains Propagate in Cells and Mice and Define Different Tauopathies. Neuron.

[253] Zlokovic B.V. (2011). Neurovascular Pathways to Neurodegeneration in Alzheimer’s Disease and Other Disorders. Nat. Rev. Neurosci..

[254] Asai H., Ikezu S., Tsunoda S., Medalla M., Luebke J., Haydar T., Wolozin B., Butovsky O., Kügler S., Ikezu T. (2015). Depletion of Microglia and Inhibition of Exosome Synthesis Halt Tau Propagation. Nat. Neurosci..

[255] Giannakopoulos P., Herrmann F.R., Bussière T., Bouras C., Kövari E., Perl D.P., Morrison J.H., Gold G., Hof P.R. (2003). Tangle and Neuron Numbers, but Not Amyloid Load, Predict Cognitive Status in Alzheimer’s Disease. Neurology.

[256] Jack C.R., Bennett D.A., Blennow K., Carrillo M.C., Dunn B., Haeberlein S.B., Holtzman D.M., Jagust W., Jessen F., Karlawish J. (2018). NIA-AA Research Framework: Toward a Biological Definition of Alzheimer’s Disease. Alzheimers Dement..

[257] Busche M.A., Hyman B.T. (2020). Synergy between Amyloid-β and Tau in Alzheimer’s Disease. Nat. Neurosci..

[258] Roberson E.D., Scearce-Levie K., Palop J.J., Yan F., Cheng I.H., Wu T., Gerstein H., Yu G.-Q., Mucke L. (2007). Reducing Endogenous Tau Ameliorates Amyloid Beta-Induced Deficits in an Alzheimer’s Disease Mouse Model. Science.

[259] Thal D.R., Rüb U., Orantes M., Braak H. (2002). Phases of A Beta-Deposition in the Human Brain and Its Relevance for the Development of AD. Neurology.

[260] Ossenkoppele R., Schonhaut D.R., Schöll M., Lockhart S.N., Ayakta N., Baker S.L., O’Neil J.P., Janabi M., Lazaris A., Cantwell A. (2016). Tau PET Patterns Mirror Clinical and Neuroanatomical Variability in Alzheimer’s Disease. Brain.

[261] Greenberg S.M., Charidimou A. (2018). Diagnosis of Cerebral Amyloid Angiopathy: Evolution of the Boston Criteria. Stroke.

[262] Tarasoff-Conway J.M., Carare R.O., Osorio R.S., Glodzik L., Butler T., Fieremans E., Axel L., Rusinek H., Nicholson C., Zlokovic B.V. (2015). Clearance Systems in the Brain-Implications for Alzheimer Disease. Nat. Rev. Neurol..

[263] Smith E.E., Greenberg S.M. (2009). Beta-Amyloid, Blood Vessels, and Brain Function. Stroke.

[264] Carrano A., Hoozemans J.J.M., van der Vies S.M., Rozemuller A.J.M., van Horssen J., de Vries H.E. (2011). Amyloid Beta Induces Oxidative Stress-Mediated Blood-Brain Barrier Changes in Capillary Amyloid Angiopathy. Antioxid. Redox Signal..

[265] Attems J., Jellinger K.A. (2004). Only Cerebral Capillary Amyloid Angiopathy Correlates with Alzheimer Pathology--a Pilot Study. Acta Neuropathol..

[266] van Duinen S.G., Castaño E.M., Prelli F., Bots G.T., Luyendijk W., Frangione B. (1987). Hereditary Cerebral Hemorrhage with Amyloidosis in Patients of Dutch Origin Is Related to Alzheimer Disease. Proc. Natl. Acad. Sci. USA.

[267] Gómez-Isla T., Hollister R., West H., Mui S., Growdon J.H., Petersen R.C., Parisi J.E., Hyman B.T. (1997). Neuronal Loss Correlates with but Exceeds Neurofibrillary Tangles in Alzheimer’s Disease. Ann. Neurol..

[268] DeKosky S.T., Scheff S.W. (1990). Synapse Loss in Frontal Cortex Biopsies in Alzheimer’s Disease: Correlation with Cognitive Severity. Ann. Neurol..

[269] Bartus R.T., Dean R.L., Beer B., Lippa A.S. (1982). The Cholinergic Hypothesis of Geriatric Memory Dysfunction. Science.

[270] Francis P.T., Palmer A.M., Snape M., Wilcock G.K. (1999). The Cholinergic Hypothesis of Alzheimer’s Disease: A Review of Progress. J. Neurol. Neurosurg. Psychiatry.

[271] Perry E.K., Tomlinson B.E., Blessed G., Bergmann K., Gibson P.H., Perry R.H. (1978). Correlation of Cholinergic Abnormalities with Senile Plaques and Mental Test Scores in Senile Dementia. Br. Med. J..

[272] Insua D., Corredoira A., González-Martínez A., Suárez M.-L., Santamarina G., Sarasa M., Pesini P. (2012). Expression of P75(NTR), a Marker for Basal Forebrain Cholinergic Neurons, in Young and Aged Dogs with or without Cognitive Dysfunction Syndrome. J. Alzheimers Dis..

[273] Marien M.R., Colpaert F.C., Rosenquist A.C. (2004). Noradrenergic Mechanisms in Neurodegenerative Diseases: A Theory. Brain Res. Rev..

[274] Reddy P.H., Beal M.F. (2008). Amyloid Beta, Mitochondrial Dysfunction and Synaptic Damage: Implications for Cognitive Decline in Aging and Alzheimer’s Disease. Trends Mol. Med..

[275] Swerdlow R.H. (2018). Mitochondria and Mitochondrial Cascades in Alzheimer’s Disease. J. Alzheimers Dis..

[276] Smith M.A., Perry G., Richey P.L., Sayre L.M., Anderson V.E., Beal M.F., Kowall N. (1996). Oxidative Damage in Alzheimer’s. Nature.

[277] Butterfield D.A., Halliwell B. (2019). Oxidative Stress, Dysfunctional Glucose Metabolism and Alzheimer Disease. Nat. Rev. Neurosci..

[278] Heneka M.T., Carson M.J., El Khoury J., Landreth G.E., Brosseron F., Feinstein D.L., Jacobs A.H., Wyss-Coray T., Vitorica J., Ransohoff R.M. (2015). Neuroinflammation in Alzheimer’s Disease. Lancet Neurol..

[279] Akiyama H., Barger S., Barnum S., Bradt B., Bauer J., Cole G.M., Cooper N.R., Eikelenboom P., Emmerling M., Fiebich B.L. (2000). Inflammation and Alzheimer’s Disease. Neurobiol. Aging.

[280] Dias D., Socodato R. (2025). Beyond Amyloid and Tau: The Critical Role of Microglia in Alzheimer’s Disease Therapeutics. Biomedicines.

[281] Aspelund A., Antila S., Proulx S.T., Karlsen T.V., Karaman S., Detmar M., Wiig H., Alitalo K. (2015). A Dural Lymphatic Vascular System That Drains Brain Interstitial Fluid and Macromolecules. J. Exp. Med..

[282] Mansour G.K., Bolgova O., Hajjar A.W., Mavrych V. (2025). Neurovascular Dysfunction and Glymphatic Impairment: An Unexplored Therapeutic Frontier in Neurodegeneration. Int. J. Mol. Sci..

[283] Da Mesquita S., Louveau A., Vaccari A., Smirnov I., Cornelison R.C., Kingsmore K.M., Contarino C., Onengut-Gumuscu S., Farber E., Raper D. (2018). Functional Aspects of Meningeal Lymphatics in Ageing and Alzheimer’s Disease. Nature.

[284] Zeppenfeld D.M., Simon M., Haswell J.D., D’Abreo D., Murchison C., Quinn J.F., Grafe M.R., Woltjer R.L., Kaye J., Iliff J.J. (2017). Association of Perivascular Localization of Aquaporin-4 with Cognition and Alzheimer Disease in Aging Brains. JAMA Neurol..

[285] Valenza M., Facchinetti R., Steardo L., Scuderi C. (2019). Altered Waste Disposal System in Aging and Alzheimer’s Disease: Focus on Astrocytic Aquaporin-4. Front. Pharmacol..

[286] Ishida K., Yamada K., Nishiyama R., Hashimoto T., Nishida I., Abe Y., Yasui M., Iwatsubo T. (2022). Glymphatic System Clears Extracellular Tau and Protects from Tau Aggregation and Neurodegeneration. J. Exp. Med..

[287] Harrison I.F., Ismail O., Machhada A., Colgan N., Ohene Y., Nahavandi P., Ahmed Z., Fisher A., Meftah S., Murray T.K. (2020). Impaired Glymphatic Function and Clearance of Tau in an Alzheimer’s Disease Model. Brain.

[288] Zou W., Pu T., Feng W., Lu M., Zheng Y., Du R., Xiao M., Hu G. (2019). Blocking Meningeal Lymphatic Drainage Aggravates Parkinson’s Disease-like Pathology in Mice Overexpressing Mutated α-Synuclein. Transl. Neurodegener..

[289] Rainey-Smith S.R., Mazzucchelli G.N., Villemagne V.L., Brown B.M., Porter T., Weinborn M., Bucks R.S., Milicic L., Sohrabi H.R., Taddei K. (2018). Genetic Variation in Aquaporin-4 Moderates the Relationship between Sleep and Brain Aβ-Amyloid Burden. Transl. Psychiatry.

[290] Burfeind K.G., Murchison C.F., Westaway S.K., Simon M.J., Erten-Lyons D., Kaye J.A., Quinn J.F., Iliff J.J. (2017). The Effects of Noncoding Aquaporin-4 Single-Nucleotide Polymorphisms on Cognition and Functional Progression of Alzheimer’s Disease. Alzheimers Dement..

[291] Chandra A., Farrell C., Wilson H., Dervenoulas G., De Natale E.R., Politis M. (2021). Alzheimer’s Disease Neuroimaging Initiative Aquaporin-4 Polymorphisms Predict Amyloid Burden and Clinical Outcome in the Alzheimer’s Disease Spectrum. Neurobiol. Aging.

[292] Fang Y., Dai S., Jin C., Si X., Gu L., Song Z., Gao T., Chen Y., Yan Y., Yin X. (2021). Aquaporin-4 Polymorphisms Are Associated with Cognitive Performance in Parkinson’s Disease. Front. Aging Neurosci..

[293] Ringstad G., Vatnehol S.A.S., Eide P.K. (2017). Glymphatic MRI in Idiopathic Normal Pressure Hydrocephalus. Brain.

[294] Lee H.-J., Lee D.A., Shin K.J., Park K.M. (2022). Glymphatic System Dysfunction in Obstructive Sleep Apnea Evidenced by DTI-ALPS. Sleep Med..

[295] Ozsahin I., Zhou L., Wang X., Garetti J., Jamison K., Xi K., Tanzi E., Jaywant A., Patchell A., Maloney T. (2024). Diffusion Tensor Imaging Along Perivascular Spaces (DTI-ALPS) to Assess Effects of Age, Sex, and Head Size on Interstitial Fluid Dynamics in Healthy Subjects. J. Alzheimers Dis. Rep..

[296] Zhang W., Zhou Y., Wang J., Gong X., Chen Z., Zhang X., Cai J., Chen S., Fang L., Sun J. (2021). Glymphatic Clearance Function in Patients with Cerebral Small Vessel Disease. Neuroimage.

[297] Gold B.T., Shao X., Sudduth T.L., Jicha G.A., Wilcock D.M., Seago E.R., Wang D.J.J. (2021). Water Exchange Rate across the Blood-Brain Barrier Is Associated with CSF Amyloid-β 42 in Healthy Older Adults. Alzheimers Dement..

[298] Sudlow C., Gallacher J., Allen N., Beral V., Burton P., Danesh J., Downey P., Elliott P., Green J., Landray M. (2015). UK Biobank: An Open Access Resource for Identifying the Causes of a Wide Range of Complex Diseases of Middle and Old Age. PLoS Med..

[299] Jack C.R., Knopman D.S., Jagust W.J., Petersen R.C., Weiner M.W., Aisen P.S., Shaw L.M., Vemuri P., Wiste H.J., Weigand S.D. (2013). Tracking Pathophysiological Processes in Alzheimer’s Disease: An Updated Hypothetical Model of Dynamic Biomarkers. Lancet Neurol..

[300] Kopeć K., Koziorowski D., Szlufik S. (2025). The Therapeutic Potential of Glymphatic System Activity to Reduce the Pathogenic Accumulation of Cytotoxic Proteins in Alzheimer’s Disease. Int. J. Mol. Sci..

[301] Lilius T.O., Blomqvist K., Hauglund N.L., Liu G., Stæger F.F., Bærentzen S., Du T., Ahlström F., Backman J.T., Kalso E.A. (2019). Dexmedetomidine Enhances Glymphatic Brain Delivery of Intrathecally Administered Drugs. J. Control. Release.

[302] Aryal M., Azadian M.M., Hart A.R., Macedo N., Zhou Q., Rosenthal E.L., Airan R.D. (2022). Noninvasive Ultrasonic Induction of Cerebrospinal Fluid Flow Enhances Intrathecal Drug Delivery. J. Control. Release.

[303] Lilius T.O., Mortensen K.N., Deville C., Lohela T.J., Stæger F.F., Sigurdsson B., Fiordaliso E.M., Rosenholm M., Kamphuis C., Beekman F.J. (2023). Glymphatic-Assisted Perivascular Brain Delivery of Intrathecal Small Gold Nanoparticles. J. Control. Release.

[304] Ohara M., Hattori T. (2025). The Glymphatic System in Cerebrospinal Fluid Dynamics: Clinical Implications, Its Evaluation, and Application to Therapeutics. Neurodegener. Dis..

[305] Hyman B.T., Phelps C.H., Beach T.G., Bigio E.H., Cairns N.J., Carrillo M.C., Dickson D.W., Duyckaerts C., Frosch M.P., Masliah E. (2012). National Institute on Aging–Alzheimer’s Association Guidelines for the Neuropathologic Assessment of Alzheimer’s Disease. Alzheimers Dement..

[306] Whitehouse P.J., Price D.L., Struble R.G., Clark A.W., Coyle J.T., Delon M.R. (1982). Alzheimer’s Disease and Senile Dementia: Loss of Neurons in the Basal Forebrain. Science.

[307] Vinters H.V. (1987). Cerebral Amyloid Angiopathy. A Critical Review. Stroke.

[308] Arvanitakis Z., Capuano A.W., Leurgans S.E., Bennett D.A., Schneider J.A. (2016). Relation of Cerebral Vessel Disease to Alzheimer’s Disease Dementia and Cognitive Function in Elderly People: A Cross-Sectional Study. Lancet Neurol..

[309] Iqbal K., Liu F., Gong C.-X. (2016). Tau and Neurodegenerative Disease: The Story so Far. Nat. Rev. Neurol..

[310] Arriagada P.V., Growdon J.H., Hedley-Whyte E.T., Hyman B.T. (1992). Neurofibrillary Tangles but Not Senile Plaques Parallel Duration and Severity of Alzheimer’s Disease. Neurology.

[311] Attems J., Jellinger K., Thal D.R., Van Nostrand W. (2011). Review: Sporadic Cerebral Amyloid Angiopathy. Neuropathol. Appl. Neurobiol..

[312] Montine T.J., Phelps C.H., Beach T.G., Bigio E.H., Cairns N.J., Dickson D.W., Duyckaerts C., Frosch M.P., Masliah E., Mirra S.S. (2012). National Institute on Aging-Alzheimer’s Association Guidelines for the Neuropathologic Assessment of Alzheimer’s Disease: A Practical Approach. Acta Neuropathol..

[313] Tapp P.D., Siwak C.T., Gao F.Q., Chiou J.-Y., Black S.E., Head E., Muggenburg B.A., Cotman C.W., Milgram N.W., Su M.-Y. (2004). Frontal Lobe Volume, Function, and Beta-Amyloid Pathology in a Canine Model of Aging. J. Neurosci..

[314] Waller R., Baxter L., Fillingham D.J., Coelho S., Pozo J.M., Mozumder M., Frangi A.F., Ince P.G., Simpson J.E., Highley J.R. (2019). Iba-1-/CD68+ Microglia Are a Prominent Feature of Age-Associated Deep Subcortical White Matter Lesions. PLoS ONE.

[315] DeTure M.A., Dickson D.W. (2019). The Neuropathological Diagnosis of Alzheimer’s Disease. Mol. Neurodegener..

[316] MacQuiddy B., Moreno J.A., Kusick B., McGrath S. (2022). Assessment of Risk Factors in Dogs with Presumptive Advanced Canine Cognitive Dysfunction. Front. Vet. Sci..

[317] Pan Y., Kennedy A.D., Jönsson T.J., Milgram N.W. (2018). Cognitive Enhancement in Old Dogs from Dietary Supplementation with a Nutrient Blend Containing Arginine, Antioxidants, B Vitamins and Fish Oil. Br. J. Nutr..

[318] Ozawa M., Chambers J.K., Uchida K., Nakayama H. (2016). The Relation between Canine Cognitive Dysfunction and Age-Related Brain Lesions. J. Vet. Med. Sci..

[319] Ozawa M., Inoue M., Uchida K., Chambers J.K., Takeuch Y., Nakayama H. (2019). Physical Signs of Canine Cognitive Dysfunction. J. Vet. Med. Sci..

[320] Katina S., Farbakova J., Madari A., Novak M., Zilka N. (2016). Risk Factors for Canine Cognitive Dysfunction Syndrome in Slovakia. Acta Vet. Scand..

[321] Wrightson R., Albertini M., Pirrone F., McPeake K., Piotti P. (2023). The Relationship between Signs of Medical Conditions and Cognitive Decline in Senior Dogs. Animals.

[322] Vitturini C., Cerquetella M., Spaterna A., Bazzano M., Marchegiani A. (2025). Diagnosis of Canine Cognitive Dysfunction Syndrome: A Narrative Review. Vet. Sci..

[323] González-Martínez Á., Rosado B., Pesini P., Suárez M.-L., Santamarina G., García-Belenguer S., Villegas A., Monleón I., Sarasa M. (2011). Plasma β-Amyloid Peptides in Canine Aging and Cognitive Dysfunction as a Model of Alzheimer’s Disease. Exp. Gerontol..

[324] Pan Y. (2021). Nutrients, Cognitive Function, and Brain Aging: What We Have Learned from Dogs. Med. Sci..

[325] Dewey C.W., Rishniw M., Johnson P.J., Platt S., Robinson K., Sackman J., O’Donnell M. (2021). Canine Cognitive Dysfunction Patients Have Reduced Total Hippocampal Volume Compared with Aging Control Dogs: A Comparative Magnetic Resonance Imaging Study. Open Vet. J..

[326] Yoon J.-W., Nam C.-S., Lee K.-S., Dan T.-J., Jeon H.-J., Kang M.-A., Park H.-M. (2025). Evaluation of Blood-Based Diagnostic Biomarkers for Canine Cognitive Dysfunction Syndrome. Animals.

[327] Kang M.-H., Jeong W.-P., Nam C.-S., Yoon J.-W., Choi D.-M., Lee G.-S., Kim Y.-J., Dan T.-J., Park H.-M. (2025). Case Report: Ischemic Brain Infarction and Cognitive Dysfunction Syndrome in an Aged Dog. Front. Vet. Sci..

[328] Hasegawa D., Yayoshi N., Fujita Y., Fujita M., Orima H. (2005). Measurement of Interthalamic Adhesion Thickness as a Criteria for Brain Atrophy in Dogs with and without Cognitive Dysfunction (Dementia). Vet. Radiol. Ultrasound.

[329] Noh D., Choi S., Choi H., Lee Y., Lee K. (2017). Evaluation of Interthalamic Adhesion Size as an Indicator of Brain Atrophy in Dogs with and without Cognitive Dysfunction. Vet. Radiol. Ultrasound.

[330] van der Flier W.M., Skoog I., Schneider J.A., Pantoni L., Mok V., Chen C.L.H., Scheltens P. (2018). Vascular Cognitive Impairment. Nat. Rev. Dis. Primers.

[331] Chapagain D., Range F., Huber L., Virányi Z. (2018). Cognitive Aging in Dogs. Gerontology.

[332] Dewey C.W., da Costa R.C. (2015). Practical Guide to Canine and Feline Neurology.

[333] Hodshon A.W., Hecht S., Thomas W.B. (2014). Use of the T2*-Weighted Gradient Recalled Echo Sequence for Magnetic Resonance Imaging of the Canine and Feline Brain. Vet. Radiol. Ultrasound.

[334] Kerwin S.C., Levine J.M., Budke C.M., Griffin J.F., Boudreau C.E. (2017). Putative Cerebral Microbleeds in Dogs Undergoing Magnetic Resonance Imaging of the Head: A Retrospective Study of Demographics, Clinical Associations, and Relationship to Case Outcome. J. Vet. Intern. Med..

[335] Li Q., Yang Y., Reis C., Tao T., Li W., Li X., Zhang J.H. (2018). Cerebral Small Vessel Disease. Cell Transplant..

